# The impact of oxygen on the transcriptome of recombinant *S. cerevisiae *and *P. pastoris *- a comparative analysis

**DOI:** 10.1186/1471-2164-12-218

**Published:** 2011-05-09

**Authors:** Kristin Baumann, Laura Dato, Alexandra B Graf, Gianni Frascotti, Martin Dragosits, Danilo Porro, Diethard Mattanovich, Pau Ferrer, Paola Branduardi

**Affiliations:** 1 Department of Chemical Engineering, Autonomous University of Barcelona, Spain; 2 Department of Biotechnology and Bioscience, University of Milano-Bicocca, Milan, Italy; 3 Institute of Applied Microbiology, Department of Biotechnology, University of Natural Resources and Applied Life Sciences, Vienna, Austria; 4 School of Bioengineering, University of Applied Sciences, FH Campus Vienna, Austria; 5 Austrian Centre of Industrial Biotechnology (ACIB GmbH), Vienna, Austria; 6 Department of Computer Science, UC Davis Genome Center, University of California, Davis, USA

## Abstract

**Background:**

*Saccharomyces cerevisiae *and *Pichia pastoris *are two of the most relevant microbial eukaryotic platforms for the production of recombinant proteins. Their known genome sequences enabled several transcriptomic profiling studies under many different environmental conditions, thus mimicking not only perturbations and adaptations which occur in their natural surroundings, but also in industrial processes. Notably, the majority of such transcriptome analyses were performed using non-engineered strains.

In this comparative study, the gene expression profiles of *S. cerevisiae *and *P. pastoris*, a Crabtree positive and Crabtree negative yeast, respectively, were analyzed for three different oxygenation conditions (normoxic, oxygen-limited and hypoxic) under recombinant protein producing conditions in chemostat cultivations.

**Results:**

The major differences in the transcriptomes of *S. cerevisiae *and *P. pastoris *were observed between hypoxic and normoxic conditions, where the availability of oxygen strongly affected ergosterol biosynthesis, central carbon metabolism and stress responses, particularly the unfolded protein response. Steady state conditions under low oxygen set-points seemed to perturb the transcriptome of *S. cerevisiae *to a much lesser extent than the one of *P. pastoris*, reflecting the major tolerance of the baker's yeast towards oxygen limitation, and a higher fermentative capacity. Further important differences were related to Fab production, which was not significantly affected by oxygen availability in *S. cerevisiae*, while a clear productivity increase had been previously reported for hypoxically grown *P. pastoris*.

**Conclusions:**

The effect of three different levels of oxygen availability on the physiology of *P. pastoris *and *S. cerevisiae *revealed a very distinct remodelling of the transcriptional program, leading to novel insights into the different adaptive responses of Crabtree negative and positive yeasts to oxygen availability. Moreover, the application of such comparative genomic studies to recombinant hosts grown in different environments might lead to the identification of key factors for efficient protein production.

## Background

Yeasts are very well established host systems for the production of a broad number of recombinant proteins, and they are easy to manipulate and cultivate. During the last decades, many efforts have been made to tap their potential for improving foreign protein production for both commercial and academic purposes (for recent reviews, see [1,2]). Although a great number of such manipulations have been successful, these efforts were essentially based on the existing knowledge and empirical methods [3,4]. The initiation of the genomics era and the deciphering of the *Saccharomyces cerevisiae *genome in 1996 have enabled a significant progress in the identification of genes that were involved or activated in response to a wide range of different stresses [5,6], but only a few studies took advantage of the new systems biology tools to uncover novel helper genes to enhance heterologous protein secretion [3,7-9]. Until recently, even less effort has been made to combine such studies to investigate the impact of environmental stress responses (ESR) on recombinant protein production. Cells undergo continuous environmental fluctuations during industrial processes, triggering physiological responses and adaptations which are highly interrelated with correct protein folding and secretion. It is thus obvious that such studies - combining environmental stress responses under protein production conditions - would provide an important platform to identify targets for a rational engineering of not only yeasts but also other cellular protein factories.

The recently published sequence of the *Pichia pastoris *genome [10,11] allowed the development of *P. pastoris *specific microarrays [12] and the onset of genome-wide studies in this yeast expression system, which has been gaining high industrial relevance in the recent years. This advance was of special importance because the lack of host specific microarrays not only hindered research, but also implicated a certain dependency of data interpretation on similarities to *S. cerevisiae*. Recently, Dragosits and co-workers investigated the effects of temperature [13] and osmolarity [14] on the proteome/fluoxome and proteome/transcriptome, respectively, of a recombinant *P. pastoris *strain. Notably, the application of different levels of osmolarity to growing *P. pastoris *cells demonstrated differences in the physiological response as compared to published data for *S. cerevisiae*. Similarly, important differences between these yeasts have also been observed for the unfolded protein response (UPR) upon dithiothreitol (DTT) treatment and in a strain over-expressing the UPR transcription factor *HAC1 *[12]. These differences between data on *P. pastoris *and the established literature of *S. cerevisiae *point to less uniform regulatory systems in yeasts than believed until now.

The basis of this genome-wide comparative study were the previously analyzed transcriptional responses of recombinant *S. cerevisiae *(unpublished results) and *P. pastoris *[15] to oxygen availability. The engineered yeasts and their corresponding control (empty vector) strains were grown in normoxic, oxygen-limited and hypoxic conditions in glucose-limited chemostats. Both recombinant strains secreted a human antibody Fab fragment [16,17] under control of the constitutive glycolytic promoters pGAP (glyceraldehyde-3 phosphate dehydrogenase, for *P. pastoris*) and pTPI (triosephosphate isomerase, for *S. cerevisiae*).

Together with the relevance for industrial processes, where oxygen transfer is often described as important issue in high cell density fermentations, oxygen is of special interest in the comparison of two yeasts, both facultative anaerobe but with different capacities to ferment glucose (Crabtree effect). While *S. cerevisiae *is a highly fermentative Crabtree positive yeast, able to produce ethanol aerobically in the presence of high concentrations of glucose, *P. pastoris *is Crabtree negative and more sensitive to the availability of oxygen than to variations in substrate concentration.

Also, it is well known that environmental conditions have an effect on recombinant protein secretion, and that the profile and severity of such effect is host dependent. We are still far away from an ideal scenario of a universal expression system, like proposed by Sørensen [18]. However, a genome-wide approach aiming at the understanding of the global mechanisms connecting protein production to environmental conditions, as proposed in this study, could help to find strain or species dependent target genes or target mechanisms for cell engineering.

## Results and Discussion

### Physiological changes in yeasts under different levels of oxygen availability

The Fab expressing and control strains of *S. cerevisiae *and *P. pastoris *were grown in glucose-limited chemostat cultivations under normoxic (N), oxygen-limited (L) and hypoxic (H) conditions. Product yields of the secreted antibody Fab fragment, biomass concentration, and by-product concentrations during steady-state growth of all strains are given in Table 1. The most outstanding result was the different impact of hypoxic conditions on recombinant Fab secretion. While oxygen deprivation significantly increased the productivity of *P. pastoris *(data taken from [19]), the Fab yield in *S. cerevisiae *was not affected by oxygen.

**Table 1:** Physiological parameters

	YDM [g l^-1^]			Fab yield [mg_Fab3H6 _g_YDM_^-1^]			ethanol [g l^-1^]			arabitol*^a ^*or glycerol*^b ^*[g l^-1^]		
	
	N	L	H	N	L	H	N	L	H	N	L	H
*Sc*Fab3H6	9.01 ± 0.24	8.69 ± 0.19	5.88 ± 0.23	0.027 ± 0.005	0.029 ± 0.005	0.023 ± 0.004	nd	0.06 ± 0.017	3.64 ± 0.36	nd	nd	nd
*Sc *control	9.14 ± 0.52	8.59 ± 0.28	5.13 ± 0.31	-	-	-	nd	0.05 ± 0.007	3.46 ± 0.33	nd	nd	nd
*Pp*Fab3H6	23.98 ± 0.67	22.54 ± 1.06	12.58 ± 1.12	0.22 ± 0.01	0.38 ± 0.01	0.54 ± 0.02	nd	0.89 ± 0.09	6.85 ± 0.19	nd	0.90 ± 0.07	2.88 ± 0.18
*Pp *control	23.61 ± 0.77	20.14 ± 0.59	11.68 ± 0.28	-	-	-	nd	1.14 ± 0.12	5.75 ± 0.08	nd	1.21 ± 0.21	2.19 ± 0.26

Physiological characterization of the reference and recombinant strains of *S. cerevisiae *CEN.PK113 and *P. pastoris *X-33 in normoxic (N), oxygen-limited (L) and hypoxic (H) glucose-based chemostat cultivations. YDM = yeast dry mass, nd = not detected, *^a ^*in *P. pastoris *and *^b ^*in *S. cerevisiae*

### General overview of the transcriptional analysis

We performed a global analysis of microarray data from recombinant and reference strains of *S. cerevisiae *and *P. pastoris *grown under different oxygen availability levels. For pair wise comparisons of the oxygen conditions, the type of gene regulation (up- or downregulated) always refers to the lower oxygen set-point. We only used a data set of common genes (2891 genes, see Methods section for a detailed description and additional file 1 for the gene list). From this selected data set we identified 412 genes for *P. pastoris *and 196 genes for *S. cerevisiae *with a significantly different expression (log2 fold change threshold ≥ 0.59; *p*-value ≤ 0.05). The numbers of up- and downregulated genes were very equally distributed in both yeasts when comparing hypoxic with normoxic conditions, but pointed to a generally stronger regulation in *P. pastoris *in the other comparisons, with the exception of the control strain in oxygen-limited *vs*. normoxic conditions (see Table 2).

**Table 2:** Microarray statistics

	HvsN				HvsL				LvsN			
	***S. cerevisiae***		***P. pastoris***		***S. cerevisiae***		***P. pastoris***		***S. cerevisiae***		***P. pastoris***	
	
	**Cont**	**Fab**	**Cont**	**Fab**	**Cont**	**Fab**	**Cont**	**Fab**	**Cont**	**Fab**	**Cont**	**Fab**

up	69	75	70	86	25	35	91	145	2	3	0	22
down	82	83	110	107	2	10	114	112	10	10	0	30
% regulated	5.22	5.47	6.23	6.68	0.93	1.56	7.09	8.89	0.42	0.45	0.00	1.80

Microarray statistics including the common genes (2891) of *S. cerevisiae *and *P. pastoris*. Numbers reflect the regulated genes derived from pair wise comparisons between oxygen set-points, considering a *p*-value threshold ≤ 0.05 and a log2 fold change cut-off of 0.59. HvN = hypoxic *vs*. normoxic; HvL = hypoxic *vs*. oxygen-limited; LvN = oxygen-limited *vs*. normoxic; Cont = control strain; Fab = expressing strain

### Principal component analysis

**Figure 1:**
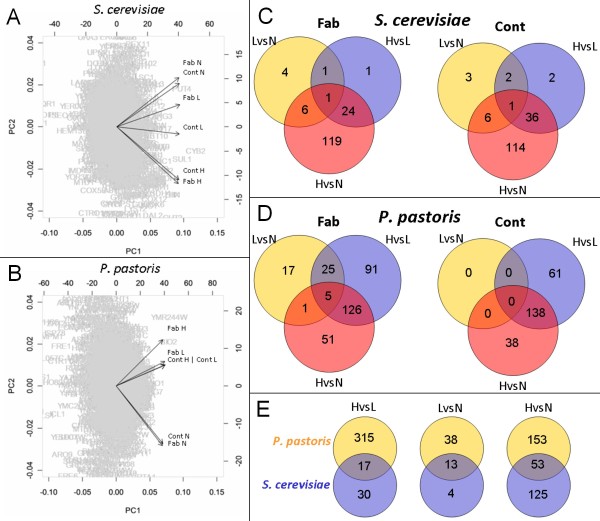
**Global comparison of differential gene regulation**. The correlation matrix of 2191 common genes (absolute normalized expressions levels) for (A) *S. cerevisiae *and (B) *P. pastoris *strains grown under normoxic (N), oxygen-limited (L) and hypoxic (H) conditions are visualized with the principle component analysis (PCA) biplot. Lines pointing in the same direction correspond to strains and oxygen conditions which are correlated. In both strains, the first and second components explain 97 % of the total variation. Overlapping sets of regulated genes in (C) *S. cerevisiae*, (D) *P. pastoris *and (E) between both species are displayed by Venn diagrams. Fab = Fab producing strain, Cont = control strain. HvsL = hypoxic *vs*. oxygen-limited, HvsN = hypoxic *vs*. normoxic, and LvsN = oxygen-limited *vs*. normoxic.

In a first approach, the absolute (normalized) expression data of the two yeast species were separately subjected to principal component analysis (PCA). The first and second component, accounting for 97 % of the total variability in the expression data, were plotted against each other and revealed oxygen as the major discriminating factor in both data sets (Figure 1A and 1B). Interestingly, the first principal component did not reveal any key parameter for the internal structure in our data. It was the second component that determined a correlation between oxygen availability and data distribution. We assume that this result is quite common in highly dimensional data like it is the case for microarray data since the first component represents a weighted average and distinguishes genes by their average overall expression [20]. PCA projection in the case of *S. cerevisiae *(Figure 1A) revealed a clear division between normoxic and hypoxic gene expression, with no difference regarding the strain genetic background (control or expressing strain). The clusters for oxygen-limited conditions were distributed along the PC2 axis, slightly separating the producing from the reference strain. The PCA for *P. pastoris *(Figure 1B) showed a similar behavior, since the second component was defined predominantly by oxygen, tightly grouping together hypoxic and oxygen-limited conditions. Only the producing strain in hypoxic conditions diverged from this classification and seemed to be the most influential variable. While the low oxygen 'environment' (hypoxia and oxygen limitation) in *P. pastoris *showed a positive correlation with the PC2, this interaction was negative in *S. cerevisiae *(and vice versa for the normoxic groups). Detailed analysis of the overlaps of regulated genes indicated that only few genes were differentially expressed between oxygen-limited and normoxic conditions in *S. cerevisiae*, while most regulation was apparent between hypoxic and normoxic conditions, almost fully overlapping with the set of genes that was differentially expressed between hypoxic and oxygen-limited conditions (Figure 1C). Differently, in *P. pastoris *the largest number of regulated genes appeared to be in the hypoxic *vs*. oxygen-limited comparison, overlapping with approximately 75 % of regulated genes between hypoxic and normoxic conditions (Figure 1D). While the transcriptomes of the *P. pastoris *control strain grown at limited and normal oxygen supply did not differ, there were 48 regulated genes in the Fab producing strain. The majority of differential regulation in *P. pastoris *emerged between hypoxic and oxygen-limited growth, while most regulation in *S. cerevisiae *was observed between hypoxic and normoxic growth (Figure 1E). These first results of the comparative analysis already pointed to major differences between the transcriptome of *P. pastoris *and *S. cerevisiae *in response to oxygen availability.

### Gene ontology group representation

**Figure 2:**
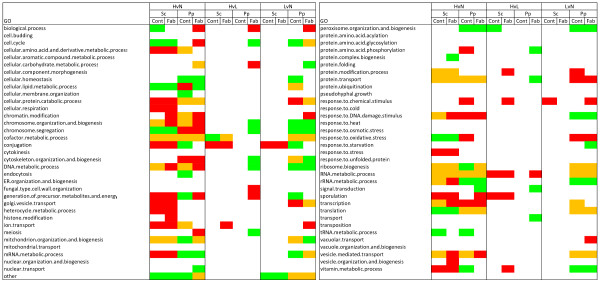
**GO group enrichment as determined by Fisher's exact test**. Significantly enriched GO functional groups (computed with the Fisher's exact test with a p-value ≤ 0.05) are labeled in red (upregulated genes), green (downregulated genes) and orange (both up- and downregulated genes). Pairwise comparisons in *S. cerevisiae *(Sc) and *P. pastoris *(Pp) are abbreviated as in figure 1.

Overrepresentation of up- and downregulated genes in gene ontology (GO) functional groups was evaluated by a Fisher's exact test (*p*-value ≤ 0.05). These enriched functional groups were analyzed with the fold change values of the pair wise comparisons, i.e. hypoxic *vs*. normoxic (HvsN), hypoxic *vs*. oxygen-limited (HvsL), and oxygen-limited *vs*. normoxic conditions (LvsN). The results are illustrated in Figure 2. While the comparison of hypoxic and normoxic conditions yielded a more extensive set of regulated genes, the number of enriched groups in the intermediate set points was generally more moderate, in particular for *S. cerevisiae*. While no statistically enriched GO terms were found for the *P. pastoris *reference strain comparing the lower oxygen conditions (HvsL), the extent of responsive GO categories for LvsN was similar to that in HvsN. These results were not only in good accordance with the PCA and direct comparison of equally regulated genes, but also with the different fermentative properties of the yeasts. Even though *P. pastoris *is facultative anaerobe, it has a higher propensity for respiratory growth than *S. cerevisiae*. This might explain a less drastic reorganization of the *S. cerevisiae *transcriptome after a shift to a reduced oxygen environment.

In case of the comparison hypoxic *vs*. fully aerobic, differences between the yeast species could be observed for the GO terms *cell cycle, chromosome segregation, response to oxidative stress *and *translation*. These four groups were enriched with downregulated genes (green) in *S. cerevisiae *and upregulated genes (red) in *P. pastoris*. For the functional categories *cellular homeostasis, amino acid metabolic process, protein catabolic process, conjugation, mRNA metabolic process, peroxisome organization and biogenesis, response to stress *and *vitamin metabolic process*, a significant number of genes were either downregulated in *P. pastoris*, and/or genes with a positive fold change were overrepresented in *S. cerevisiae*. Patterns of similarly regulated GO groups in the two yeast species could be observed for *cofactor metabolic process, protein transport *and *RNA metabolic process*, all of them comprising gene sets with both-side directed regulation (orange). A strain-dependent behavior (control *vs*. expressing) was recognized for the GO categories *chromosome organization and biogenesis, DNA metabolic process, vesicle mediated transport *and *tRNA metabolic process*. While the first three groups were upregulated in the expressing strains and bidirectional in the reference strains, the *tRNA metabolic process *was significantly downregulated in the control strains. Additionally, for the categories *cellular lipid metabolic process, generation of precursor metabolites and energy, mitochondrion organization and biogenesis *and *ribosome biogenesis *the behaviour of both *S. cerevisiae *strains (recombinant and control) and of the *P. pastoris *Fab-expressing strain was similar, but different in the *P. pastoris *control strain.

### Omics Expression Viewer

**Figure 3:**
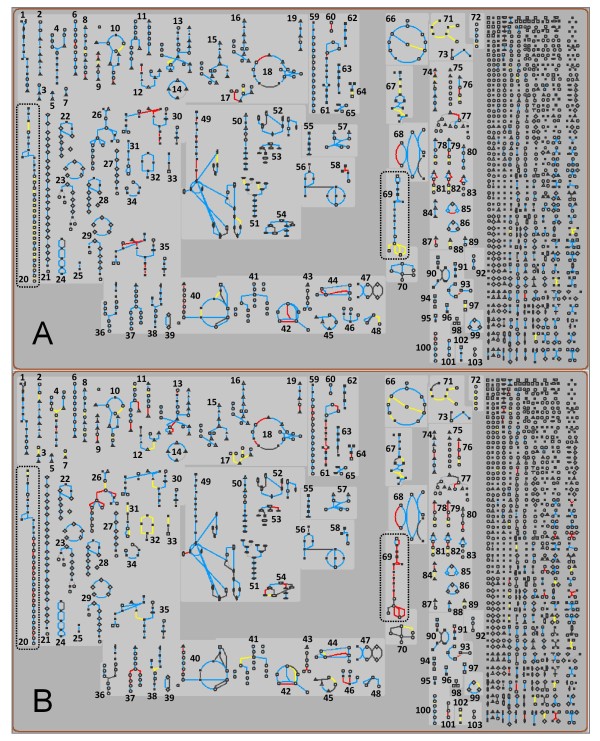
**Overlay of transcriptome data on the *S. cerevisiae *metabolic map**. Fold change data of the pairwise comparison hypoxic *vs*. normoxic (HvsN) of the recombinant *S. cerevisiae *(A) and *P. pastoris *(B) strain are overlapped with the metabolic map of *S. cerevisiae *(MetaCyc, SGD database [66]). Each node in the diagram represents a single metabolite, and each line represents a single bioreaction. In the right part of the diagram the small molecule metabolism is represented (for a complete description of the map see http://pathway.yeastgenome.org). Reaction lines are colour-coded (three colour bins) according to the fold change value of the gene: red for data values that exceed a log2 fold change threshold of 0.59, yellow for data values less than the inverse of the threshold, and blue for values in between. Detailed lists of all the regulated pathways, together with their diagrams and corresponding gene lists are provided in additional file 2 (for *S. cerevisiae*) and 3 (for *P. pastoris*). Depicted pathways are indicated by numbers, according to the table shown in additional file 4. The ergosterol pathway (n.20) and glycolysis (n.69) are indicated by dashed boxes.

To obtain a general view of metabolic pathways responding to oxygen availability, we overlapped the fold change values obtained from the HvsN comparison of the producing strains with the map of *S. cerevisiae *core metabolism. Figure 3 shows the overview of the entire schematized map, while detailed lists of all the regulated pathways, together with their diagrams and corresponding gene lists, are provided in additional file 2 (for *S. cerevisiae*) and additional file 3 (for *P. pastoris*). All depicted pathways are indicated by numbers according to the table shown in additional file 4. The most striking differences were detected for the glycolytic pathway, ergosterol and sphingolipid biosynthesis, and the oxidative branch of the pentose phosphate pathway (Figure 3). The uniform upregulation of glycolytic genes, enzymes and metabolic fluxes in *P. pastoris *chemostats upon a shift to hypoxic growth conditions was recently reported in our preceding study [15], indicating a transcriptional control of the central carbon metabolism in this yeast. In hypoxically grown *S. cerevisiae *this picture was quite different, since glycolysis was not regulated at the transcriptome level. These observations confirm previous chemostat studies, where a poor correlation between the mRNA levels and the corresponding protein abundances or *in vivo *fluxes demonstrated a post-transcriptional control of glycolysis in anaerobic *S. cerevisiae *cultures [21-23]. De Groot [22] further estimated the pool of glycolytic enzymes to account for 21 % of the total protein in anaerobic conditions, thus occupying a considerable fraction of the *S. cerevisiae *translation machinery. This 'occupation' could somehow hamper or limit the translation of other proteins, e.g. the recombinant Fab antibody. Estimation of the corresponding percentage in *P. pastoris *might provide new insights on the different production abilities of the two microorganisms.

An interesting observation was the opposite regulation of members of the ergosterol biosynthesis pathway. Ergosterol is an essential component of membrane lipids and regulates the fluidity and permeability of the plasma membrane, where it is most abundant. It has a broad function in cellular processes like plasma membrane fusion, pheromone signaling or protein sorting [24-26]. Furthermore, ergosterol biosynthesis is an oxygen-requiring process. In *P. pastoris *the majority of genes whose products catalyze oxygen-dependent reactions of this pathway (*ERG1, ERG11, ERG25 *and *ERG3*) were strongly upregulated in hypoxia, while *ERG27, ERG6 *and *ERG4 *were downregulated. In contrast, all the mentioned genes resulted to be downregulated in *S. cerevisiae*. We recently hypothesized that the upregulation of the genes catalyzing oxygen-consuming reactions in the ergosterol pathway of *P. pastoris *may be a reaction to intermediate or end product deficit provoked by low oxygen availability [15], since the ergosterol content was shown to be reduced under hypoxic conditions in a parallel study [27]. A similar trend was described for *S. cerevisiae *[28,29]. It has been assumed that the anaerobic/hypoxic induction of genes catalyzing oxygen-dependent reactions in some cases could be an adjustment of cells to subsequent oxygenation of the cultivations. In the case of *S. cerevisiae*, the ergosterol regulatory process may depend on the sterol requirement for growth and on the presence of other lipids, as postulated by Rosenfeld and Beauvoit [30]. In this context we assume that the sterol content in *S. cerevisiae *under hypoxic conditions was still sufficient to maintain growth. Interestingly, Rintala and co-workers [31] observed a similar downregulation of *ERG11, ERG25 *and *ERG6 *with 2.8% oxygen (in the inlet air) when compared to full aeration, but unchanged levels of *ERG1, ERG3 *and *ERG27*. Unlike in this work, however, their growth medium was supplemented throughout the study with ergosterol and unsaturated fatty acids, which are typically supplied only to anaerobic *S. cerevisiae *cultivation media in order to sustain growth [32,33]. Since endogenous or exogenous sterols are regulators of ERG gene expression, the provision of exogenous ergosterol in such experiments might have partially masked the "authentic" hypoxic response of *S. cerevisiae *and could explain the observed dissimilarities. In addition, transcriptional regulation could be directly influenced by oxygen concentration. Bunn and Poyton [34] demonstrated that different genes responding to the presence of oxygen have different thresholds for activation/deactivation of their transcription. In this view, it could be possible to imagine that different thresholds are sensed in the two yeasts, consistently with their different fermentation capacities. Therefore, a condition of oxygen limitation might be sensed by *P. pastoris *as more extreme, eliciting a proper response.

In relation to protein secretion, there are some indications that lipid metabolism, especially with regard to ergosterol biosynthesis and membrane remodeling, may affect protein secretion. This pathway was discussed in the recent work of our group as possible target for strain engineering, since it encountered a very strong reorganization during hypoxic conditions in which the secretion of the antibody Fab fragment was significantly improved [15]. Recent findings in our lab showed that disequilibrium of the membrane properties by applying non-ionic detergents or gene specific antifungal drugs stimulated recombinant protein secretion in *P. pastoris *shake flask cultures (Baumann et al., submitted). These findings strongly support our hypothesis about a link between changes in membrane fluidity and recombinant protein secretion in *P. pastoris*. Nonetheless, although an increased membrane fluidity was demonstrated for *erg *mutants of *S. cerevisiae *with defective ergosterol biosynthesis [35], such kinds of mutants were reported to be affected in the sorting of proteins to the plasma membrane [36] and in protein internalization via endocytosis [37] but not in protein secretion [36,37]. Therefore, it might be that differences in the membrane composition due to ergosterol limitation and/or compensation mechanisms do not have the same influence on protein secretion mechanisms in the two yeasts, thus contributing to the different secretion capacity observed in hypoxic conditions.

Transcription of the pentose phosphate pathway (PPP) genes was significantly reduced in both yeasts, but to a greater extent in *S. cerevisiae*. While in *P. pastoris *only genes of the non-oxidative pathway were regulated (*RKI1, TKL1 *and *TAL1*), in *S. cerevisiae *this list was supplemented by genes from the oxidative PPP branch including *SOL3, SOL4, GND1, GND2*. It was previously described that the distribution of carbon flux between glycolysis and PPP seems to be associated with the ability of yeasts to perform aerobic fermentation (Crabtree effect) [38,39]. *S. cerevisiae *as a Crabtree positive yeast was shown to have rather low metabolic fluxes through the pentose phosphate pathway [40] which is predominantly used for NADPH production and less directly for intermediates production. The downregulation of the pathway under hypoxic conditions may prevent an imbalance in the cellular redox system, since reducing equivalents like NADH or NADPH are accumulated in anaerobic cultivations. While *S. cerevisiae *typically produces glycerol in order to re-oxidize excess NADH [41], *P. pastoris *was recently shown to circumvent redox imbalances by the production of arabitol [27]. This observation could give reason for the different regulation, since D-ribulose and D-xylulose from the upper (oxidative) PPP are the main precursors for the formation of arabitol in many fungi [42,43].

### Hierarchical clustering

**Figure 4:**
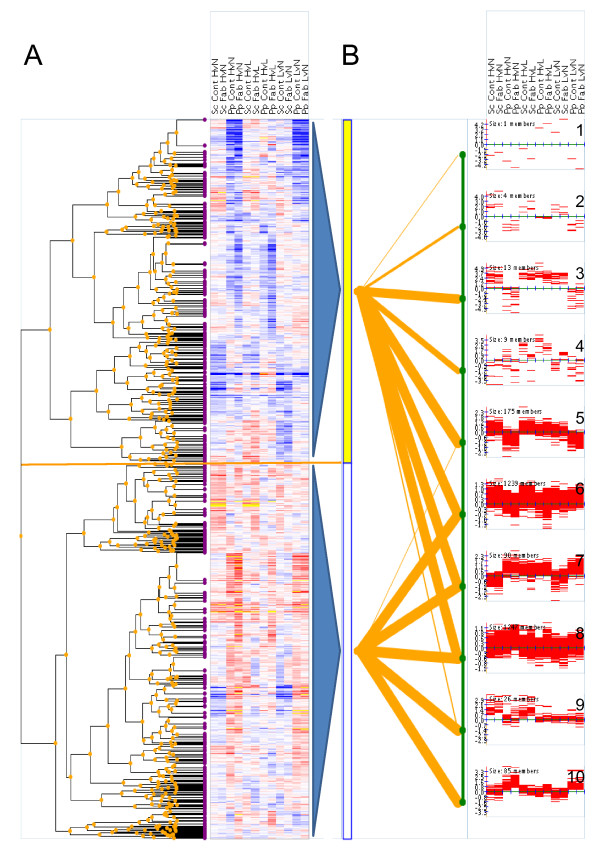
**Clustering comparison graphical output**. The output of the hierarchical clustering (correlation based distance, average linkage) of the fold changes of all pairwise comparisons of oxygen provision is displayed in form of a dendrogram attached to a heat map, with the two main branches indicated by a red line (A). The clustered matrix is linked to the outcome (line-plots) of the k-means clustering (k = 10) (B). The clustering comparison correspondence is displayed in the central part of the graph. It is depicted as lines of varying thickness, mapping sub-branches of the tree to flat clustering superclusters. Line thickness is proportional to the number of elements common to both sets. Pairwise comparisons in *S. cerevisiae *(Sc) and *P. pastoris *(Pp) are abbreviated as follows: HvsL (hypoxic *vs*. oxygen-limited), HvsN (hypoxic *vs*. normoxic) and LvsN (oxygen-limited *vs*. normoxic). Fab = Fab producing strain, Cont = control strain.

Another approach towards a global overview of the yeast's transcriptome in response to oxygen provision included cluster analysis (EBI Expression Profiler) with the fold change data as input files. Figure 4 illustrates the result of a clustering comparison, which linked the dendrogram of a hierarchical clustering to a set of corresponding flat (k-means) clusters (number of clusters = 10). The outcome demonstrated 7 interesting clusters (see discussion below) with notable differences between yeast species comparing hypoxic and normoxic conditions, and 2 clusters (6 and 8) with a very similar profile for the pair wise oxygen comparisons and yeast species. These clusters were also the two largest in size, representing 1239 and 1247 genes, respectively, on a small-scale y-axis (log2 values ranging between ± 1.3). Individual clusters were selected for further analysis and are discussed below. A list of all cluster members can be found in the additional file 5.

#### Clusters of interest

Cluster 2 comprised 4 mating related genes (*FIG1, KAR4, SST2 *and *STE2*), which were strongly upregulated in all pair wise comparisons in *S. cerevisiae*, and downregulated in *P. pastoris*. This result may reflect the different life cycle of the yeasts, which alternates between haplophase and diplophase in *S. cerevisiae*, while *P. pastoris *is most stable in its vegetative haploid state. In *S. cerevisiae*, mating occurs between two haploid cells of opposite mating type MATa or MATα (reviewed in [44]) after pheromone signalling, resulting in a diploid nucleus. In *P. pastoris *mating occurs only in conditions of severe nitrogen limitation [45].

The expression pattern in Cluster 3 (13 members) showed a species dependent regulation and had its strongest impact on the transcriptome of *P. pastoris *comparing hypoxic *vs*. normoxic and oxygen-limited *vs*. normoxic conditions. Among the most regulated genes we found 4 peroxisomal genes (*PCD1, PEX11, PEX13 *and the glyoxylate cycle involved gene *MDH3*) and 2 genes that are related to glycerol metabolism and transport (*GUT1, YFL054C*). *S. cerevisiae *may have a higher tolerance for oxygen deficiency by maintaining peroxisomal activities and restoring biosynthetic intermediates via anaplerotic reactions (e.g. glyoxylate cycle) [46]. On the other hand, carbon source may impact regulation of peroxisomal genes in the methylotrophic yeast *P. pastoris*. Since peroxisomes harbour the enzymes for methanol assimilation, these organelles drastically increase in size during growth on methanol, but degrade rapidly upon a shift to glucose [47]. This would, however, only explain a general low transcriptional level of peroxisome-related genes in *P. pastoris *grown on glucose, but not a downregulation under hypoxic and oxygen-limited conditions. We therefore assume an implication of other oxygen-related processes, e.g. the breakdown of fatty-acids, which is drastically reduced when oxygen is scarce (see cluster 4). *GUT1*, whose expression is induced on non-fermentable carbon sources such as glycerol or ethanol, and *YFL054C*, which encodes a putative channel-like protein that mediates passive diffusion of glycerol in the presence of ethanol, were slightly induced in *S. cerevisiae *at low oxygen availability. Since glycerol is generated in anaerobic cultures for the purpose of re-establishing the cell's redox balance, the upregulation of these genes is reasonable.

Further genes found in this cluster were two iron-related genes (the transcription factor *AFT1 *and the iron transporter *SIT1*), the a-mating factor receptor *STE3*, and a cell wall mannoprotein which is required for growth under anaerobic conditions (*TIR3*).

Cluster 4 contained 6 fatty acid β-oxidation pathway related genes (*ECI1, FAA2, FOX2, POX1, SPS19 *and *PXA1*). The transcript pattern, with a similar trend in both yeast species, indicated a strong downregulation in the set-points with low oxygen availability, but a slight upregulation in the comparison HvL, that is, showing their lowest gene expression levels under oxygen-limited rather than hypoxic conditions. Interestingly, the transcript levels were generally lower in the Fab-producing strains.

Cluster 5 included 175 genes with the majority of them downregulated in *P. pastoris *under hypoxic and oxygen-limited conditions. While the genes for *S. cerevisiae *were marginally induced when comparing hypoxic with aerobic conditions, the profile was rather unchanged for the other pairwise comparisons. This cluster was enriched with TCA cycle genes (*KGD2, SDH4, LSC1, LSC2, CIT1 *and *FUM1*) and genes involved in amino acid biosynthetic process (*LYS21, GLT1, ILV2, SER2, HIS6*, and *ARO7*). The downregulation of the TCA cycle in hypoxic *P. pastoris *chemostat cultivations on the transcriptome, proteome and metabolome levels was recently reported by our group [15]. While in *S. cerevisiae*, a similar repression of TCA cycle transcripts was observed for anaerobic glucose-limited chemostats [23,48,49], intermediate oxygen conditions with 2.8 % oxygen in the inlet gas induced a moderate upregulation of the transcripts for the main TCA cycle enzymes [31], which is in accordance with the results in this study. In this context, the similar regulation of the amino acid biosynthetic genes may be partly interrelated, since the TCA cycle provides biosynthetic precursors for amino acids including lysine (*LYS21*), glutamine (*GLT1*) and isoleucine (*ILV2*) [50].

A set of genes was associated with response to oxidative (*GRX3, DOT5, SRX1, CCP1*) and DNA damage stress (*MSH3, HAM1, YNK1*). Aerobically grown cells are always exposed to some degree of oxidative stress, and activate defence mechanisms in order to repair (or to prevent further) damage. It thus seems plausible that *P. pastoris *silences the oxidative stress defences in hypoxic and oxygen-limited conditions as compared to normoxia. Interestingly, the transcript levels of such stress related genes in hypoxically grown *S. cerevisiae *were slightly induced. It is very unlikely that a surplus of reducing equivalents in low oxygen environments allows for the generation of radicals. Krantz and co-workers [51] observed a similar trend in *S. cerevisiae *chemostats under anaerobic osmo-stress conditions, and assumed a co-regulation with general stress response genes (like *CCT1*), however, without any evident requirement.

Three glycogen biosynthetic genes (*GLG1, GSY2*, and *GAC1*) were also present in cluster 5. Glycogen, a highly abundant reserve compound in yeasts and composed of glucose, is generally considered to provide both carbon and energy during starvation periods (extensively reviewed in [52]). *P. pastoris' *glycogen content was shown previously to be higher in aerobic conditions than in conditions of low oxygen provision [27], which coincided with the observed downregulation of the related genes under oxygen limitation. It seems plausible that *P. pastoris *cells do not synthesize glycogen in glucose-limited hypoxic cultures, but rather mobilize stored glycogen for energy purposes and the conservation of a high glycolytic flux. The unchanged/slightly induced transcripts of glycogen-synthetic genes in *S. cerevisiae *during hypoxia, in contrast, remain unclear, but could be related to the involvement of glycogen accumulation during spore formation in diploid cells [53]. Evidence for increased spore formation in hypoxically grown *S. cerevisiae *is given by the induced genes associated with ascospore formation in cluster 9.

Another functional category that is enriched in cluster 5 consists of five transcription factors controlling various cellular functions, like mating (*HMRA1, STE12*), ethanol, glycerol and fatty acid utilization and peroxisomal gene transcription (*ADR1*), nucleotide excision repair (*TFB3*) and phospholipid biosynthesis (*OPI1*). A number of known targets of these transcription factors were also present in the downregulated clusters.

Cluster 7 contained genes with a low level of expression in *S. cerevisiae *and with induced transcripts in *P. pastoris *in hypoxic and oxygen-limited conditions, as compared to normoxic conditions. This cluster was considerably enriched with genes of the ergosterol pathway (*ERG3, ERG4, ERG5, ERG6, ERG24, ERG25, ERG26 *and *ERG27*). As discussed before (see Omics expression viewer Section), there are some differences on ergosterol regulation in *S. cerevisiae *under anaerobic or oxygen-limited conditions [31]. Additionally, the usual supplement of ergosterol to anaerobically grown *S. cerevisiae *hampers a direct comparison with our data, since we did not add ergosterol in any oxygen condition. The results of this comparative analysis, however, clearly indicate remarkable differences in the regulation of sterol pathways in the two yeasts in a reduced oxygen environment.

Two trehalose metabolic genes (*TPS2 *and *ATH1*) were also found in this cluster. Tps2p catalyzes the synthesis of the reserve compound trehalose, which is also known as stress protecting agent (reviewed in [54]) that has an extraordinary capacity of stabilizing proteins and preventing aggregation of denatured proteins [55]. Chen et al. [56] observed that trehalose accumulation in *Drosophila *protected the cells from hypoxic injury. However, in a recent study on the macromolecular and elemental composition of *P. pastoris *grown under the exactly same experimental conditions [27], no trehalose accumulation could be detected when varying the oxygenation degree or the strain genetic background. Since *ATH1 *is involved in the catabolism of trehalose, it appears that the trehalose pool might have a higher turnover in hypoxic and oxygen-limited *P. pastoris *cultivations. In agreement with the transcriptional profiles, instead, the trehalose content of *S. cerevisiae *decreased when reducing oxygen supply (data not shown), as similarly reported elsewhere [23].

The regulation of genes encoding enzymes capable of xylose reduction (*GCY1 *and *YJR096W*) appears to be characteristic for *P. pastoris*. Xylose reduction is most probably involved in the formation of arabitol in *P. pastoris *hypoxic fermentations. Another gene of special interest in this cluster was related to protein secretion (*NCE102*) since it is involved in non-classical protein export [57]. Similarly, we recently identified *NCE103*, encoding a substrate for the non-classical protein export pathway, to be hypoxically induced in *P. pastoris*, with a significantly stronger upregulation in the producing strain [15].

Cluster 9 (26 members) is primarily characterized by strongly induced mRNA levels in *S. cerevisiae *and unchanged or slightly downregulated genes in *P. pastoris *hypoxic and oxygen-limited conditions, as compared to fully aerobic conditions. Some exceptions showed a uniform upregulated pattern for all pairwise comparisons (*FSH1, GSA2, PHO84 *and *WSC4*) or a strong upregulation in the *P. pastoris *control strain (*YCT1*). *OPT2*, an oligopeptide transporter with a role in vacuole assembly, and *CDA2*, ascospore wall formation, showed a stronger induction in the *S. cerevisiae *producing strain. The cluster was predominantly composed of ascospore formation genes (*CDA2, GAS2, SPO75, SPR1, SPR3 *and *SPS2*) and transmembrane transporter genes (*DAL4, DAL5, YCT1, PHO84, FET4 *and *OPT2*). As already discussed before, spore formation mainly derives from diploid cells, which is a very uncommon state in the life cycle of *P. pastoris*. Spore formation, however, is also known to be a strictly aerobic process because it only takes place in the presence of a poor carbon source and in cells starved of nitrogen ([58], and reviewed in [59]). Therefore, the upregulation of some spore formation associated genes might be related to other adaptive processes towards low oxygen availability or rather alert the cells to prepare them for more environmental extremes.

*DAL4 *and *DAL5 *are transporters of allantoin - an intermediate of adenine and guanine degradation that can serve as sole nitrogen source. The induction of these genes in *S. cerevisiae *could be a hint to a higher need of nitrogen under hypoxic conditions as compared to *P. pastoris*. Also, the upregulation of some transmembrane transporters may indicate some extra demand in *S. cerevisiae *for important building blocks like metals, amino acids or oligopeptides.

Cluster 10 was mainly composed of glycolytic genes (*CDC19, ENO1, EXG1, GLK1, GPM1, PFK1, PFK2, PGK1*, and *TDH3*) and stress-related genes (*UGA2, YDL124W, HSP12, TSA1 *and *NCE103*). Furthermore, it included two important protein folding-associated genes (*ERO1 *and *HAC1*) and three ergosterol genes (*ERG1, ERG11 *and *ERG28*). The pattern in this cluster was predominantly characterized by a strong induction in HvN and LvN in *P. pastoris *strains. The transcriptional induction of glycolysis and ergosterol synthesis in *P. pastoris *under low oxygen conditions has already been discussed in the "Omics expression viewer" section. Among the stress related genes, *NCE103 *is known to have a protective role in the presence of H_2_O_2_, but also a participation in a non conventional protein export, as observed for *NCE102*, has been proposed [57]. *UGA2*, a succinate semialdehyde dehydrogenase with a function in gamma-aminobutyric acid (GABA) degradation, was also reported to be induced by H_2_O_2 _thus increasing oxidative stress tolerance [60]. However, it is very unlikely that hypoxia triggered such antioxidant responses, since no respiratory activity could be detected in the case of *P. pastoris*. We rather propose some other function of their gene products, like the involvement of Nce103p in protein secretion as previously mentioned, or the role of Uga2p in degrading the non-protein amino acid GABA for nitrogen utilization. A further "moonlighting" protein with multiple functions is the gene product of *TSA1*, which plays a role as antioxidant under normal conditions [61], but self-associates to form a chaperone complex as a consequence of environmental perturbations. In its chaperone state it targets unfolded proteins in order to prevent their aggregation [62]. *HSP12*, another chaperone-encoding gene that was induced by hypoxia in *P. pastoris*, confers increased stability to membranes in the presence of ethanol.

Many chaperones are helper factors throughout the protein folding process and assure the release of only correctly folded polypeptides from the endoplasmic reticulum (ER). Environmental stresses or similar events can easily provoke an overload of the ER folding machine, resulting in the aggregation of unprocessed proteins and consequently in the onset of the unfolded protein response (UPR) (reviewed in [63]). Transcripts of the transcription factor activating this cellular mechanism, Hac1p, were also significantly induced in *P. pastoris *as compared to *S. cerevisiae*. Notably, unlike in *P. pastoris, S. cerevisiae HAC1 *is not regulated at the transcriptional level upon UPR, but only by splicing of the transcript (*HAC1s*). This means that comparing UPR induction in both yeasts can be done only on a basis of UPR target genes like *KAR2, PDI1 *and *ERO1*. Since none of these genes was upregulated in *S. cerevisiae*, we could exclude any UPR activity in this yeast, which would explain also why other chaperones like *TSA1 *and *HSP12 *were unchanged as well. In good consistency to the overall results of this study, *HAC1 *regulation could also reasonably be linked to changes in the lipid balance, since UPR was also suggested to be activated upon lipid deprivation in order to coordinate membrane synthesis, with Hac1 as important regulator [64]. Since we also observed a drastic difference between the yeasts in the regulation of ergosterol biosynthesis, alterations in membrane fluidity could have provoked the upregulation of *P. pastoris HAC1*.

In good agreement with these results, also *ERO1*, required for oxidative protein folding in the endoplasmic reticulum, was activated upon hypoxia in *P. pastoris*. Together with *PDI1*, another key player during UPR, it catalyzes the transfer of oxidizing equivalents to folding proteins [65]. It is likely that the differences in the regulation of the protein folding machinery are reflected in the different protein product yields in *P. pastoris *and *S. cerevisiae*, since a number of studies have reported a stimulatory effect of the overexpression of UPR related genes (e.g. *PDI1, HAC1s *and *ERO1*) on recombinant protein secretion [9].

It is apparent from this work that there are a number of pathways that are not overlapping in their response to oxygen availability in these two yeasts, thus reflecting important differences in the regulation of their metabolisms.

## Conclusions

The effect of three different conditions of oxygen provision on the transcriptome of *P. pastoris *and *S. cerevisiae *revealed a very distinct remodelling of the genomic expression program, particularly in the case of hypoxia. On one side, such observed differences point to a different importance of transcriptional regulation mechanisms in respect to metabolic remodelling following adaptation to reduced oxygen availability, which probably reflect a divergence in the evolution of control mechanisms preferred by the two yeasts. This might be related to different strategies for cell survival in the competition with other microorganisms: For example, a preference towards post-transcriptional or post-translational regulation mechanisms, as inferred for *S. cerevisiae*, gives the possibility of a faster adaptation to changing conditions and, therefore, more versatility. On the other side, clear differences between the two yeasts were detected at the transcriptome level for several cellular processes related with protein secretion, namely the ergosterol biosynthesis pathway, the central carbon metabolism and the stress and unfolded protein response. Such physiological differences underlying the distinct impact of hypoxia on protein secretion in the two yeasts give a strong indication on those pathways whose regulation/manipulation might lead to improved phenotypes with respect to heterologous protein production under well defined environmental conditions.

Overall, the results of this study indicate that combination of systems biology approaches and biological systems diversity is a useful tool to gain novel insights into the physiology of biotechnologically important microorganisms, from which new strategies for rational optimization of strains and cultivation conditions could be derived.

## Methods

### Strains and chemostat cultivations

#### Pichia pastoris

The construction of the *P. pastoris *strains used in this study as well as a detailed description of the chemostat cultivations and sampling are reported elsewhere [15]. In brief, a recombinant *P. pastoris *X-33 (wild type phenotype) derived strain and its corresponding empty-vector control strain were cultivated in a glucose-limited chemostat at a dilution rate of 0.1 h^-1^. The recombinant strain secreted the light and heavy chain of a human monoclonal antibody Fab (3H6) fragment under the constitutive GAP promoter. Cultivations were performed at 3 different oxygen concentrations in the inlet gas stream, yielding normoxic (21 % O_2_), oxygen-limited (11 % O_2_) and hypoxic (8 % O_2_) conditions. Triplicate samples for the DNA microarray experiments were taken at steady state conditions for each oxygen set-point.

#### Saccharomyces cerevisiae

The coding sequences for the 3H6 Fab heavy and light chain were both integrated on a pYX integrative expression vector under control of the constitutive *S. cerevisiae TPI1 *promoter. The *S. cerevisiae *α-factor leader sequence was used to target the protein into the supernatant, and a polyA sequence served as terminator. The expression vectors were integrated into the genome of *S. cerevisiae *CEN.PK strain with *HIS3 *and *URA3 *as selection markers. The control strain was transformed with the empty pYX vectors.

Chemostat cultivations were performed in a bench-top bioreactor (Biostat B, Braun Biotech International GmbH) at a working volume of 1.25 L. After a batch period of approximately 24 hours, chemostat cultivation was initiated at a dilution rate of D = 0.1 h^-1^. The parameters were set to 1000 rpm and 26°C, and the pH was controlled at 5.0. The total gas flow was kept constant for all experiments at 1 vvm (volume gas per volume medium and minute). While normoxic cultivations were run with pure air in the inlet gas, the oxygen concentration was reduced to 5 % and 2 % for the oxygen-limited and hypoxic conditions, respectively, by partially replacing the air with nitrogen. Samples were taken from three independent experiments after reaching steady state conditions, i.e. after at least 5 residence times τ.

### Microarray experiments

The *P. pastoris *specific DNA microarrays used in this study were produced on the Agilent platform (Agilent Technologies) and established by Graf et al. [12]. The transcriptome analysis for *S. cerevisiae *was carried out with Agilent Yeast Gene Expression Microarrays, 4 × 44K. For both yeast species, RNA extraction, cDNA synthesis and labeling, as well as the microarray hybridizations and data analysis were performed as reported in previous studies [12,14]. All samples were labeled in a dye-swap manner and hybridized against a reference cDNA, which was generated from a pool of cells (either *P. pastoris *or *S. cerevisiae*) grown under different culture conditions. Microarray data are available in the ArrayExpress database http://www.ebi.ac.uk/arrayexpress under accession number E-MEXP-2742 for *P. pastoris *and E-MEXP-3134 for *S. cerevisiae*.

### Data analysis

The common gene list between *P. pastoris *and *S. cerevisiae *contains orthologs of the two species. Orthology was determined using a reciprocal best hit strategy based on protein BLASTs of *P. pastoris *proteins against *S. cerevisiae *proteins and vice versa. The protein BLAST was performed with an E-value cut-off < 10^-5 ^and the BLAST reports were filtered for hits that had at least one high scoring segment > 50 amino acids with a similarity > 40 %. Of the resulting list, 388 gene IDs had to be excluded since they were either missing on the *S. cerevisiae *or the *P. pastoris *microarray. Additional file 1 contains the final list of orthologs (2891 gene IDs) with an indication of the number of *S. cerevisiae *proteins for which the respective *P. pastoris *protein was the best hit. A complete account of the annotation of *P. pastoris *can be found in De Schutter et al. [10] and Mattanovich et al. [11].

Principal Component Analysis (PCA) and Fisher's exact test for identification of significantly regulated gene groups based on gene ontology classes (GO groups) were carried our using the R platform http://www.r-project.org. The selection of GO groups was based on the GOSlim terms with some very large classes being resolved in more detail. The absolute (normalized) expression values of each yeast species were applied in both analyses.

Fold change expression data were subjected to the 'Expression Omics Viewer' tool from the SGD Saccharomyces genome database http://www.yeastgenome.org for visualization of the metabolic gene regulation when comparing hypoxic with normoxic conditions. A three color display with a specified threshold was applied, with red for data values that exceed the threshold of 0.59 (log2 fold change, equivalent to a fold change of 1.5), yellow for data values less than the inverse of that threshold, and blue for values in between.

Data clustering and visualization were performed with the open source tool Expression Profiler from EBI http://www.ebi.ac.uk/expressionprofiler. A cluster comparison was run between hierarchical clustering (correlation-based distance, average linkage) and k-means clustering (Euclidean distance, k = 10). Main clusters were manually analyzed for the most relevant discriminatory GO groups and genes for each organism, strain or oxygen comparison.

## Authors' contributions

KB performed the *P. pastoris *bioreactor cultivations, microarray experiments, data analysis and interpretation of the results, and drafted the manuscript. GF performed the *S. cerevisiae *fermentations. LD carried out the *S. cerevisiae *microarray experiments, analyzed and interpreted the results and helped in drafting the manuscript. ABG participated in the design and bioinformatics analysis of the microarrays. MD assisted in the design and performance of the microarray experiments. DP, PB, PF and DM participated in the conceptual and experimental design of this study, interpretation of results and revision of the manuscript. All authors read and approved the final manuscript.

## Supplementary Material

Additional file 1 **List of orthologues**. List of the orthologues (2891 gene IDs) between *S. cerevisiae *and *P. pastoris *used for the direct comparison of the results of the transcriptional analysis. Orthology was determined using a reciprocal best hit strategy based on protein BLASTs (E-value cut-off < 10^-5 ^and filtering for at least one high scoring segment > 50 amino acids with a similarity > 40%). 388 gene IDs passing the filters were excluded since they were missing either on the *S. cerevisiae *or on the *P. pastoris *microarrays.
Column A, *P. pastoris *gene IDs used in microarrays; column B, *S. cerevisiae *systematic names; column C, *S. cerevisiae *short gene names (corresponding to the protein names); column D, number of *S. cerevisiae *genes for which the *P. pastoris *protein was the best hit. Click here for file

Additional file 2 **MetaCyc Data *Saccharomyces cerevisiae***. Regulated *S. cerevisiae *pathways in hypoxia *vs*. normoxia. Individual *S. cerevisiae *(recombinant strain) pathways that were transcriptionally regulated (*i e*. exceeding the log2 FC threshold of 0.59) in the comparison hypoxic *vs*. normoxic conditions, as resulting from the MetaCyc analysis presented in Figure 3 http://pathway.yeastgenome.org. Pathway numbers in the first column are referred to Figure 3. Pathway diagrams show all the intermediates of each pathways; reaction lines and the corresponding genes are colour-coded (three colour bins) according to the fold change threshold: red for upregulated, yellow for downregulated and blue for unregulated; log2 FC for each gene are also shown in colour. Last column contains the extended enzyme names corresponding to each gene of the pathway. Click here for file

Additional file 3 **MetaCyc data *Pichia pastoris***. Regulated *P. pastoris *pathways in hypoxia *vs*. normoxia. Individual *P. pastoris *(recombinant strain) pathways that were transcriptionally regulated (*i e*. exceeding the log2 FC threshold of 0.59) in the comparison hypoxic *vs*. normoxic conditions, as resulting from the MetaCyc analysis presented in Figure 3 http://pathway.yeastgenome.org. Pathway numbers in the first column are referred to Figure 3. Pathway diagrams show all the intermediates of each pathways; reaction lines and the corresponding genes are colour-coded (three colour bins) according to the fold change threshold: red for upregulated, yellow for downregulated and blue for unregulated; log2 FC for each gene are also shown in colour. Last column contains the extended enzyme names corresponding to each gene of the pathway. Click here for file

Additional file 4 **Metabolic pathways**. List of metabolic pathways that correspond to the numbers indicated on the cellular overview chart depicted in Figure 3. Click here for file

Additional file 5 **Gene list of single clusters**. Lists of genes corresponding to each of the cluster resulting from the k-means clustering (k = 10) depicted in Figure 4B (obtained by Expression Profiler analysis; http://www.ebi.ac.uk/expressionprofiler). Each column contains a single cluster. Column names indicate cluster number and, in brackets, the total number of genes present in each cluster. Click here for file
