# Supplementary material for: The impact of oxygen on the transcriptome of recombinant S. cerevisiae and P. pastoris - a comparative analysis
Source: BMC Genomics. 2011 May 9;12:218. doi: 10.1186/1471-2164-12-218 (PMC3116504; doi:10.1186/1471-2164-12-218)
Supplement: Additional file 2 — MetaCyc Data Saccharomyces cerevisiae. Regulated S. cerevisiae pathways in hypoxia vs. normoxia. Individual S. cerevisiae (recombinant strain) pathways that were transcriptionally regulated (i e. exceeding the log2 FC threshold of 0.59) in the comparison hypoxic vs. normoxic conditions, as resulting from the MetaCyc analysis presented in Figure 3 http://pathway.yeastgenome.org. Pathway numbers in the first column are referred to Figure 3. Pathway diagrams show all the intermediates of each pathways; reaction lines and the corresponding genes are colour-coded (three colour bins) according to the fold change threshold: red for upregulated, yellow for downregulated and blue for unregulated; log2 FC for each gene are also shown in colour. Last column contains the extended enzyme names corresponding to each gene of the pathway. [file 1471-2164-12-218-S2.DOC]

**Additional file 2 – Regulated *S.cerevisiae* pathways in hypoxia vs. normoxia**

Individual *S. cerevisiae* (Fab-producing strain) pathways transcriptionally regulated (*i e.* exceeding log2 FC threshold of 0.59) in the comparison hypoxic vs. normoxic conditions, as resulting from MetaCyc analysis (see Figure 3). Pathway numbers are referred to Figure 3 legend.

| **N^o^** | **Pathway** | **Pathway Diagram** | **Enzymes, Genes** |
| --- | --- | --- | --- |
| 8 | [lysine biosynthesis](http://pathway.yeastgenome.org/YEAST/NEW-IMAGE?type=PATHWAY&object=YEAST-ARG-SYN-PWY) | 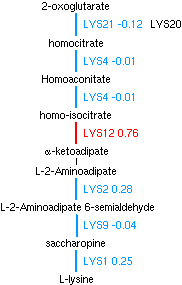 | | [homocitrate synthase](http://pathway.yeastgenome.org/YEAST/NEW-IMAGE?type=ENZYME&object=YDL182W-MONOMER) | [LYS20](http://db.yeastgenome.org/cgi-bin/locus.pl?locus=S000002341) | | --- | --- | | [homocitrate synthase](http://pathway.yeastgenome.org/YEAST/NEW-IMAGE?type=ENZYME&object=YDL131W-MONOMER) | [LYS21](http://db.yeastgenome.org/cgi-bin/locus.pl?locus=S000002289) | | [homoaconitase](http://pathway.yeastgenome.org/YEAST/NEW-IMAGE?type=ENZYME&object=YDR234W-MONOMER) | [LYS4](http://db.yeastgenome.org/cgi-bin/locus.pl?locus=S000002642) | | [homo-isocitrate dehydrogenase](http://pathway.yeastgenome.org/YEAST/NEW-IMAGE?type=ENZYME&object=YIL094C-MONOMER) | [LYS12](http://db.yeastgenome.org/cgi-bin/locus.pl?locus=S000001356) | | [alpha aminoadipate reductase](http://pathway.yeastgenome.org/YEAST/NEW-IMAGE?type=ENZYME&object=YBR115C-MONOMER) | [LYS2](http://db.yeastgenome.org/cgi-bin/locus.pl?locus=S000000319) | | [saccharopine dehydrogenase (NADP+, L-glutamate-forming)](http://pathway.yeastgenome.org/YEAST/NEW-IMAGE?type=ENZYME&object=MONOMER3O-363) | [LYS9](http://db.yeastgenome.org/cgi-bin/locus.pl?locus=S000005333) | | [saccharopine dehydrogenase (NAD+, L-lysine-forming)](http://pathway.yeastgenome.org/YEAST/NEW-IMAGE?type=ENZYME&object=YIR034C-MONOMER) | [LYS1](http://db.yeastgenome.org/cgi-bin/locus.pl?locus=S000001473) | |
| 8 | [arginine biosynthesis](http://pathway.yeastgenome.org/YEAST/NEW-IMAGE?type=PATHWAY&object=YEAST-ARG-SYN-PWY) | 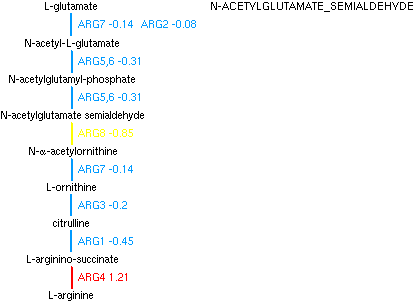 | | [acetylglutamate synthase](http://pathway.yeastgenome.org/YEAST/NEW-IMAGE?type=ENZYME&object=YJL071W-MONOMER) | [ARG2](http://db.yeastgenome.org/cgi-bin/locus.pl?locus=S000003607) | | --- | --- | | [acetylglutamate kinase / N-acetyl-gamma-glutamyl-phosphate reductase](http://pathway.yeastgenome.org/YEAST/NEW-IMAGE?type=ENZYME&object=YER069W-MONOMER) | [ARG5,6](http://db.yeastgenome.org/cgi-bin/locus.pl?locus=S000000871) | | [acetylornithine aminotransferase](http://pathway.yeastgenome.org/YEAST/NEW-IMAGE?type=ENZYME&object=YOL140W-MONOMER) | [ARG8](http://db.yeastgenome.org/cgi-bin/locus.pl?locus=S000005500) | | [acetylornithine acetyltransferase](http://pathway.yeastgenome.org/YEAST/NEW-IMAGE?type=ENZYME&object=YMR062C-MONOMER) | [ARG7](http://db.yeastgenome.org/cgi-bin/locus.pl?locus=S000004666) | | [ornithine carbamoyltransferase](http://pathway.yeastgenome.org/YEAST/NEW-IMAGE?type=ENZYME&object=YJL088W-MONOMER) | [ARG3](http://db.yeastgenome.org/cgi-bin/locus.pl?locus=S000003624) | | [arginosuccinate synthetase](http://pathway.yeastgenome.org/YEAST/NEW-IMAGE?type=ENZYME&object=YOL058W-MONOMER) | [ARG1](http://db.yeastgenome.org/cgi-bin/locus.pl?locus=S000005419) | | [argininosuccinate lyase](http://pathway.yeastgenome.org/YEAST/NEW-IMAGE?type=ENZYME&object=YHR018C-MONOMER) | [ARG4](http://db.yeastgenome.org/cgi-bin/locus.pl?locus=S000001060) | |
| 9 | [arginine degradation (anaerobic)](http://pathway.yeastgenome.org/YEAST/NEW-IMAGE?type=PATHWAY&object=ARG-PRO-PWY) | 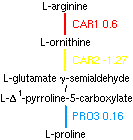 | | [arginase](http://pathway.yeastgenome.org/YEAST/NEW-IMAGE?type=ENZYME&object=YPL111W-MONOMER) | [CAR1](http://db.yeastgenome.org/cgi-bin/locus.pl?locus=S000006032) | | --- | --- | | [ornithine aminotransferase](http://pathway.yeastgenome.org/YEAST/NEW-IMAGE?type=ENZYME&object=YLR438W-MONOMER) | [CAR2](http://db.yeastgenome.org/cgi-bin/locus.pl?locus=S000004430) | | [delta 1-pyrroline-5-carboxylate reductase](http://pathway.yeastgenome.org/YEAST/NEW-IMAGE?type=ENZYME&object=YER023W-MONOMER) | [PRO3](http://db.yeastgenome.org/cgi-bin/locus.pl?locus=S000000825) | |
| 10 | [arginine biosynthesis](http://pathway.yeastgenome.org/YEAST/NEW-IMAGE?type=PATHWAY&object=ARGSYNBSUB-PWY) | 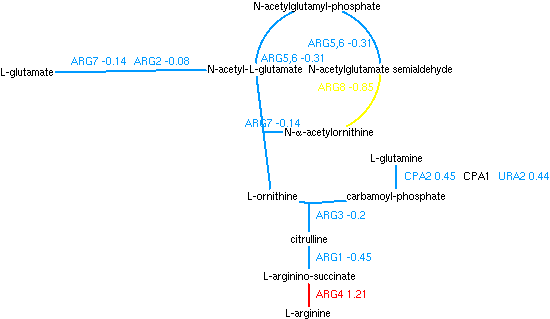 | | [acetylglutamate synthase](http://pathway.yeastgenome.org/YEAST/NEW-IMAGE?type=ENZYME&object=YJL071W-MONOMER) | [ARG2](http://db.yeastgenome.org/cgi-bin/locus.pl?locus=S000003607) | | --- | --- | | [acetylglutamate kinase / N-acetyl-gamma-glutamyl-phosphate reductase](http://pathway.yeastgenome.org/YEAST/NEW-IMAGE?type=ENZYME&object=YER069W-MONOMER) | [ARG5,6](http://db.yeastgenome.org/cgi-bin/locus.pl?locus=S000000871) | | [acetylornithine aminotransferase](http://pathway.yeastgenome.org/YEAST/NEW-IMAGE?type=ENZYME&object=YOL140W-MONOMER) | [ARG8](http://db.yeastgenome.org/cgi-bin/locus.pl?locus=S000005500) | | [acetylornithine acetyltransferase](http://pathway.yeastgenome.org/YEAST/NEW-IMAGE?type=ENZYME&object=YMR062C-MONOMER) | [ARG7](http://db.yeastgenome.org/cgi-bin/locus.pl?locus=S000004666) | | [carbamyl phosphate synthase / aspartate transcarbamylase](http://pathway.yeastgenome.org/YEAST/NEW-IMAGE?type=ENZYME&object=YJL130C-MONOMER) | [URA2](http://db.yeastgenome.org/cgi-bin/locus.pl?locus=S000003666) | | [carbamoyl phosphate synthetase](http://pathway.yeastgenome.org/YEAST/NEW-IMAGE?type=ENZYME&object=CPLX3O-887) | [CPA2](http://db.yeastgenome.org/cgi-bin/locus.pl?locus=S000003870) [CPA1](http://db.yeastgenome.org/cgi-bin/locus.pl?locus=S000005829) | | [ornithine carbamoyltransferase](http://pathway.yeastgenome.org/YEAST/NEW-IMAGE?type=ENZYME&object=YJL088W-MONOMER) | [ARG3](http://db.yeastgenome.org/cgi-bin/locus.pl?locus=S000003624) | | [arginosuccinate synthetase](http://pathway.yeastgenome.org/YEAST/NEW-IMAGE?type=ENZYME&object=YOL058W-MONOMER) | [ARG1](http://db.yeastgenome.org/cgi-bin/locus.pl?locus=S000005419) | | [argininosuccinate lyase](http://pathway.yeastgenome.org/YEAST/NEW-IMAGE?type=ENZYME&object=YHR018C-MONOMER) | [ARG4](http://db.yeastgenome.org/cgi-bin/locus.pl?locus=S000001060) | |
| 12 | [serine biosynthesis from 3-phosphoglycerate](http://pathway.yeastgenome.org/YEAST/NEW-IMAGE?type=PATHWAY&object=PWY3O-230) | 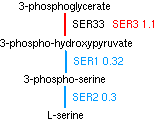 | | [3-phosphoglycerate dehydrogenase](http://pathway.yeastgenome.org/YEAST/NEW-IMAGE?type=ENZYME&object=YER081W-MONOMER) | [SER3](http://db.yeastgenome.org/cgi-bin/locus.pl?locus=S000000883) | | --- | --- | | [3-phosphoglycerate dehydrogenase](http://pathway.yeastgenome.org/YEAST/NEW-IMAGE?type=ENZYME&object=YIL074C-MONOMER) | [SER33](http://db.yeastgenome.org/cgi-bin/locus.pl?locus=S000001336) | | [phosphoserine transaminase](http://pathway.yeastgenome.org/YEAST/NEW-IMAGE?type=ENZYME&object=YOR184W-MONOMER) | [SER1](http://db.yeastgenome.org/cgi-bin/locus.pl?locus=S000005710) | | [phosphoserine phosphatase](http://pathway.yeastgenome.org/YEAST/NEW-IMAGE?type=ENZYME&object=YGR208W-MONOMER) | [SER2](http://db.yeastgenome.org/cgi-bin/locus.pl?locus=S000003440) | |
| 12 | [serine biosynthesis from glyoxylate](http://pathway.yeastgenome.org/YEAST/NEW-IMAGE?type=PATHWAY&object=PWY3O-230) | 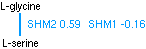 | | [Serine hydroxymethyltransferase, mitochondrial](http://pathway.yeastgenome.org/YEAST/NEW-IMAGE?type=ENZYME&object=YBR263W-MONOMER) | [SHM1](http://db.yeastgenome.org/cgi-bin/locus.pl?locus=S000000467) | | --- | --- | | [serine hydroxymethyltransferase](http://pathway.yeastgenome.org/YEAST/NEW-IMAGE?type=ENZYME&object=YLR058C-MONOMER) | [SHM2](http://db.yeastgenome.org/cgi-bin/locus.pl?locus=S000004048) | |
| 12 | [glycine biosynthesis from serine](http://pathway.yeastgenome.org/YEAST/NEW-IMAGE?type=PATHWAY&object=GLYSYN-SER-PWY) | 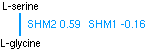 | | [Serine hydroxymethyltransferase, mitochondrial](http://pathway.yeastgenome.org/YEAST/NEW-IMAGE?type=ENZYME&object=YBR263W-MONOMER) | [SHM1](http://db.yeastgenome.org/cgi-bin/locus.pl?locus=S000000467) | | --- | --- | | [serine hydroxymethyltransferase](http://pathway.yeastgenome.org/YEAST/NEW-IMAGE?type=ENZYME&object=YLR058C-MONOMER) | [SHM2](http://db.yeastgenome.org/cgi-bin/locus.pl?locus=S000004048) | |
| 13 | [homocysteine and cysteine interconversion](http://pathway.yeastgenome.org/YEAST/NEW-IMAGE?type=PATHWAY&object=HOMOCYS-CYS-CONVERT) | 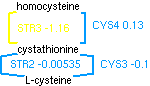 | | [cystathionine gamma-synthase](http://pathway.yeastgenome.org/YEAST/NEW-IMAGE?type=ENZYME&object=YJR130C-MONOMER) | [STR2](http://db.yeastgenome.org/cgi-bin/locus.pl?locus=S000003891) | | --- | --- | | [cystathionine beta-lyase](http://pathway.yeastgenome.org/YEAST/NEW-IMAGE?type=ENZYME&object=YGL184C-MONOMER) | [STR3](http://db.yeastgenome.org/cgi-bin/locus.pl?locus=S000003152) | | [cystathionine beta-synthase](http://pathway.yeastgenome.org/YEAST/NEW-IMAGE?type=ENZYME&object=YGR155W-MONOMER) | [CYS4](http://db.yeastgenome.org/cgi-bin/locus.pl?locus=S000003387) | | [cystathionine gamma-lyase](http://pathway.yeastgenome.org/YEAST/NEW-IMAGE?type=ENZYME&object=YAL012W-MONOMER) | [CYS3](http://db.yeastgenome.org/cgi-bin/locus.pl?locus=S000000010) | |
| 17 | [glutamate biosynthesis from ammonia](http://pathway.yeastgenome.org/YEAST/NEW-IMAGE?type=PATHWAY&object=GLUNH3-PWY) | 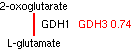 | | [NADP-dependent glutamate dehydrogenase](http://pathway.yeastgenome.org/YEAST/NEW-IMAGE?type=ENZYME&object=YAL062W-MONOMER) | [GDH3](http://db.yeastgenome.org/cgi-bin/locus.pl?locus=S000000058) | | --- | --- | | [NADP-dependent glutamate dehydrogenase](http://pathway.yeastgenome.org/YEAST/NEW-IMAGE?type=ENZYME&object=YOR375C-MONOMER) | [GDH1](http://db.yeastgenome.org/cgi-bin/locus.pl?locus=S000005902) | |
| 17 | [superpathway of glutamate biosynthesis](http://pathway.yeastgenome.org/YEAST/NEW-IMAGE?type=PATHWAY&object=GLUTSYN-PWY) | 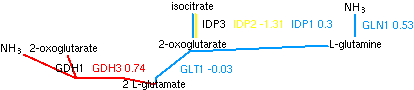 | | [NADP-dependent glutamate dehydrogenase](http://pathway.yeastgenome.org/YEAST/NEW-IMAGE?type=ENZYME&object=YAL062W-MONOMER) | [GDH3](http://db.yeastgenome.org/cgi-bin/locus.pl?locus=S000000058) | | --- | --- | | [NADP-dependent glutamate dehydrogenase](http://pathway.yeastgenome.org/YEAST/NEW-IMAGE?type=ENZYME&object=YOR375C-MONOMER) | [GDH1](http://db.yeastgenome.org/cgi-bin/locus.pl?locus=S000005902) | | [glutamine synthetase](http://pathway.yeastgenome.org/YEAST/NEW-IMAGE?type=ENZYME&object=YPR035W-MONOMER) | [GLN1](http://db.yeastgenome.org/cgi-bin/locus.pl?locus=S000006239) | | [NADP-dependent isocitrate dehydrogenase](http://pathway.yeastgenome.org/YEAST/NEW-IMAGE?type=ENZYME&object=YDL066W-MONOMER) | [IDP1](http://db.yeastgenome.org/cgi-bin/locus.pl?locus=S000002224) | | [NADP-dependent isocitrate dehydrogenase](http://pathway.yeastgenome.org/YEAST/NEW-IMAGE?type=ENZYME&object=YLR174W-MONOMER) | [IDP2](http://db.yeastgenome.org/cgi-bin/locus.pl?locus=S000004164) | | [NADP-dependent isocitrate dehydrogenase](http://pathway.yeastgenome.org/YEAST/NEW-IMAGE?type=ENZYME&object=YNL009W-MONOMER) | [IDP3](http://db.yeastgenome.org/cgi-bin/locus.pl?locus=S000004954) | | [glutamate synthase (NADH)](http://pathway.yeastgenome.org/YEAST/NEW-IMAGE?type=ENZYME&object=YDL171C-MONOMER) | [GLT1](http://db.yeastgenome.org/cgi-bin/locus.pl?locus=S000002330) | |
| 18 | [methionine salvage pathway](http://pathway.yeastgenome.org/YEAST/NEW-IMAGE?type=PATHWAY&object=PWY3O-64) | 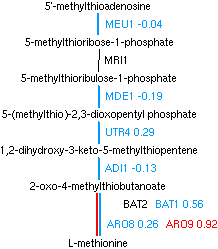 | | [5'-methylthioadenosine phosphorylase](http://pathway.yeastgenome.org/YEAST/NEW-IMAGE?type=ENZYME&object=MONOMER3O-157) | [MEU1](http://db.yeastgenome.org/cgi-bin/locus.pl?locus=S000004007) | | --- | --- | | [methylthioribose-1 P isomerase](http://pathway.yeastgenome.org/YEAST/NEW-IMAGE?type=ENZYME&object=CPLX3O-113) | [MRI1](http://db.yeastgenome.org/cgi-bin/locus.pl?locus=S000006322) | | [methylthioribulose-1-phosphate dehydratase](http://pathway.yeastgenome.org/YEAST/NEW-IMAGE?type=ENZYME&object=MONOMER3O-169) | [MDE1](http://db.yeastgenome.org/cgi-bin/locus.pl?locus=S000003785) | | [2,3-dioxomethiopentane-1-phosphate enolase/phosphatase](http://pathway.yeastgenome.org/YEAST/NEW-IMAGE?type=ENZYME&object=MONOMER3O-175) | [UTR4](http://db.yeastgenome.org/cgi-bin/locus.pl?locus=S000000764) | | [acireductone dioxygenase](http://pathway.yeastgenome.org/YEAST/NEW-IMAGE?type=ENZYME&object=MONOMER3O-186) | [ADI1](http://db.yeastgenome.org/cgi-bin/locus.pl?locus=S000004611) | | [aromatic amino acid aminotransferase II](http://pathway.yeastgenome.org/YEAST/NEW-IMAGE?type=ENZYME&object=YHR137W-MONOMER) | [ARO9](http://db.yeastgenome.org/cgi-bin/locus.pl?locus=S000001179) | | [aromatic amino acid aminotransferase I](http://pathway.yeastgenome.org/YEAST/NEW-IMAGE?type=ENZYME&object=YGL202W-MONOMER) | [ARO8](http://db.yeastgenome.org/cgi-bin/locus.pl?locus=S000003170) | | [branched-chain amino acid aminotransferase](http://pathway.yeastgenome.org/YEAST/NEW-IMAGE?type=ENZYME&object=YHR208W-MONOMER) | [BAT1](http://db.yeastgenome.org/cgi-bin/locus.pl?locus=S000001251) | | [branched-chain amino acid transaminase](http://pathway.yeastgenome.org/YEAST/NEW-IMAGE?type=ENZYME&object=YJR148W-MONOMER) | [BAT2](http://db.yeastgenome.org/cgi-bin/locus.pl?locus=S000003909) | |
| 20 | [ergosterol biosynthesis](http://pathway.yeastgenome.org/YEAST/NEW-IMAGE?type=PATHWAY&object=ERGOSTEROL-SYN-PWY) | 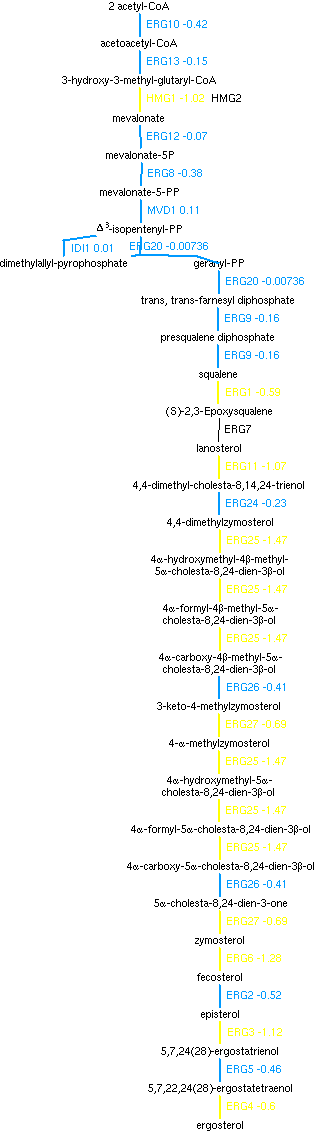  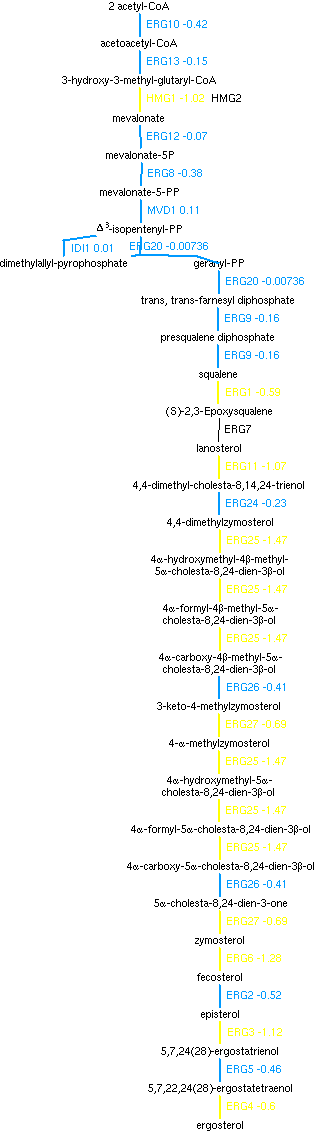 | | [acetoacetyl CoA thiolase](http://pathway.yeastgenome.org/YEAST/NEW-IMAGE?type=ENZYME&object=YPL028W-MONOMER) | [ERG10](http://db.yeastgenome.org/cgi-bin/locus.pl?locus=S000005949) | | --- | --- | | [3-hydroxy-3-methylglutaryl coenzyme A synthase](http://pathway.yeastgenome.org/YEAST/NEW-IMAGE?type=ENZYME&object=YML126C-MONOMER) | [ERG13](http://db.yeastgenome.org/cgi-bin/locus.pl?locus=S000004595) | | [3-hydroxy-3-methylglutaryl-coenzyme A (HMG-CoA)](http://pathway.yeastgenome.org/YEAST/NEW-IMAGE?type=ENZYME&object=YLR450W-MONOMER) | [HMG2](http://db.yeastgenome.org/cgi-bin/locus.pl?locus=S000004442) | | [3-hydroxy-3-methylglutaryl-coenzyme A (HMG-CoA)](http://pathway.yeastgenome.org/YEAST/NEW-IMAGE?type=ENZYME&object=YML075C-MONOMER) | [HMG1](http://db.yeastgenome.org/cgi-bin/locus.pl?locus=S000004540) | | [mevalonate kinase](http://pathway.yeastgenome.org/YEAST/NEW-IMAGE?type=ENZYME&object=YMR208W-MONOMER) | [ERG12](http://db.yeastgenome.org/cgi-bin/locus.pl?locus=S000004821) | | [phosphomevalonate kinase](http://pathway.yeastgenome.org/YEAST/NEW-IMAGE?type=ENZYME&object=YMR220W-MONOMER) | [ERG8](http://db.yeastgenome.org/cgi-bin/locus.pl?locus=S000004833) | | [mevalonate pyrophosphate decarboxylase](http://pathway.yeastgenome.org/YEAST/NEW-IMAGE?type=ENZYME&object=YNR043W-MONOMER) | [MVD1](http://db.yeastgenome.org/cgi-bin/locus.pl?locus=S000005326) | | [isopentenyl diphosphate:dimethylallyl diphosphate isomerase](http://pathway.yeastgenome.org/YEAST/NEW-IMAGE?type=ENZYME&object=YPL117C-MONOMER) | [IDI1](http://db.yeastgenome.org/cgi-bin/locus.pl?locus=S000006038) | | [farnesyl diphosphate synthetase](http://pathway.yeastgenome.org/YEAST/NEW-IMAGE?type=ENZYME&object=YJL167W-MONOMER) | [ERG20](http://db.yeastgenome.org/cgi-bin/locus.pl?locus=S000003703) | | [squalene synthetase](http://pathway.yeastgenome.org/YEAST/NEW-IMAGE?type=ENZYME&object=YHR190W-MONOMER) | [ERG9](http://db.yeastgenome.org/cgi-bin/locus.pl?locus=S000001233) | | [squalene monooxygenase](http://pathway.yeastgenome.org/YEAST/NEW-IMAGE?type=ENZYME&object=YGR175C-MONOMER) | [ERG1](http://db.yeastgenome.org/cgi-bin/locus.pl?locus=S000003407) | | [2,3-oxidosqualene-lanosterol cyclase](http://pathway.yeastgenome.org/YEAST/NEW-IMAGE?type=ENZYME&object=YHR072W-MONOMER) | [ERG7](http://db.yeastgenome.org/cgi-bin/locus.pl?locus=S000001114) | | [cytochrome P450 lanosterol 14a-demethylase](http://pathway.yeastgenome.org/YEAST/NEW-IMAGE?type=ENZYME&object=YHR007C-MONOMER) | [ERG11](http://db.yeastgenome.org/cgi-bin/locus.pl?locus=S000001049) | | [C-14 sterol reductase](http://pathway.yeastgenome.org/YEAST/NEW-IMAGE?type=ENZYME&object=YNL280C-MONOMER) | [ERG24](http://db.yeastgenome.org/cgi-bin/locus.pl?locus=S000005224) | | [C-4 sterol methyl oxidase](http://pathway.yeastgenome.org/YEAST/NEW-IMAGE?type=ENZYME&object=YGR060W-MONOMER) | [ERG25](http://db.yeastgenome.org/cgi-bin/locus.pl?locus=S000003292) | | [C-3 sterol dehydrogenase](http://pathway.yeastgenome.org/YEAST/NEW-IMAGE?type=ENZYME&object=YGL001C-MONOMER) | [ERG26](http://db.yeastgenome.org/cgi-bin/locus.pl?locus=S000002969) | | [3-keto sterol reductase](http://pathway.yeastgenome.org/YEAST/NEW-IMAGE?type=ENZYME&object=YLR100W-MONOMER) | [ERG27](http://db.yeastgenome.org/cgi-bin/locus.pl?locus=S000004090) | | [SAM:C-24 sterol methyltransferase](http://pathway.yeastgenome.org/YEAST/NEW-IMAGE?type=ENZYME&object=MONOMER3O-188) | [ERG6](http://db.yeastgenome.org/cgi-bin/locus.pl?locus=S000004467) | | [C-8 sterol isomerase](http://pathway.yeastgenome.org/YEAST/NEW-IMAGE?type=ENZYME&object=YMR202W-MONOMER) | [ERG2](http://db.yeastgenome.org/cgi-bin/locus.pl?locus=S000004815) | | [C-5 sterol desaturase](http://pathway.yeastgenome.org/YEAST/NEW-IMAGE?type=ENZYME&object=YLR056W-MONOMER) | [ERG3](http://db.yeastgenome.org/cgi-bin/locus.pl?locus=S000004046) | | [C-22 sterol desaturase](http://pathway.yeastgenome.org/YEAST/NEW-IMAGE?type=ENZYME&object=MONOMER3O-232) | [ERG5](http://db.yeastgenome.org/cgi-bin/locus.pl?locus=S000004617) | | [C-24 sterol reductase](http://pathway.yeastgenome.org/YEAST/NEW-IMAGE?type=ENZYME&object=YGL012W-MONOMER) | [ERG4](http://db.yeastgenome.org/cgi-bin/locus.pl?locus=S000002980) | |
| 20 | [mevalonate pathway](http://pathway.yeastgenome.org/YEAST/NEW-IMAGE?type=PATHWAY&object=IPPSYN-PWY) | 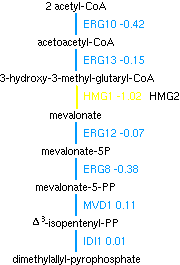 | | [acetoacetyl CoA thiolase](http://pathway.yeastgenome.org/YEAST/NEW-IMAGE?type=ENZYME&object=YPL028W-MONOMER) | [ERG10](http://db.yeastgenome.org/cgi-bin/locus.pl?locus=S000005949) | | --- | --- | | [3-hydroxy-3-methylglutaryl coenzyme A synthase](http://pathway.yeastgenome.org/YEAST/NEW-IMAGE?type=ENZYME&object=YML126C-MONOMER) | [ERG13](http://db.yeastgenome.org/cgi-bin/locus.pl?locus=S000004595) | | [3-hydroxy-3-methylglutaryl-coenzyme A (HMG-CoA)](http://pathway.yeastgenome.org/YEAST/NEW-IMAGE?type=ENZYME&object=YLR450W-MONOMER) | [HMG2](http://db.yeastgenome.org/cgi-bin/locus.pl?locus=S000004442) | | [3-hydroxy-3-methylglutaryl-coenzyme A (HMG-CoA)](http://pathway.yeastgenome.org/YEAST/NEW-IMAGE?type=ENZYME&object=YML075C-MONOMER) | [HMG1](http://db.yeastgenome.org/cgi-bin/locus.pl?locus=S000004540) | | [mevalonate kinase](http://pathway.yeastgenome.org/YEAST/NEW-IMAGE?type=ENZYME&object=YMR208W-MONOMER) | [ERG12](http://db.yeastgenome.org/cgi-bin/locus.pl?locus=S000004821) | | [phosphomevalonate kinase](http://pathway.yeastgenome.org/YEAST/NEW-IMAGE?type=ENZYME&object=YMR220W-MONOMER) | [ERG8](http://db.yeastgenome.org/cgi-bin/locus.pl?locus=S000004833) | | [mevalonate pyrophosphate decarboxylase](http://pathway.yeastgenome.org/YEAST/NEW-IMAGE?type=ENZYME&object=YNR043W-MONOMER) | [MVD1](http://db.yeastgenome.org/cgi-bin/locus.pl?locus=S000005326) | | [isopentenyl diphosphate:dimethylallyl diphosphate isomerase](http://pathway.yeastgenome.org/YEAST/NEW-IMAGE?type=ENZYME&object=YPL117C-MONOMER) | [IDI1](http://db.yeastgenome.org/cgi-bin/locus.pl?locus=S000006038) | |
| 30 | [phospholipid biosynthesis](http://pathway.yeastgenome.org/YEAST/NEW-IMAGE?type=PATHWAY&object=PHOSLIPSYN2-PWY) | 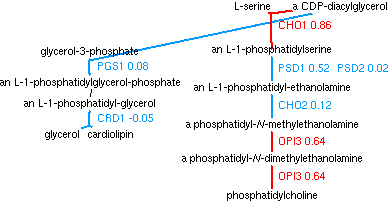 | | [phosphatidylserine synthase](http://pathway.yeastgenome.org/YEAST/NEW-IMAGE?type=ENZYME&object=YER026C-MONOMER) | [CHO1](http://db.yeastgenome.org/cgi-bin/locus.pl?locus=S000000828) | | --- | --- | | [phosphatidylserine decarboxylase, golgi/vacuole](http://pathway.yeastgenome.org/YEAST/NEW-IMAGE?type=ENZYME&object=YGR170W-MONOMER) | [PSD2](http://db.yeastgenome.org/cgi-bin/locus.pl?locus=S000003402) | | [phosphatidylserine decarboxylase, mitochondria](http://pathway.yeastgenome.org/YEAST/NEW-IMAGE?type=ENZYME&object=YNL169C-MONOMER) | [PSD1](http://db.yeastgenome.org/cgi-bin/locus.pl?locus=S000005113) | | [CHO2](http://pathway.yeastgenome.org/YEAST/NEW-IMAGE?type=ENZYME&object=YGR157W-MONOMER) | [CHO2](http://db.yeastgenome.org/cgi-bin/locus.pl?locus=S000003389) | | [OPI3](http://pathway.yeastgenome.org/YEAST/NEW-IMAGE?type=ENZYME&object=YJR073C-MONOMER) | [OPI3](http://db.yeastgenome.org/cgi-bin/locus.pl?locus=S000003834) | | [phosphatidylglycerolphosphate synthase](http://pathway.yeastgenome.org/YEAST/NEW-IMAGE?type=ENZYME&object=YCL004W-MONOMER) | [PGS1](http://db.yeastgenome.org/cgi-bin/locus.pl?locus=S000000510) | | [cardiolipin synthase](http://pathway.yeastgenome.org/YEAST/NEW-IMAGE?type=ENZYME&object=YDL142C-MONOMER) | [CRD1](http://db.yeastgenome.org/cgi-bin/locus.pl?locus=S000002301) | |
| 35 | [phosphatidylinositol biosynthesis](http://pathway.yeastgenome.org/YEAST/NEW-IMAGE?type=PATHWAY&object=PWY3O-3) | 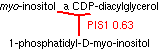 | | [phosphatidylinositol synthase](http://pathway.yeastgenome.org/YEAST/NEW-IMAGE?type=ENZYME&object=YPR113W-MONOMER) | [PIS1](http://db.yeastgenome.org/cgi-bin/locus.pl?locus=S000006317) | | --- | --- | |
| 40 | de novo NAD biosynthesis | 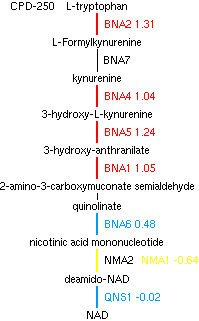 | | [Tryptophan 2,3-dioxygenase](http://pathway.yeastgenome.org/YEAST/NEW-IMAGE?type=ENZYME&object=YJR078W-MONOMER) | [BNA2](http://db.yeastgenome.org/cgi-bin/locus.pl?locus=S000003839) | | --- | --- | | [Arylformamidase](http://pathway.yeastgenome.org/YEAST/NEW-IMAGE?type=ENZYME&object=MONOMER3O-17) | [BNA7](http://db.yeastgenome.org/cgi-bin/locus.pl?locus=S000002836) | | [Kynurenine 3-mono oxygenase](http://pathway.yeastgenome.org/YEAST/NEW-IMAGE?type=ENZYME&object=YBL098W-MONOMER) | [BNA4](http://db.yeastgenome.org/cgi-bin/locus.pl?locus=S000000194) | | [Kynureninase](http://pathway.yeastgenome.org/YEAST/NEW-IMAGE?type=ENZYME&object=YLR231C-MONOMER) | [BNA5](http://db.yeastgenome.org/cgi-bin/locus.pl?locus=S000004221) | | [3-hydroxyanthranilic acid dioxygenase](http://pathway.yeastgenome.org/YEAST/NEW-IMAGE?type=ENZYME&object=YJR025C-MONOMER) | [BNA1](http://db.yeastgenome.org/cgi-bin/locus.pl?locus=S000003786) | | [Quinolinate phosphoribosyl transferase](http://pathway.yeastgenome.org/YEAST/NEW-IMAGE?type=ENZYME&object=YFR047C-MONOMER) | [BNA6](http://db.yeastgenome.org/cgi-bin/locus.pl?locus=S000001943) | | [Nicotinamide/nicotinic acid mononucleotide adenylyltransferase](http://pathway.yeastgenome.org/YEAST/NEW-IMAGE?type=ENZYME&object=YLR328W-MONOMER) | [NMA1](http://db.yeastgenome.org/cgi-bin/locus.pl?locus=S000004320) | | [Nicotinamide/nicotinic acid mononucleotide adenylyltransferase](http://pathway.yeastgenome.org/YEAST/NEW-IMAGE?type=ENZYME&object=YGR010W-MONOMER) | [NMA2](http://db.yeastgenome.org/cgi-bin/locus.pl?locus=S000003242) | | [glutamine-dependent NAD synthase](http://pathway.yeastgenome.org/YEAST/NEW-IMAGE?type=ENZYME&object=MONOMER3O-845) | [QNS1](http://db.yeastgenome.org/cgi-bin/locus.pl?locus=S000001116) | |
| 40 | [tryptophan degradation via kynurenine](http://pathway.yeastgenome.org/YEAST/NEW-IMAGE?type=PATHWAY&object=TRYPTOPHAN-DEGRADATION-1) | 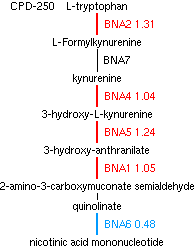 | | [Tryptophan 2,3-dioxygenase](http://pathway.yeastgenome.org/YEAST/NEW-IMAGE?type=ENZYME&object=YJR078W-MONOMER) | [BNA2](http://db.yeastgenome.org/cgi-bin/locus.pl?locus=S000003839) | | --- | --- | | [Arylformamidase](http://pathway.yeastgenome.org/YEAST/NEW-IMAGE?type=ENZYME&object=MONOMER3O-17) | [BNA7](http://db.yeastgenome.org/cgi-bin/locus.pl?locus=S000002836) | | [Kynurenine 3-mono oxygenase](http://pathway.yeastgenome.org/YEAST/NEW-IMAGE?type=ENZYME&object=YBL098W-MONOMER) | [BNA4](http://db.yeastgenome.org/cgi-bin/locus.pl?locus=S000000194) | | [Kynureninase](http://pathway.yeastgenome.org/YEAST/NEW-IMAGE?type=ENZYME&object=YLR231C-MONOMER) | [BNA5](http://db.yeastgenome.org/cgi-bin/locus.pl?locus=S000004221) | | [3-hydroxyanthranilic acid dioxygenase](http://pathway.yeastgenome.org/YEAST/NEW-IMAGE?type=ENZYME&object=YJR025C-MONOMER) | [BNA1](http://db.yeastgenome.org/cgi-bin/locus.pl?locus=S000003786) | | [Quinolinate phosphoribosyl transferase](http://pathway.yeastgenome.org/YEAST/NEW-IMAGE?type=ENZYME&object=YFR047C-MONOMER) | [BNA6](http://db.yeastgenome.org/cgi-bin/locus.pl?locus=S000001943) | |
| 40 | [nicotinamide riboside salvage pathway I](http://pathway.yeastgenome.org/YEAST/NEW-IMAGE?type=PATHWAY&object=PWY3O-4106) | 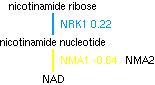 | | [nicotinic acid riboside kinase / nicotinamide ribose kinase](http://pathway.yeastgenome.org/YEAST/NEW-IMAGE?type=ENZYME&object=MONOMER3O-4139) | [NRK1](http://db.yeastgenome.org/cgi-bin/locus.pl?locus=S000005073) | | --- | --- | | [Nicotinamide/nicotinic acid mononucleotide adenylyltransferase](http://pathway.yeastgenome.org/YEAST/NEW-IMAGE?type=ENZYME&object=YGR010W-MONOMER) | [NMA2](http://db.yeastgenome.org/cgi-bin/locus.pl?locus=S000003242) | | [Nicotinamide/nicotinic acid mononucleotide adenylyltransferase](http://pathway.yeastgenome.org/YEAST/NEW-IMAGE?type=ENZYME&object=YLR328W-MONOMER) | [NMA1](http://db.yeastgenome.org/cgi-bin/locus.pl?locus=S000004320) | |
| 40 | [NAD salvage pathway](http://pathway.yeastgenome.org/YEAST/NEW-IMAGE?type=PATHWAY&object=PWY3O-4107) | 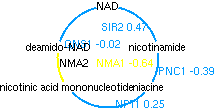 | | [Nicotinamide/nicotinic acid mononucleotide adenylyltransferase](http://pathway.yeastgenome.org/YEAST/NEW-IMAGE?type=ENZYME&object=YLR328W-MONOMER) | [NMA1](http://db.yeastgenome.org/cgi-bin/locus.pl?locus=S000004320) | | --- | --- | | [Nicotinamide/nicotinic acid mononucleotide adenylyltransferase](http://pathway.yeastgenome.org/YEAST/NEW-IMAGE?type=ENZYME&object=YGR010W-MONOMER) | [NMA2](http://db.yeastgenome.org/cgi-bin/locus.pl?locus=S000003242) | | [glutamine-dependent NAD synthase](http://pathway.yeastgenome.org/YEAST/NEW-IMAGE?type=ENZYME&object=MONOMER3O-845) | [QNS1](http://db.yeastgenome.org/cgi-bin/locus.pl?locus=S000001116) | | [NAD-dependent histone deacetylase](http://pathway.yeastgenome.org/YEAST/NEW-IMAGE?type=ENZYME&object=MONOMER3O-4152) | [SIR2](http://db.yeastgenome.org/cgi-bin/locus.pl?locus=S000002200) | | [nicotinamidase](http://pathway.yeastgenome.org/YEAST/NEW-IMAGE?type=ENZYME&object=YGL037C-MONOMER) | [PNC1](http://db.yeastgenome.org/cgi-bin/locus.pl?locus=S000003005) | | [nicotinate phosphoribosyl transferase](http://pathway.yeastgenome.org/YEAST/NEW-IMAGE?type=ENZYME&object=YOR209C-MONOMER) | [NPT1](http://db.yeastgenome.org/cgi-bin/locus.pl?locus=S000005735) | |
| 36 - 42 | folate biosynthesis | 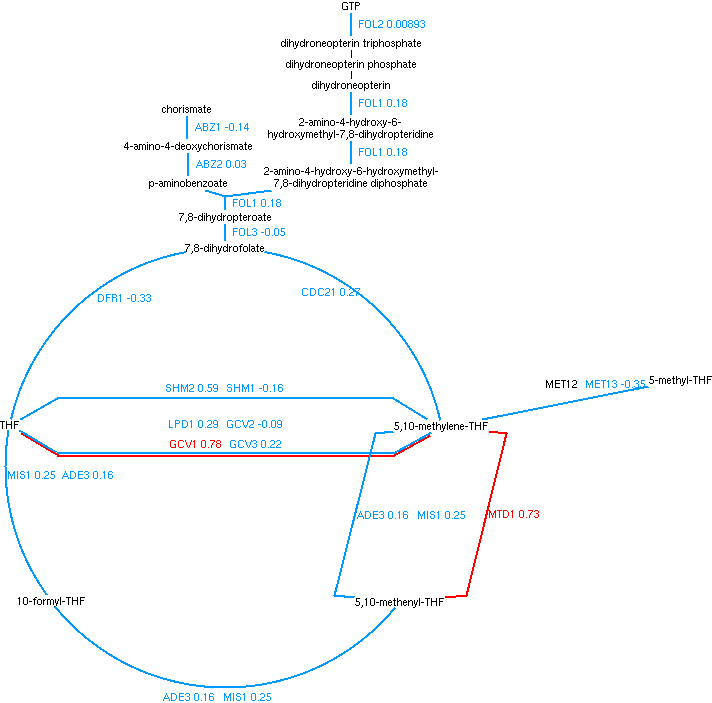 | | [GTP-cyclohydrolase I](http://pathway.yeastgenome.org/YEAST/NEW-IMAGE?type=ENZYME&object=YGR267C-MONOMER) | [FOL2](http://db.yeastgenome.org/cgi-bin/locus.pl?locus=S000003499) | | --- | --- | | [aminodeoxychorismate synthase](http://pathway.yeastgenome.org/YEAST/NEW-IMAGE?type=ENZYME&object=YNR033W-MONOMER) | [ABZ1](http://db.yeastgenome.org/cgi-bin/locus.pl?locus=S000005316) | | [aminodeoxychorismate lyase](http://pathway.yeastgenome.org/YEAST/NEW-IMAGE?type=ENZYME&object=MONOMER3O-131) | [ABZ2](http://db.yeastgenome.org/cgi-bin/locus.pl?locus=S000004902) | | [2-amino-4-hydroxy-6-hydroxymethyldihydropteridine pyrophosphokinase [multifunctional]](http://pathway.yeastgenome.org/YEAST/NEW-IMAGE?type=ENZYME&object=YNL256W-MONOMER) | [FOL1](http://db.yeastgenome.org/cgi-bin/locus.pl?locus=S000005200) | | [dihydrofolate synthase](http://pathway.yeastgenome.org/YEAST/NEW-IMAGE?type=ENZYME&object=YMR113W-MONOMER) | [FOL3](http://db.yeastgenome.org/cgi-bin/locus.pl?locus=S000004719) | | [dihydrofolate reductase](http://pathway.yeastgenome.org/YEAST/NEW-IMAGE?type=ENZYME&object=YOR236W-MONOMER) | [DFR1](http://db.yeastgenome.org/cgi-bin/locus.pl?locus=S000005762) | | [glycine cleavage complex](http://pathway.yeastgenome.org/YEAST/NEW-IMAGE?type=ENZYME&object=CPLX3O-213) | [LPD1](http://db.yeastgenome.org/cgi-bin/locus.pl?locus=S000001876) [GCV2](http://db.yeastgenome.org/cgi-bin/locus.pl?locus=S000004801) [GCV1](http://db.yeastgenome.org/cgi-bin/locus.pl?locus=S000002426) [GCV3](http://db.yeastgenome.org/cgi-bin/locus.pl?locus=S000000042) | | [Serine hydroxymethyltransferase, mitochondrial](http://pathway.yeastgenome.org/YEAST/NEW-IMAGE?type=ENZYME&object=YBR263W-MONOMER) | [SHM1](http://db.yeastgenome.org/cgi-bin/locus.pl?locus=S000000467) | | [serine hydroxymethyltransferase](http://pathway.yeastgenome.org/YEAST/NEW-IMAGE?type=ENZYME&object=YLR058C-MONOMER) | [SHM2](http://db.yeastgenome.org/cgi-bin/locus.pl?locus=S000004048) | | [thymidylate synthase](http://pathway.yeastgenome.org/YEAST/NEW-IMAGE?type=ENZYME&object=CPLX3O-630) | [CDC21](http://db.yeastgenome.org/cgi-bin/locus.pl?locus=S000005600) | | [MTHFR](http://pathway.yeastgenome.org/YEAST/NEW-IMAGE?type=ENZYME&object=YGL125W-MONOMER) | [MET13](http://db.yeastgenome.org/cgi-bin/locus.pl?locus=S000003093) | | [MTHFR](http://pathway.yeastgenome.org/YEAST/NEW-IMAGE?type=ENZYME&object=YPL023C-MONOMER) | [MET12](http://db.yeastgenome.org/cgi-bin/locus.pl?locus=S000005944) | | [NAD-dependent 5,10-methylenetetrahydrafolate dehydrogenase](http://pathway.yeastgenome.org/YEAST/NEW-IMAGE?type=ENZYME&object=CPLX3O-317) | [MTD1](http://db.yeastgenome.org/cgi-bin/locus.pl?locus=S000001788) | | [C1-tetrahydrofolate synthase](http://pathway.yeastgenome.org/YEAST/NEW-IMAGE?type=ENZYME&object=YGR204W-MONOMER) | [ADE3](http://db.yeastgenome.org/cgi-bin/locus.pl?locus=S000003436) | | [mitochondrial C1-tetrahydrofolate synthase](http://pathway.yeastgenome.org/YEAST/NEW-IMAGE?type=ENZYME&object=YBR084W-MONOMER) | [MIS1](http://db.yeastgenome.org/cgi-bin/locus.pl?locus=S000000288) | |
| 41 | [folate polyglutamylation](http://pathway.yeastgenome.org/YEAST/NEW-IMAGE?type=PATHWAY&object=PWY3O-20) | 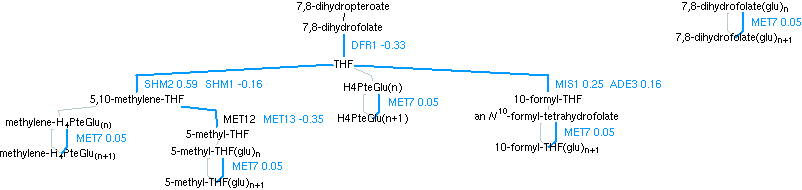 | | [dihydrofolate reductase](http://pathway.yeastgenome.org/YEAST/NEW-IMAGE?type=ENZYME&object=YOR236W-MONOMER) | [DFR1](http://db.yeastgenome.org/cgi-bin/locus.pl?locus=S000005762) | | --- | --- | | [Serine hydroxymethyltransferase, mitochondrial](http://pathway.yeastgenome.org/YEAST/NEW-IMAGE?type=ENZYME&object=YBR263W-MONOMER) | [SHM1](http://db.yeastgenome.org/cgi-bin/locus.pl?locus=S000000467) | | [serine hydroxymethyltransferase](http://pathway.yeastgenome.org/YEAST/NEW-IMAGE?type=ENZYME&object=YLR058C-MONOMER) | [SHM2](http://db.yeastgenome.org/cgi-bin/locus.pl?locus=S000004048) | | [MTHFR](http://pathway.yeastgenome.org/YEAST/NEW-IMAGE?type=ENZYME&object=YGL125W-MONOMER) | [MET13](http://db.yeastgenome.org/cgi-bin/locus.pl?locus=S000003093) | | [MTHFR](http://pathway.yeastgenome.org/YEAST/NEW-IMAGE?type=ENZYME&object=YPL023C-MONOMER) | [MET12](http://db.yeastgenome.org/cgi-bin/locus.pl?locus=S000005944) | | [C1-tetrahydrofolate synthase](http://pathway.yeastgenome.org/YEAST/NEW-IMAGE?type=ENZYME&object=YGR204W-MONOMER) | [ADE3](http://db.yeastgenome.org/cgi-bin/locus.pl?locus=S000003436) | | [mitochondrial C1-tetrahydrofolate synthase](http://pathway.yeastgenome.org/YEAST/NEW-IMAGE?type=ENZYME&object=YBR084W-MONOMER) | [MIS1](http://db.yeastgenome.org/cgi-bin/locus.pl?locus=S000000288) | | [folylpolyglutamate synthetase](http://pathway.yeastgenome.org/YEAST/NEW-IMAGE?type=ENZYME&object=YOR241W-MONOMER) | [MET7](http://db.yeastgenome.org/cgi-bin/locus.pl?locus=S000005767) | |
| 42 | [folate interconversions](http://pathway.yeastgenome.org/YEAST/NEW-IMAGE?type=PATHWAY&object=PWY3O-697) | 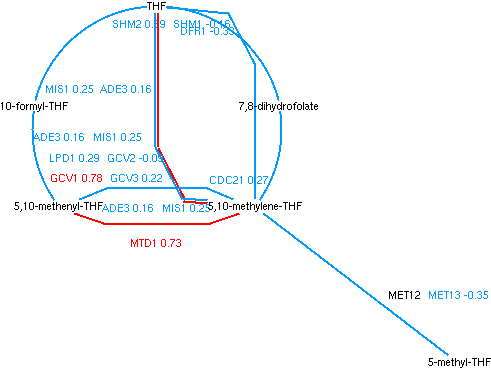 | | [C1-tetrahydrofolate synthase](http://pathway.yeastgenome.org/YEAST/NEW-IMAGE?type=ENZYME&object=YGR204W-MONOMER) | [ADE3](http://db.yeastgenome.org/cgi-bin/locus.pl?locus=S000003436) | | --- | --- | | [mitochondrial C1-tetrahydrofolate synthase](http://pathway.yeastgenome.org/YEAST/NEW-IMAGE?type=ENZYME&object=YBR084W-MONOMER) | [MIS1](http://db.yeastgenome.org/cgi-bin/locus.pl?locus=S000000288) | | [Serine hydroxymethyltransferase, mitochondrial](http://pathway.yeastgenome.org/YEAST/NEW-IMAGE?type=ENZYME&object=YBR263W-MONOMER) | [SHM1](http://db.yeastgenome.org/cgi-bin/locus.pl?locus=S000000467) | | [serine hydroxymethyltransferase](http://pathway.yeastgenome.org/YEAST/NEW-IMAGE?type=ENZYME&object=YLR058C-MONOMER) | [SHM2](http://db.yeastgenome.org/cgi-bin/locus.pl?locus=S000004048) | | [thymidylate synthase](http://pathway.yeastgenome.org/YEAST/NEW-IMAGE?type=ENZYME&object=CPLX3O-630) | [CDC21](http://db.yeastgenome.org/cgi-bin/locus.pl?locus=S000005600) | | [dihydrofolate reductase](http://pathway.yeastgenome.org/YEAST/NEW-IMAGE?type=ENZYME&object=YOR236W-MONOMER) | [DFR1](http://db.yeastgenome.org/cgi-bin/locus.pl?locus=S000005762) | | [glycine cleavage complex](http://pathway.yeastgenome.org/YEAST/NEW-IMAGE?type=ENZYME&object=CPLX3O-213) | [LPD1](http://db.yeastgenome.org/cgi-bin/locus.pl?locus=S000001876) [GCV2](http://db.yeastgenome.org/cgi-bin/locus.pl?locus=S000004801) [GCV1](http://db.yeastgenome.org/cgi-bin/locus.pl?locus=S000002426) [GCV3](http://db.yeastgenome.org/cgi-bin/locus.pl?locus=S000000042) | | [MTHFR](http://pathway.yeastgenome.org/YEAST/NEW-IMAGE?type=ENZYME&object=YGL125W-MONOMER) | [MET13](http://db.yeastgenome.org/cgi-bin/locus.pl?locus=S000003093) | | [MTHFR](http://pathway.yeastgenome.org/YEAST/NEW-IMAGE?type=ENZYME&object=YPL023C-MONOMER) | [MET12](http://db.yeastgenome.org/cgi-bin/locus.pl?locus=S000005944) | | [NAD-dependent 5,10-methylenetetrahydrafolate dehydrogenase](http://pathway.yeastgenome.org/YEAST/NEW-IMAGE?type=ENZYME&object=CPLX3O-317) | [MTD1](http://db.yeastgenome.org/cgi-bin/locus.pl?locus=S000001788) | |
| 44 | [folate transformations](http://pathway.yeastgenome.org/YEAST/NEW-IMAGE?type=PATHWAY&object=PWY-2201) | 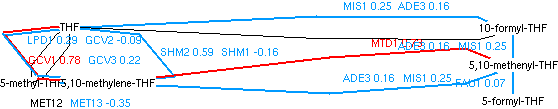 | | [Serine hydroxymethyltransferase, mitochondrial](http://pathway.yeastgenome.org/YEAST/NEW-IMAGE?type=ENZYME&object=YBR263W-MONOMER) | [SHM1](http://db.yeastgenome.org/cgi-bin/locus.pl?locus=S000000467) | | --- | --- | | [serine hydroxymethyltransferase](http://pathway.yeastgenome.org/YEAST/NEW-IMAGE?type=ENZYME&object=YLR058C-MONOMER) | [SHM2](http://db.yeastgenome.org/cgi-bin/locus.pl?locus=S000004048) | | [MTHFR](http://pathway.yeastgenome.org/YEAST/NEW-IMAGE?type=ENZYME&object=YGL125W-MONOMER) | [MET13](http://db.yeastgenome.org/cgi-bin/locus.pl?locus=S000003093) | | [MTHFR](http://pathway.yeastgenome.org/YEAST/NEW-IMAGE?type=ENZYME&object=YPL023C-MONOMER) | [MET12](http://db.yeastgenome.org/cgi-bin/locus.pl?locus=S000005944) | | [glycine cleavage complex](http://pathway.yeastgenome.org/YEAST/NEW-IMAGE?type=ENZYME&object=CPLX3O-213) | [LPD1](http://db.yeastgenome.org/cgi-bin/locus.pl?locus=S000001876) [GCV2](http://db.yeastgenome.org/cgi-bin/locus.pl?locus=S000004801) [GCV1](http://db.yeastgenome.org/cgi-bin/locus.pl?locus=S000002426) [GCV3](http://db.yeastgenome.org/cgi-bin/locus.pl?locus=S000000042) | | [NAD-dependent 5,10-methylenetetrahydrafolate dehydrogenase](http://pathway.yeastgenome.org/YEAST/NEW-IMAGE?type=ENZYME&object=CPLX3O-317) | [MTD1](http://db.yeastgenome.org/cgi-bin/locus.pl?locus=S000001788) | | [mitochondrial C1-tetrahydrofolate synthase](http://pathway.yeastgenome.org/YEAST/NEW-IMAGE?type=ENZYME&object=YBR084W-MONOMER) | [MIS1](http://db.yeastgenome.org/cgi-bin/locus.pl?locus=S000000288) | | [C1-tetrahydrofolate synthase](http://pathway.yeastgenome.org/YEAST/NEW-IMAGE?type=ENZYME&object=YGR204W-MONOMER) | [ADE3](http://db.yeastgenome.org/cgi-bin/locus.pl?locus=S000003436) | | [5,10-methenyltetrahydrofolate synthetase](http://pathway.yeastgenome.org/YEAST/NEW-IMAGE?type=ENZYME&object=YER183C-MONOMER) | [FAU1](http://db.yeastgenome.org/cgi-bin/locus.pl?locus=S000000985) | |
| 48 | [glutathione-glutaredoxin](http://pathway.yeastgenome.org/YEAST/NEW-IMAGE?type=PATHWAY&object=PLPSAL-PWY) redox reactions | 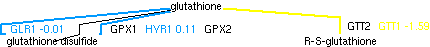 | | [glutathione oxidoreductase](http://pathway.yeastgenome.org/YEAST/NEW-IMAGE?type=ENZYME&object=YPL091W-MONOMER) | [GLR1](http://db.yeastgenome.org/cgi-bin/locus.pl?locus=S000006012) | | --- | --- | | [glutathione transferase](http://pathway.yeastgenome.org/YEAST/NEW-IMAGE?type=ENZYME&object=YIR038C-MONOMER) | [GTT1](http://db.yeastgenome.org/cgi-bin/locus.pl?locus=S000001477) | | [glutathione transferase](http://pathway.yeastgenome.org/YEAST/NEW-IMAGE?type=ENZYME&object=YLL060C-MONOMER) | [GTT2](http://db.yeastgenome.org/cgi-bin/locus.pl?locus=S000003983) | | [Glutathione peroxidase](http://pathway.yeastgenome.org/YEAST/NEW-IMAGE?type=ENZYME&object=YBR244W-MONOMER) | [GPX2](http://db.yeastgenome.org/cgi-bin/locus.pl?locus=S000000448) | | [glutathione-peroxidase](http://pathway.yeastgenome.org/YEAST/NEW-IMAGE?type=ENZYME&object=YIR037W-MONOMER) | [HYR1](http://db.yeastgenome.org/cgi-bin/locus.pl?locus=S000001476) | | [Glutathione peroxidase](http://pathway.yeastgenome.org/YEAST/NEW-IMAGE?type=ENZYME&object=YKL026C-MONOMER) | [GPX1](http://db.yeastgenome.org/cgi-bin/locus.pl?locus=S000001509) | |
| 49 | [de novo biosynthesis of purine nucleotides](http://pathway.yeastgenome.org/YEAST/NEW-IMAGE?type=PATHWAY&object=DENOVOPURINE3-PWY) | 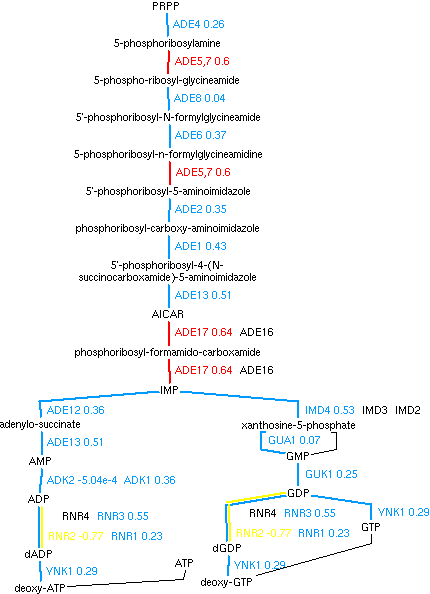 | | [phosphoribosylpyrophosphate amidotransferase](http://pathway.yeastgenome.org/YEAST/NEW-IMAGE?type=ENZYME&object=YMR300C-MONOMER) | [ADE4](http://db.yeastgenome.org/cgi-bin/locus.pl?locus=S000004915) | | --- | --- | | [glycinamide ribotide transformylase](http://pathway.yeastgenome.org/YEAST/NEW-IMAGE?type=ENZYME&object=YDR408C-MONOMER) | [ADE8](http://db.yeastgenome.org/cgi-bin/locus.pl?locus=S000002816) | | [5'-phosphoribosylformyl glycinamidine synthetase](http://pathway.yeastgenome.org/YEAST/NEW-IMAGE?type=ENZYME&object=YGR061C-MONOMER) | [ADE6](http://db.yeastgenome.org/cgi-bin/locus.pl?locus=S000003293) | | [phosphoribosylglycinamidine synthetase / phopshoribosylaminoimidazole synthetase](http://pathway.yeastgenome.org/YEAST/NEW-IMAGE?type=ENZYME&object=YGL234W-MONOMER) | [ADE5,7](http://db.yeastgenome.org/cgi-bin/locus.pl?locus=S000003203) | | [phosphoribosylaminoimidazole-carboxylase](http://pathway.yeastgenome.org/YEAST/NEW-IMAGE?type=ENZYME&object=YOR128C-MONOMER) | [ADE2](http://db.yeastgenome.org/cgi-bin/locus.pl?locus=S000005654) | | [phosphoribosyl amino imidazolesuccinocarbozamide synthetase](http://pathway.yeastgenome.org/YEAST/NEW-IMAGE?type=ENZYME&object=YAR015W-MONOMER) | [ADE1](http://db.yeastgenome.org/cgi-bin/locus.pl?locus=S000000070) | | [inosine monophosphate cyclohydrolase [multifunctional]](http://pathway.yeastgenome.org/YEAST/NEW-IMAGE?type=ENZYME&object=YLR028C-MONOMER) | [ADE16](http://db.yeastgenome.org/cgi-bin/locus.pl?locus=S000004018) | | [inosine monophosphate cyclohydrolase [multifunctional]](http://pathway.yeastgenome.org/YEAST/NEW-IMAGE?type=ENZYME&object=YMR120C-MONOMER) | [ADE17](http://db.yeastgenome.org/cgi-bin/locus.pl?locus=S000004727) | | [IMP dehydrogenase](http://pathway.yeastgenome.org/YEAST/NEW-IMAGE?type=ENZYME&object=YHR216W-MONOMER) | [IMD2](http://db.yeastgenome.org/cgi-bin/locus.pl?locus=S000001259) | | [IMP dehydrogenase](http://pathway.yeastgenome.org/YEAST/NEW-IMAGE?type=ENZYME&object=YLR432W-MONOMER) | [IMD3](http://db.yeastgenome.org/cgi-bin/locus.pl?locus=S000004424) | | [IMP dehydrogenase](http://pathway.yeastgenome.org/YEAST/NEW-IMAGE?type=ENZYME&object=YML056C-MONOMER) | [IMD4](http://db.yeastgenome.org/cgi-bin/locus.pl?locus=S000004520) | | [GMP synthase](http://pathway.yeastgenome.org/YEAST/NEW-IMAGE?type=ENZYME&object=YMR217W-MONOMER) | [GUA1](http://db.yeastgenome.org/cgi-bin/locus.pl?locus=S000004830) | | [guanylate kinase](http://pathway.yeastgenome.org/YEAST/NEW-IMAGE?type=ENZYME&object=MONOMER3O-102) | [GUK1](http://db.yeastgenome.org/cgi-bin/locus.pl?locus=S000002862) | | [adenylosuccinate synthetase](http://pathway.yeastgenome.org/YEAST/NEW-IMAGE?type=ENZYME&object=YNL220W-MONOMER) | [ADE12](http://db.yeastgenome.org/cgi-bin/locus.pl?locus=S000005164) | | [adenylosuccinate lyase](http://pathway.yeastgenome.org/YEAST/NEW-IMAGE?type=ENZYME&object=YLR359W-MONOMER) | [ADE13](http://db.yeastgenome.org/cgi-bin/locus.pl?locus=S000004351) | | [adenylate kinase](http://pathway.yeastgenome.org/YEAST/NEW-IMAGE?type=ENZYME&object=YDR226W-MONOMER) | [ADK1](http://db.yeastgenome.org/cgi-bin/locus.pl?locus=S000002634) | | [mitochondrial GTP:AMP phosphotransferase](http://pathway.yeastgenome.org/YEAST/NEW-IMAGE?type=ENZYME&object=YER170W-MONOMER) | [ADK2](http://db.yeastgenome.org/cgi-bin/locus.pl?locus=S000000972) | | [ribonucleotide reductase](http://pathway.yeastgenome.org/YEAST/NEW-IMAGE?type=ENZYME&object=CPLX3O-270) | [RNR4](http://db.yeastgenome.org/cgi-bin/locus.pl?locus=S000003412) [RNR3](http://db.yeastgenome.org/cgi-bin/locus.pl?locus=S000001328) [RNR2](http://db.yeastgenome.org/cgi-bin/locus.pl?locus=S000003563) [RNR1](http://db.yeastgenome.org/cgi-bin/locus.pl?locus=S000000872) | | [nucleoside diphosphate kinase](http://pathway.yeastgenome.org/YEAST/NEW-IMAGE?type=ENZYME&object=YKL067W-MONOMER) | [YNK1](http://db.yeastgenome.org/cgi-bin/locus.pl?locus=S000001550) | |
| 51 | [de novo biosynthesis of pyrimidine deoxyribonucleotides](http://pathway.yeastgenome.org/YEAST/NEW-IMAGE?type=PATHWAY&object=YEAST-SALV-PYRMID-DNTP) | 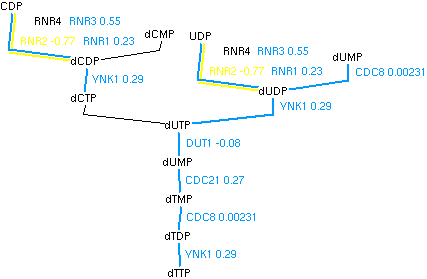 | | [ribonucleotide reductase](http://pathway.yeastgenome.org/YEAST/NEW-IMAGE?type=ENZYME&object=CPLX3O-270) | [RNR4](http://db.yeastgenome.org/cgi-bin/locus.pl?locus=S000003412) [RNR3](http://db.yeastgenome.org/cgi-bin/locus.pl?locus=S000001328) [RNR2](http://db.yeastgenome.org/cgi-bin/locus.pl?locus=S000003563) [RNR1](http://db.yeastgenome.org/cgi-bin/locus.pl?locus=S000000872) | | --- | --- | | [dUTP pyrophosphatase](http://pathway.yeastgenome.org/YEAST/NEW-IMAGE?type=ENZYME&object=YBR252W-MONOMER) | [DUT1](http://db.yeastgenome.org/cgi-bin/locus.pl?locus=S000000456) | | [thymidylate synthase](http://pathway.yeastgenome.org/YEAST/NEW-IMAGE?type=ENZYME&object=CPLX3O-630) | [CDC21](http://db.yeastgenome.org/cgi-bin/locus.pl?locus=S000005600) | | [uridylate kinase / thymidylate kinase](http://pathway.yeastgenome.org/YEAST/NEW-IMAGE?type=ENZYME&object=YJR057W-MONOMER) | [CDC8](http://db.yeastgenome.org/cgi-bin/locus.pl?locus=S000003818) | | [nucleoside diphosphate kinase](http://pathway.yeastgenome.org/YEAST/NEW-IMAGE?type=ENZYME&object=YKL067W-MONOMER) | [YNK1](http://db.yeastgenome.org/cgi-bin/locus.pl?locus=S000001550) | |
| 58 | [*myo*-inositol biosynthesis](http://pathway.yeastgenome.org/YEAST/NEW-IMAGE?type=PATHWAY&object=PWY-2301) | 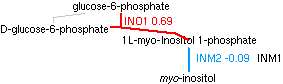 | | [L-myo-inositol-1-phosphate synthase](http://pathway.yeastgenome.org/YEAST/NEW-IMAGE?type=ENZYME&object=CPLX3O-29) | [INO1](http://db.yeastgenome.org/cgi-bin/locus.pl?locus=S000003689) | | --- | --- | | [inositol monophosphatase](http://pathway.yeastgenome.org/YEAST/NEW-IMAGE?type=ENZYME&object=YHR046C-MONOMER) | [INM1](http://db.yeastgenome.org/cgi-bin/locus.pl?locus=S000001088) | | [inositol monophosphate](http://pathway.yeastgenome.org/YEAST/NEW-IMAGE?type=ENZYME&object=MONOMER3O-11) | [INM2](http://db.yeastgenome.org/cgi-bin/locus.pl?locus=S000002695) | |
| 60 | [chitosan biosynthesis](http://pathway.yeastgenome.org/YEAST/NEW-IMAGE?type=PATHWAY&object=TRESYN-PWY) | 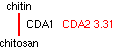 | | [chitin deacetylase](http://pathway.yeastgenome.org/YEAST/NEW-IMAGE?type=ENZYME&object=YLR308W-MONOMER) | [CDA2](http://db.yeastgenome.org/cgi-bin/locus.pl?locus=S000004299) | | --- | --- | | [chitin deacetylase](http://pathway.yeastgenome.org/YEAST/NEW-IMAGE?type=ENZYME&object=YLR307W-MONOMER) | [CDA1](http://db.yeastgenome.org/cgi-bin/locus.pl?locus=S000004298) | |
| 61 | [gluconeogenesis](http://pathway.yeastgenome.org/YEAST/NEW-IMAGE?type=PATHWAY&object=GLUCONEO-PWY) | 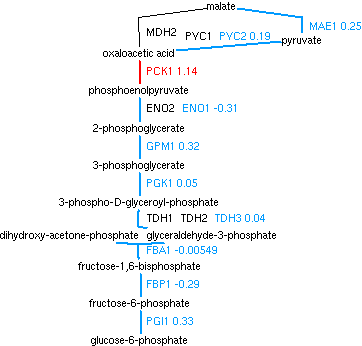 | | [malic enzyme](http://pathway.yeastgenome.org/YEAST/NEW-IMAGE?type=ENZYME&object=YKL029C-MONOMER) | [MAE1](http://db.yeastgenome.org/cgi-bin/locus.pl?locus=S000001512) | | --- | --- | | [pyruvate carboxylase](http://pathway.yeastgenome.org/YEAST/NEW-IMAGE?type=ENZYME&object=YBR218C-MONOMER) | [PYC2](http://db.yeastgenome.org/cgi-bin/locus.pl?locus=S000000422) | | [pyruvate carboxylase](http://pathway.yeastgenome.org/YEAST/NEW-IMAGE?type=ENZYME&object=YGL062W-MONOMER) | [PYC1](http://db.yeastgenome.org/cgi-bin/locus.pl?locus=S000003030) | | [cytosolic malate dehydrogenase](http://pathway.yeastgenome.org/YEAST/NEW-IMAGE?type=ENZYME&object=CPLX3O-88) | [MDH2](http://db.yeastgenome.org/cgi-bin/locus.pl?locus=S000005486) | | [phosphoenolpyruvate carboxylkinase](http://pathway.yeastgenome.org/YEAST/NEW-IMAGE?type=ENZYME&object=YKR097W-MONOMER) | [PCK1](http://db.yeastgenome.org/cgi-bin/locus.pl?locus=S000001805) | | [enolase I](http://pathway.yeastgenome.org/YEAST/NEW-IMAGE?type=ENZYME&object=YGR254W-MONOMER) | [ENO1](http://db.yeastgenome.org/cgi-bin/locus.pl?locus=S000003486) | | [enolase](http://pathway.yeastgenome.org/YEAST/NEW-IMAGE?type=ENZYME&object=YHR174W-MONOMER) | [ENO2](http://db.yeastgenome.org/cgi-bin/locus.pl?locus=S000001217) | | [phosphoglycerate mutase](http://pathway.yeastgenome.org/YEAST/NEW-IMAGE?type=ENZYME&object=YKL152C-MONOMER) | [GPM1](http://db.yeastgenome.org/cgi-bin/locus.pl?locus=S000001635) | | [3-phosphoglycerate kinase](http://pathway.yeastgenome.org/YEAST/NEW-IMAGE?type=ENZYME&object=YCR012W-MONOMER) | [PGK1](http://db.yeastgenome.org/cgi-bin/locus.pl?locus=S000000605) | | [glyceraldehyde-3-phosphate dehydrogenase](http://pathway.yeastgenome.org/YEAST/NEW-IMAGE?type=ENZYME&object=YGR192C-MONOMER) | [TDH3](http://db.yeastgenome.org/cgi-bin/locus.pl?locus=S000003424) | | [glyceraldehyde 3-phosphate dehydrogenase](http://pathway.yeastgenome.org/YEAST/NEW-IMAGE?type=ENZYME&object=YJR009C-MONOMER) | [TDH2](http://db.yeastgenome.org/cgi-bin/locus.pl?locus=S000003769) | | [glyceraldehyde-3-phosphate dehydrogenase](http://pathway.yeastgenome.org/YEAST/NEW-IMAGE?type=ENZYME&object=YJL052W-MONOMER) | [TDH1](http://db.yeastgenome.org/cgi-bin/locus.pl?locus=S000003588) | | [aldolase](http://pathway.yeastgenome.org/YEAST/NEW-IMAGE?type=ENZYME&object=YKL060C-MONOMER) | [FBA1](http://db.yeastgenome.org/cgi-bin/locus.pl?locus=S000001543) | | [fructose-1,6-bisphosphatase](http://pathway.yeastgenome.org/YEAST/NEW-IMAGE?type=ENZYME&object=YLR377C-MONOMER) | [FBP1](http://db.yeastgenome.org/cgi-bin/locus.pl?locus=S000004369) | | [glucose-6-phosphate isomerase](http://pathway.yeastgenome.org/YEAST/NEW-IMAGE?type=ENZYME&object=YBR196C-MONOMER) | [PGI1](http://db.yeastgenome.org/cgi-bin/locus.pl?locus=S000000400) | |
| 64 | [trehalose biosynthesis](http://pathway.yeastgenome.org/YEAST/NEW-IMAGE?type=PATHWAY&object=TRESYN-PWY) | 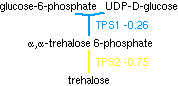 | | [trehalose-6-phosphate synthase](http://pathway.yeastgenome.org/YEAST/NEW-IMAGE?type=ENZYME&object=YBR126C-MONOMER) | [TPS1](http://db.yeastgenome.org/cgi-bin/locus.pl?locus=S000000330) | | --- | --- | | [trehalose-6-phosphate phosphatase](http://pathway.yeastgenome.org/YEAST/NEW-IMAGE?type=ENZYME&object=YDR074W-MONOMER) | [TPS2](http://db.yeastgenome.org/cgi-bin/locus.pl?locus=S000002481) | |
| 66 | [glyoxylate cycle](http://pathway.yeastgenome.org/YEAST/NEW-IMAGE?type=PATHWAY&object=GLYOXYLATE-BYPASS) | 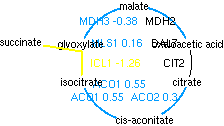 | | [cytosolic malate dehydrogenase](http://pathway.yeastgenome.org/YEAST/NEW-IMAGE?type=ENZYME&object=CPLX3O-88) | [MDH2](http://db.yeastgenome.org/cgi-bin/locus.pl?locus=S000005486) | | --- | --- | | [peroxisome malate dehydrogenase](http://pathway.yeastgenome.org/YEAST/NEW-IMAGE?type=ENZYME&object=CPLX3O-83) | [MDH3](http://db.yeastgenome.org/cgi-bin/locus.pl?locus=S000002236) | | [citrate synthase](http://pathway.yeastgenome.org/YEAST/NEW-IMAGE?type=ENZYME&object=YCR005C-MONOMER) | [CIT2](http://db.yeastgenome.org/cgi-bin/locus.pl?locus=S000000598) | | [aconitate hydratase](http://pathway.yeastgenome.org/YEAST/NEW-IMAGE?type=ENZYME&object=YJL200C-MONOMER) | [ACO2](http://db.yeastgenome.org/cgi-bin/locus.pl?locus=S000003736) | | [aconitase](http://pathway.yeastgenome.org/YEAST/NEW-IMAGE?type=ENZYME&object=YLR304C-MONOMER) | [ACO1](http://db.yeastgenome.org/cgi-bin/locus.pl?locus=S000004295) | | [isocitrate lyase](http://pathway.yeastgenome.org/YEAST/NEW-IMAGE?type=ENZYME&object=YER065C-MONOMER) | [ICL1](http://db.yeastgenome.org/cgi-bin/locus.pl?locus=S000000867) | | [malate synthase 2](http://pathway.yeastgenome.org/YEAST/NEW-IMAGE?type=ENZYME&object=YIR031C-MONOMER) | [DAL7](http://db.yeastgenome.org/cgi-bin/locus.pl?locus=S000001470) | | [malate synthase](http://pathway.yeastgenome.org/YEAST/NEW-IMAGE?type=ENZYME&object=YNL117W-MONOMER) | [MLS1](http://db.yeastgenome.org/cgi-bin/locus.pl?locus=S000005061) | |
| 67 | oxidative branch of the pentose phosphate pathway | 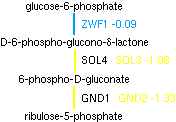 | | [glucose-6-phosphate dehydrogenase](http://pathway.yeastgenome.org/YEAST/NEW-IMAGE?type=ENZYME&object=YNL241C-MONOMER) | [ZWF1](http://db.yeastgenome.org/cgi-bin/locus.pl?locus=S000005185) | | --- | --- | | [6-phosphogluconolactonase](http://pathway.yeastgenome.org/YEAST/NEW-IMAGE?type=ENZYME&object=MONOMER3O-4032) | [SOL3](http://db.yeastgenome.org/cgi-bin/locus.pl?locus=S000001206) | | [6-phosphogluconolactonase](http://pathway.yeastgenome.org/YEAST/NEW-IMAGE?type=ENZYME&object=MONOMER3O-4047) | [SOL4](http://db.yeastgenome.org/cgi-bin/locus.pl?locus=S000003480) | | [6-phosphogluconate dehydrogenase](http://pathway.yeastgenome.org/YEAST/NEW-IMAGE?type=ENZYME&object=YGR256W-MONOMER) | [GND2](http://db.yeastgenome.org/cgi-bin/locus.pl?locus=S000003488) | | [6-phosphogluconate dehydrogenase, decarboxylating](http://pathway.yeastgenome.org/YEAST/NEW-IMAGE?type=ENZYME&object=YHR183W-MONOMER) | [GND1](http://db.yeastgenome.org/cgi-bin/locus.pl?locus=S000001226) | |
| 67 | [non-oxidative branch of the pentose phosphate pathway](http://pathway.yeastgenome.org/YEAST/NEW-IMAGE?type=PATHWAY&object=NONOXIPENT-PWY) | 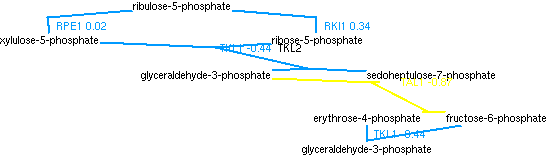 | | [ribose-5-phosphate ketol-isomerase](http://pathway.yeastgenome.org/YEAST/NEW-IMAGE?type=ENZYME&object=YOR095C-MONOMER) | [RKI1](http://db.yeastgenome.org/cgi-bin/locus.pl?locus=S000005621) | | --- | --- | | [D-ribulose-5-Phosphate 3-epimerase](http://pathway.yeastgenome.org/YEAST/NEW-IMAGE?type=ENZYME&object=YJL121C-MONOMER) | [RPE1](http://db.yeastgenome.org/cgi-bin/locus.pl?locus=S000003657) | | [transketolase](http://pathway.yeastgenome.org/YEAST/NEW-IMAGE?type=ENZYME&object=YBR117C-MONOMER) | [TKL2](http://db.yeastgenome.org/cgi-bin/locus.pl?locus=S000000321) | | [transaldolase](http://pathway.yeastgenome.org/YEAST/NEW-IMAGE?type=ENZYME&object=YLR354C-MONOMER) | [TAL1](http://db.yeastgenome.org/cgi-bin/locus.pl?locus=S000004346) | | [transketolase](http://pathway.yeastgenome.org/YEAST/NEW-IMAGE?type=ENZYME&object=YPR074C-MONOMER) | [TKL1](http://db.yeastgenome.org/cgi-bin/locus.pl?locus=S000006278) | |
| 68 | [aerobic respiration, electron transport chain](http://pathway.yeastgenome.org/YEAST/NEW-IMAGE?type=PATHWAY&object=PWY3O-188) | 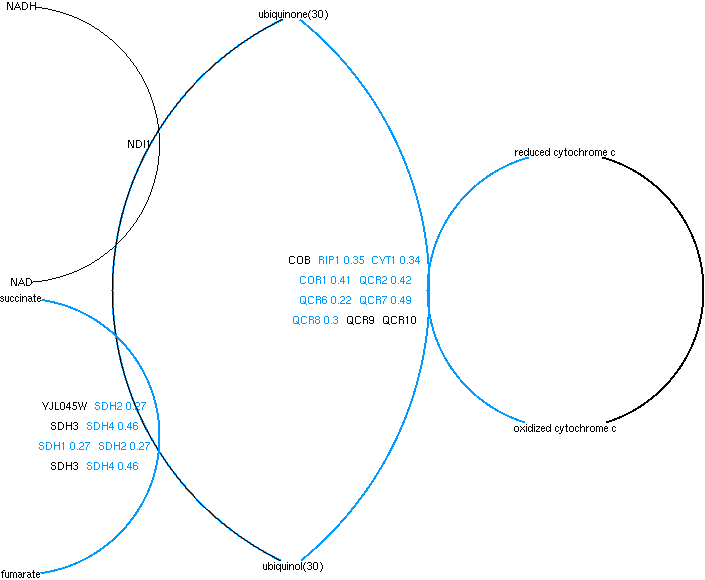 | | [cytochrome c oxidase](http://pathway.yeastgenome.org/YEAST/NEW-IMAGE?type=ENZYME&object=CPLX3O-117) | [COX1](http://db.yeastgenome.org/cgi-bin/locus.pl?locus=S000007260) [COX2](http://db.yeastgenome.org/cgi-bin/locus.pl?locus=S000007281) [COX3](http://db.yeastgenome.org/cgi-bin/locus.pl?locus=S000007283) [COX4](http://db.yeastgenome.org/cgi-bin/locus.pl?locus=S000003155) [COX5A](http://db.yeastgenome.org/cgi-bin/locus.pl?locus=S000004997) [COX6](http://db.yeastgenome.org/cgi-bin/locus.pl?locus=S000001093) [COX7](http://db.yeastgenome.org/cgi-bin/locus.pl?locus=S000004869) [COX8](http://db.yeastgenome.org/cgi-bin/locus.pl?locus=S000004387) [COX9](http://db.yeastgenome.org/cgi-bin/locus.pl?locus=S000002225) [COX12](http://db.yeastgenome.org/cgi-bin/locus.pl?locus=S000004028) [COX13](http://db.yeastgenome.org/cgi-bin/locus.pl?locus=S000003159) | | --- | --- | | [ubiquinol cytochrome c reductase complex](http://pathway.yeastgenome.org/YEAST/NEW-IMAGE?type=ENZYME&object=CPLX3O-109) | [COB](http://db.yeastgenome.org/cgi-bin/locus.pl?locus=S000007270) [RIP1](http://db.yeastgenome.org/cgi-bin/locus.pl?locus=S000000750) [CYT1](http://pathway.yeastgenome.org/YEAST/NEW-IMAGE?type=GENE&object=G3O-102) [COR1](http://db.yeastgenome.org/cgi-bin/locus.pl?locus=S000000141) [QCR2](http://db.yeastgenome.org/cgi-bin/locus.pl?locus=S000006395) [QCR6](http://db.yeastgenome.org/cgi-bin/locus.pl?locus=S000001929) [QCR7](http://db.yeastgenome.org/cgi-bin/locus.pl?locus=S000002937) [QCR8](http://db.yeastgenome.org/cgi-bin/locus.pl?locus=S000003702) [QCR9](http://db.yeastgenome.org/cgi-bin/locus.pl?locus=S000003415) [QCR10](http://db.yeastgenome.org/cgi-bin/locus.pl?locus=S000003529) | | [succinate dehydrogenase (ubiquinone)](http://pathway.yeastgenome.org/YEAST/NEW-IMAGE?type=ENZYME&object=CPLX3O-742) | [SDH1](http://db.yeastgenome.org/cgi-bin/locus.pl?locus=S000001631) [SDH2](http://db.yeastgenome.org/cgi-bin/locus.pl?locus=S000003964) [SDH3](http://db.yeastgenome.org/cgi-bin/locus.pl?locus=S000001624) [SDH4](http://db.yeastgenome.org/cgi-bin/locus.pl?locus=S000002585) | | [minor succinate dehydrogenase (ubiquinone)](http://pathway.yeastgenome.org/YEAST/NEW-IMAGE?type=ENZYME&object=CPLX3O-44) | [YJL045W](http://db.yeastgenome.org/cgi-bin/locus.pl?locus=S000003581) [SDH2](http://db.yeastgenome.org/cgi-bin/locus.pl?locus=S000003964) [SDH3](http://db.yeastgenome.org/cgi-bin/locus.pl?locus=S000001624) [SDH4](http://db.yeastgenome.org/cgi-bin/locus.pl?locus=S000002585) | | [NADH dehydrogenase (ubiquinone)](http://pathway.yeastgenome.org/YEAST/NEW-IMAGE?type=ENZYME&object=YML120C-MONOMER) | [NDI1](http://db.yeastgenome.org/cgi-bin/locus.pl?locus=S000004589) | |
| 69 | [superpathway of glucose fermentation](http://pathway.yeastgenome.org/YEAST/NEW-IMAGE?type=PATHWAY&object=GLUCFERMEN-PWY) | 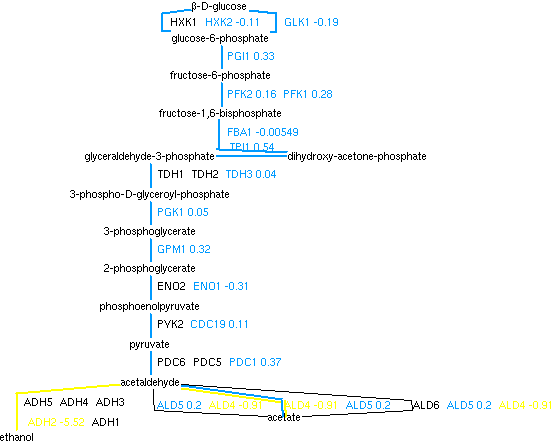 | | [glucokinase](http://pathway.yeastgenome.org/YEAST/NEW-IMAGE?type=ENZYME&object=YCL040W-MONOMER) | [GLK1](http://db.yeastgenome.org/cgi-bin/locus.pl?locus=S000000545) | | --- | --- | | [hexokinase II](http://pathway.yeastgenome.org/YEAST/NEW-IMAGE?type=ENZYME&object=YGL253W-MONOMER) | [HXK2](http://db.yeastgenome.org/cgi-bin/locus.pl?locus=S000003222) | | [hexokinase I](http://pathway.yeastgenome.org/YEAST/NEW-IMAGE?type=ENZYME&object=YFR053C-MONOMER) | [HXK1](http://db.yeastgenome.org/cgi-bin/locus.pl?locus=S000001949) | | [glucose-6-phosphate isomerase](http://pathway.yeastgenome.org/YEAST/NEW-IMAGE?type=ENZYME&object=YBR196C-MONOMER) | [PGI1](http://db.yeastgenome.org/cgi-bin/locus.pl?locus=S000000400) | | [phosphofructokinase](http://pathway.yeastgenome.org/YEAST/NEW-IMAGE?type=ENZYME&object=CPLX3O-77) | [PFK2](http://db.yeastgenome.org/cgi-bin/locus.pl?locus=S000004818) [PFK1](http://db.yeastgenome.org/cgi-bin/locus.pl?locus=S000003472) | | [aldolase](http://pathway.yeastgenome.org/YEAST/NEW-IMAGE?type=ENZYME&object=YKL060C-MONOMER) | [FBA1](http://db.yeastgenome.org/cgi-bin/locus.pl?locus=S000001543) | | [triosephosphate isomerase](http://pathway.yeastgenome.org/YEAST/NEW-IMAGE?type=ENZYME&object=YDR050C-MONOMER) | [TPI1](http://db.yeastgenome.org/cgi-bin/locus.pl?locus=S000002457) | | [glyceraldehyde-3-phosphate dehydrogenase](http://pathway.yeastgenome.org/YEAST/NEW-IMAGE?type=ENZYME&object=YGR192C-MONOMER) | [TDH3](http://db.yeastgenome.org/cgi-bin/locus.pl?locus=S000003424) | | [glyceraldehyde 3-phosphate dehydrogenase](http://pathway.yeastgenome.org/YEAST/NEW-IMAGE?type=ENZYME&object=YJR009C-MONOMER) | [TDH2](http://db.yeastgenome.org/cgi-bin/locus.pl?locus=S000003769) | | [glyceraldehyde-3-phosphate dehydrogenase](http://pathway.yeastgenome.org/YEAST/NEW-IMAGE?type=ENZYME&object=YJL052W-MONOMER) | [TDH1](http://db.yeastgenome.org/cgi-bin/locus.pl?locus=S000003588) | | [3-phosphoglycerate kinase](http://pathway.yeastgenome.org/YEAST/NEW-IMAGE?type=ENZYME&object=YCR012W-MONOMER) | [PGK1](http://db.yeastgenome.org/cgi-bin/locus.pl?locus=S000000605) | | [phosphoglycerate mutase](http://pathway.yeastgenome.org/YEAST/NEW-IMAGE?type=ENZYME&object=YKL152C-MONOMER) | [GPM1](http://db.yeastgenome.org/cgi-bin/locus.pl?locus=S000001635) | | [enolase I](http://pathway.yeastgenome.org/YEAST/NEW-IMAGE?type=ENZYME&object=YGR254W-MONOMER) | [ENO1](http://db.yeastgenome.org/cgi-bin/locus.pl?locus=S000003486) | | [enolase](http://pathway.yeastgenome.org/YEAST/NEW-IMAGE?type=ENZYME&object=YHR174W-MONOMER) | [ENO2](http://db.yeastgenome.org/cgi-bin/locus.pl?locus=S000001217) | | [pyruvate kinase](http://pathway.yeastgenome.org/YEAST/NEW-IMAGE?type=ENZYME&object=YAL038W-MONOMER) | [CDC19](http://db.yeastgenome.org/cgi-bin/locus.pl?locus=S000000036) | | [pyruvate kinase](http://pathway.yeastgenome.org/YEAST/NEW-IMAGE?type=ENZYME&object=YOR347C-MONOMER) | [PYK2](http://db.yeastgenome.org/cgi-bin/locus.pl?locus=S000005874) | | [pyruvate decarboxylase / decarboxylase](http://pathway.yeastgenome.org/YEAST/NEW-IMAGE?type=ENZYME&object=CPLX3O-118) | [PDC1](http://db.yeastgenome.org/cgi-bin/locus.pl?locus=S000004034) | | [pyruvate decarboxylase / decarboxylase](http://pathway.yeastgenome.org/YEAST/NEW-IMAGE?type=ENZYME&object=CPLX3O-67) | [PDC5](http://db.yeastgenome.org/cgi-bin/locus.pl?locus=S000004124) | | [pyruvate decarboxylase / decarboxylase](http://pathway.yeastgenome.org/YEAST/NEW-IMAGE?type=ENZYME&object=CPLX3O-58) | [PDC6](http://db.yeastgenome.org/cgi-bin/locus.pl?locus=S000003319) | | [aldehyde dehydrogenase (major mitochondrial)](http://pathway.yeastgenome.org/YEAST/NEW-IMAGE?type=ENZYME&object=YOR374W-MONOMER) | [ALD4](http://db.yeastgenome.org/cgi-bin/locus.pl?locus=S000005901) | | [aldehyde dehydrogenase (minor mitochondrial)](http://pathway.yeastgenome.org/YEAST/NEW-IMAGE?type=ENZYME&object=YER073W-MONOMER) | [ALD5](http://db.yeastgenome.org/cgi-bin/locus.pl?locus=S000000875) | | [aldehyde dehydrogenase (major cytoplasmic)](http://pathway.yeastgenome.org/YEAST/NEW-IMAGE?type=ENZYME&object=YPL061W-MONOMER) | [ALD6](http://db.yeastgenome.org/cgi-bin/locus.pl?locus=S000005982) | | [alcohol dehydrogenase](http://pathway.yeastgenome.org/YEAST/NEW-IMAGE?type=ENZYME&object=YOL086C-MONOMER) | [ADH1](http://db.yeastgenome.org/cgi-bin/locus.pl?locus=S000005446) | | [alcohol dehydrogenase](http://pathway.yeastgenome.org/YEAST/NEW-IMAGE?type=ENZYME&object=YMR303C-MONOMER) | [ADH2](http://db.yeastgenome.org/cgi-bin/locus.pl?locus=S000004918) | | [alcohol dehydrogenase](http://pathway.yeastgenome.org/YEAST/NEW-IMAGE?type=ENZYME&object=YMR083W-MONOMER) | [ADH3](http://db.yeastgenome.org/cgi-bin/locus.pl?locus=S000004688) | | [alcohol dehydrogenase](http://pathway.yeastgenome.org/YEAST/NEW-IMAGE?type=ENZYME&object=YGL256W-MONOMER) | [ADH4](http://db.yeastgenome.org/cgi-bin/locus.pl?locus=S000003225) | | [alcohol dehydrogenase](http://pathway.yeastgenome.org/YEAST/NEW-IMAGE?type=ENZYME&object=YBR145W-MONOMER) | [ADH5](http://db.yeastgenome.org/cgi-bin/locus.pl?locus=S000000349) | |
| 71 | [fatty acid oxidation pathway](http://pathway.yeastgenome.org/YEAST/NEW-IMAGE?type=PATHWAY&object=YEAST-FAO-PWY) | 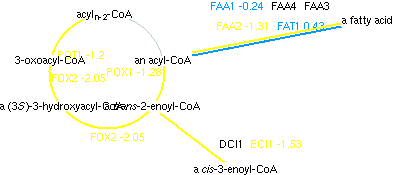 | | [d3,d2-Enoyl-CoA Isomerase](http://pathway.yeastgenome.org/YEAST/NEW-IMAGE?type=ENZYME&object=YLR284C-MONOMER) | [ECI1](http://db.yeastgenome.org/cgi-bin/locus.pl?locus=S000004274) | | --- | --- | | [delta(3,5)-delta(2,4)-dienoyl-CoA isomerase](http://pathway.yeastgenome.org/YEAST/NEW-IMAGE?type=ENZYME&object=YOR180C-MONOMER) | [DCI1](http://db.yeastgenome.org/cgi-bin/locus.pl?locus=S000005706) | | [fatty acid transporter](http://pathway.yeastgenome.org/YEAST/NEW-IMAGE?type=ENZYME&object=YBR041W-MONOMER) | [FAT1](http://db.yeastgenome.org/cgi-bin/locus.pl?locus=S000000245) | | [acyl-CoA synthetase](http://pathway.yeastgenome.org/YEAST/NEW-IMAGE?type=ENZYME&object=YER015W-MONOMER) | [FAA2](http://db.yeastgenome.org/cgi-bin/locus.pl?locus=S000000817) | | [acyl-CoA synthase](http://pathway.yeastgenome.org/YEAST/NEW-IMAGE?type=ENZYME&object=YIL009W-MONOMER) | [FAA3](http://db.yeastgenome.org/cgi-bin/locus.pl?locus=S000001271) | | [long chain fatty acyl:CoA synthetase](http://pathway.yeastgenome.org/YEAST/NEW-IMAGE?type=ENZYME&object=YMR246W-MONOMER) | [FAA4](http://db.yeastgenome.org/cgi-bin/locus.pl?locus=S000004860) | | [long chain fatty acyl:CoA synthetase](http://pathway.yeastgenome.org/YEAST/NEW-IMAGE?type=ENZYME&object=YOR317W-MONOMER) | [FAA1](http://db.yeastgenome.org/cgi-bin/locus.pl?locus=S000005844) | | [fatty-acyl coenzyme A oxidase](http://pathway.yeastgenome.org/YEAST/NEW-IMAGE?type=ENZYME&object=YGL205W-MONOMER) | [POX1](http://db.yeastgenome.org/cgi-bin/locus.pl?locus=S000003173) | | [3-hydroxyacyl-CoA dehydrogenase](http://pathway.yeastgenome.org/YEAST/NEW-IMAGE?type=ENZYME&object=YKR009C-MONOMER) | [FOX2](http://db.yeastgenome.org/cgi-bin/locus.pl?locus=S000001717) | | [3-oxoacyl CoA thiolase](http://pathway.yeastgenome.org/YEAST/NEW-IMAGE?type=ENZYME&object=YIL160C-MONOMER) | [POT1](http://db.yeastgenome.org/cgi-bin/locus.pl?locus=S000001422) | |
| 72 | [formaldehyde oxidation II (glutathione-dependent)](http://pathway.yeastgenome.org/YEAST/NEW-IMAGE?type=PATHWAY&object=PWY-1801) | 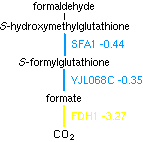 | | [formaldehyde dehydrogenase / alcohol dehydrogenase](http://pathway.yeastgenome.org/YEAST/NEW-IMAGE?type=ENZYME&object=YDL168W-MONOMER) | [SFA1](http://db.yeastgenome.org/cgi-bin/locus.pl?locus=S000002327) | | --- | --- | | [S-formylglutathione hydrolase](http://pathway.yeastgenome.org/YEAST/NEW-IMAGE?type=ENZYME&object=YJL068C-MONOMER) | [YJL068C](http://db.yeastgenome.org/cgi-bin/locus.pl?locus=S000003604) | | [formate dehydrogenases](http://pathway.yeastgenome.org/YEAST/NEW-IMAGE?type=ENZYME&object=YOR388C-MONOMER) | [FDH1](http://db.yeastgenome.org/cgi-bin/locus.pl?locus=S000005915) | |
| 76 | [leucine degradation](http://pathway.yeastgenome.org/YEAST/NEW-IMAGE?type=PATHWAY&object=PWY3O-4112) | 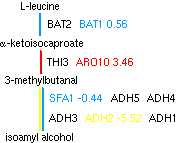 | | [branched-chain amino acid aminotransferase](http://pathway.yeastgenome.org/YEAST/NEW-IMAGE?type=ENZYME&object=YHR208W-MONOMER) | [BAT1](http://db.yeastgenome.org/cgi-bin/locus.pl?locus=S000001251) | | --- | --- | | [branched-chain amino acid transaminase](http://pathway.yeastgenome.org/YEAST/NEW-IMAGE?type=ENZYME&object=YJR148W-MONOMER) | [BAT2](http://db.yeastgenome.org/cgi-bin/locus.pl?locus=S000003909) | | [decarboxylase](http://pathway.yeastgenome.org/YEAST/NEW-IMAGE?type=ENZYME&object=CPLX3O-71) | [ARO10](http://db.yeastgenome.org/cgi-bin/locus.pl?locus=S000002788) | | [ketoisocaproate decarboxylase / decarboxylase](http://pathway.yeastgenome.org/YEAST/NEW-IMAGE?type=ENZYME&object=CPLX3O-110) | [THI3](http://db.yeastgenome.org/cgi-bin/locus.pl?locus=S000002238) | | [alcohol dehydrogenase](http://pathway.yeastgenome.org/YEAST/NEW-IMAGE?type=ENZYME&object=YOL086C-MONOMER) | [ADH1](http://db.yeastgenome.org/cgi-bin/locus.pl?locus=S000005446) | | [alcohol dehydrogenase](http://pathway.yeastgenome.org/YEAST/NEW-IMAGE?type=ENZYME&object=YMR303C-MONOMER) | [ADH2](http://db.yeastgenome.org/cgi-bin/locus.pl?locus=S000004918) | | [alcohol dehydrogenase](http://pathway.yeastgenome.org/YEAST/NEW-IMAGE?type=ENZYME&object=YMR083W-MONOMER) | [ADH3](http://db.yeastgenome.org/cgi-bin/locus.pl?locus=S000004688) | | [alcohol dehydrogenase](http://pathway.yeastgenome.org/YEAST/NEW-IMAGE?type=ENZYME&object=YGL256W-MONOMER) | [ADH4](http://db.yeastgenome.org/cgi-bin/locus.pl?locus=S000003225) | | [alcohol dehydrogenase](http://pathway.yeastgenome.org/YEAST/NEW-IMAGE?type=ENZYME&object=YBR145W-MONOMER) | [ADH5](http://db.yeastgenome.org/cgi-bin/locus.pl?locus=S000000349) | | [formaldehyde dehydrogenase / alcohol dehydrogenase](http://pathway.yeastgenome.org/YEAST/NEW-IMAGE?type=ENZYME&object=YDL168W-MONOMER) | [SFA1](http://db.yeastgenome.org/cgi-bin/locus.pl?locus=S000002327) | |
| 77 | [threonine degradation](http://pathway.yeastgenome.org/YEAST/NEW-IMAGE?type=PATHWAY&object=THREOCAT2-PWY) | 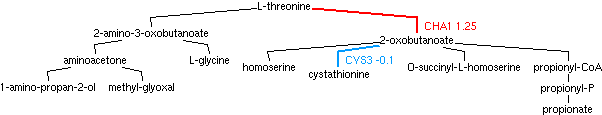 | | [serine/threonine dehydratase](http://pathway.yeastgenome.org/YEAST/NEW-IMAGE?type=ENZYME&object=YCL064C-MONOMER) | [CHA1](http://db.yeastgenome.org/cgi-bin/locus.pl?locus=S000000569) | | --- | --- | | [cystathionine gamma-lyase](http://pathway.yeastgenome.org/YEAST/NEW-IMAGE?type=ENZYME&object=YAL012W-MONOMER) | [CYS3](http://db.yeastgenome.org/cgi-bin/locus.pl?locus=S000000010) | |
| 78 | [valine degradation](http://pathway.yeastgenome.org/YEAST/NEW-IMAGE?type=PATHWAY&object=PWY3O-4105) | 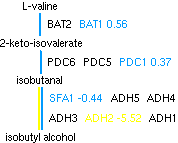 | | [branched-chain amino acid aminotransferase](http://pathway.yeastgenome.org/YEAST/NEW-IMAGE?type=ENZYME&object=YHR208W-MONOMER) | [BAT1](http://db.yeastgenome.org/cgi-bin/locus.pl?locus=S000001251) | | --- | --- | | [branched-chain amino acid transaminase](http://pathway.yeastgenome.org/YEAST/NEW-IMAGE?type=ENZYME&object=YJR148W-MONOMER) | [BAT2](http://db.yeastgenome.org/cgi-bin/locus.pl?locus=S000003909) | | [pyruvate decarboxylase / decarboxylase](http://pathway.yeastgenome.org/YEAST/NEW-IMAGE?type=ENZYME&object=CPLX3O-118) | [PDC1](http://db.yeastgenome.org/cgi-bin/locus.pl?locus=S000004034) | | [pyruvate decarboxylase / decarboxylase](http://pathway.yeastgenome.org/YEAST/NEW-IMAGE?type=ENZYME&object=CPLX3O-67) | [PDC5](http://db.yeastgenome.org/cgi-bin/locus.pl?locus=S000004124) | | [pyruvate decarboxylase / decarboxylase](http://pathway.yeastgenome.org/YEAST/NEW-IMAGE?type=ENZYME&object=CPLX3O-58) | [PDC6](http://db.yeastgenome.org/cgi-bin/locus.pl?locus=S000003319) | | [alcohol dehydrogenase](http://pathway.yeastgenome.org/YEAST/NEW-IMAGE?type=ENZYME&object=YOL086C-MONOMER) | [ADH1](http://db.yeastgenome.org/cgi-bin/locus.pl?locus=S000005446) | | [alcohol dehydrogenase](http://pathway.yeastgenome.org/YEAST/NEW-IMAGE?type=ENZYME&object=YMR303C-MONOMER) | [ADH2](http://db.yeastgenome.org/cgi-bin/locus.pl?locus=S000004918) | | [alcohol dehydrogenase](http://pathway.yeastgenome.org/YEAST/NEW-IMAGE?type=ENZYME&object=YMR083W-MONOMER) | [ADH3](http://db.yeastgenome.org/cgi-bin/locus.pl?locus=S000004688) | | [alcohol dehydrogenase](http://pathway.yeastgenome.org/YEAST/NEW-IMAGE?type=ENZYME&object=YGL256W-MONOMER) | [ADH4](http://db.yeastgenome.org/cgi-bin/locus.pl?locus=S000003225) | | [alcohol dehydrogenase](http://pathway.yeastgenome.org/YEAST/NEW-IMAGE?type=ENZYME&object=YBR145W-MONOMER) | [ADH5](http://db.yeastgenome.org/cgi-bin/locus.pl?locus=S000000349) | | [formaldehyde dehydrogenase / alcohol dehydrogenase](http://pathway.yeastgenome.org/YEAST/NEW-IMAGE?type=ENZYME&object=YDL168W-MONOMER) | [SFA1](http://db.yeastgenome.org/cgi-bin/locus.pl?locus=S000002327) | |
| 79 | [isoleucine degradation](http://pathway.yeastgenome.org/YEAST/NEW-IMAGE?type=PATHWAY&object=PWY3O-4109) | 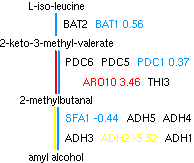 | | [branched-chain amino acid aminotransferase](http://pathway.yeastgenome.org/YEAST/NEW-IMAGE?type=ENZYME&object=YHR208W-MONOMER) | [BAT1](http://db.yeastgenome.org/cgi-bin/locus.pl?locus=S000001251) | | --- | --- | | [branched-chain amino acid transaminase](http://pathway.yeastgenome.org/YEAST/NEW-IMAGE?type=ENZYME&object=YJR148W-MONOMER) | [BAT2](http://db.yeastgenome.org/cgi-bin/locus.pl?locus=S000003909) | | [ketoisocaproate decarboxylase / decarboxylase](http://pathway.yeastgenome.org/YEAST/NEW-IMAGE?type=ENZYME&object=CPLX3O-110) | [THI3](http://db.yeastgenome.org/cgi-bin/locus.pl?locus=S000002238) | | [decarboxylase](http://pathway.yeastgenome.org/YEAST/NEW-IMAGE?type=ENZYME&object=CPLX3O-71) | [ARO10](http://db.yeastgenome.org/cgi-bin/locus.pl?locus=S000002788) | | [pyruvate decarboxylase / decarboxylase](http://pathway.yeastgenome.org/YEAST/NEW-IMAGE?type=ENZYME&object=CPLX3O-118) | [PDC1](http://db.yeastgenome.org/cgi-bin/locus.pl?locus=S000004034) | | [pyruvate decarboxylase / decarboxylase](http://pathway.yeastgenome.org/YEAST/NEW-IMAGE?type=ENZYME&object=CPLX3O-67) | [PDC5](http://db.yeastgenome.org/cgi-bin/locus.pl?locus=S000004124) | | [pyruvate decarboxylase / decarboxylase](http://pathway.yeastgenome.org/YEAST/NEW-IMAGE?type=ENZYME&object=CPLX3O-58) | [PDC6](http://db.yeastgenome.org/cgi-bin/locus.pl?locus=S000003319) | | [alcohol dehydrogenase](http://pathway.yeastgenome.org/YEAST/NEW-IMAGE?type=ENZYME&object=YOL086C-MONOMER) | [ADH1](http://db.yeastgenome.org/cgi-bin/locus.pl?locus=S000005446) | | [alcohol dehydrogenase](http://pathway.yeastgenome.org/YEAST/NEW-IMAGE?type=ENZYME&object=YMR303C-MONOMER) | [ADH2](http://db.yeastgenome.org/cgi-bin/locus.pl?locus=S000004918) | | [alcohol dehydrogenase](http://pathway.yeastgenome.org/YEAST/NEW-IMAGE?type=ENZYME&object=YMR083W-MONOMER) | [ADH3](http://db.yeastgenome.org/cgi-bin/locus.pl?locus=S000004688) | | [alcohol dehydrogenase](http://pathway.yeastgenome.org/YEAST/NEW-IMAGE?type=ENZYME&object=YGL256W-MONOMER) | [ADH4](http://db.yeastgenome.org/cgi-bin/locus.pl?locus=S000003225) | | [alcohol dehydrogenase](http://pathway.yeastgenome.org/YEAST/NEW-IMAGE?type=ENZYME&object=YBR145W-MONOMER) | [ADH5](http://db.yeastgenome.org/cgi-bin/locus.pl?locus=S000000349) | | [formaldehyde dehydrogenase / alcohol dehydrogenase](http://pathway.yeastgenome.org/YEAST/NEW-IMAGE?type=ENZYME&object=YDL168W-MONOMER) | [SFA1](http://db.yeastgenome.org/cgi-bin/locus.pl?locus=S000002327) | |
| 81 | [tryptophan degradation](http://pathway.yeastgenome.org/YEAST/NEW-IMAGE?type=PATHWAY&object=PWY3O-214) | 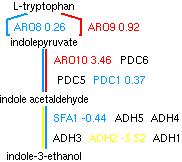 | | [aromatic amino acid aminotransferase II](http://pathway.yeastgenome.org/YEAST/NEW-IMAGE?type=ENZYME&object=YHR137W-MONOMER) | [ARO9](http://db.yeastgenome.org/cgi-bin/locus.pl?locus=S000001179) | | --- | --- | | [aromatic amino acid aminotransferase I](http://pathway.yeastgenome.org/YEAST/NEW-IMAGE?type=ENZYME&object=YGL202W-MONOMER) | [ARO8](http://db.yeastgenome.org/cgi-bin/locus.pl?locus=S000003170) | | [pyruvate decarboxylase / decarboxylase](http://pathway.yeastgenome.org/YEAST/NEW-IMAGE?type=ENZYME&object=CPLX3O-118) | [PDC1](http://db.yeastgenome.org/cgi-bin/locus.pl?locus=S000004034) | | [pyruvate decarboxylase / decarboxylase](http://pathway.yeastgenome.org/YEAST/NEW-IMAGE?type=ENZYME&object=CPLX3O-67) | [PDC5](http://db.yeastgenome.org/cgi-bin/locus.pl?locus=S000004124) | | [pyruvate decarboxylase / decarboxylase](http://pathway.yeastgenome.org/YEAST/NEW-IMAGE?type=ENZYME&object=CPLX3O-58) | [PDC6](http://db.yeastgenome.org/cgi-bin/locus.pl?locus=S000003319) | | [decarboxylase](http://pathway.yeastgenome.org/YEAST/NEW-IMAGE?type=ENZYME&object=CPLX3O-71) | [ARO10](http://db.yeastgenome.org/cgi-bin/locus.pl?locus=S000002788) | | [alcohol dehydrogenase](http://pathway.yeastgenome.org/YEAST/NEW-IMAGE?type=ENZYME&object=YOL086C-MONOMER) | [ADH1](http://db.yeastgenome.org/cgi-bin/locus.pl?locus=S000005446) | | [alcohol dehydrogenase](http://pathway.yeastgenome.org/YEAST/NEW-IMAGE?type=ENZYME&object=YMR303C-MONOMER) | [ADH2](http://db.yeastgenome.org/cgi-bin/locus.pl?locus=S000004918) | | [alcohol dehydrogenase](http://pathway.yeastgenome.org/YEAST/NEW-IMAGE?type=ENZYME&object=YMR083W-MONOMER) | [ADH3](http://db.yeastgenome.org/cgi-bin/locus.pl?locus=S000004688) | | [alcohol dehydrogenase](http://pathway.yeastgenome.org/YEAST/NEW-IMAGE?type=ENZYME&object=YGL256W-MONOMER) | [ADH4](http://db.yeastgenome.org/cgi-bin/locus.pl?locus=S000003225) | | [alcohol dehydrogenase](http://pathway.yeastgenome.org/YEAST/NEW-IMAGE?type=ENZYME&object=YBR145W-MONOMER) | [ADH5](http://db.yeastgenome.org/cgi-bin/locus.pl?locus=S000000349) | | [formaldehyde dehydrogenase / alcohol dehydrogenase](http://pathway.yeastgenome.org/YEAST/NEW-IMAGE?type=ENZYME&object=YDL168W-MONOMER) | [SFA1](http://db.yeastgenome.org/cgi-bin/locus.pl?locus=S000002327) | |
| 82 | [phenylalanine degradation](http://pathway.yeastgenome.org/YEAST/NEW-IMAGE?type=PATHWAY&object=PWY3O-4115) | 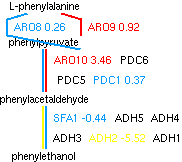 | | [aromatic amino acid aminotransferase II](http://pathway.yeastgenome.org/YEAST/NEW-IMAGE?type=ENZYME&object=YHR137W-MONOMER) | [ARO9](http://db.yeastgenome.org/cgi-bin/locus.pl?locus=S000001179) | | --- | --- | | [aromatic amino acid aminotransferase I](http://pathway.yeastgenome.org/YEAST/NEW-IMAGE?type=ENZYME&object=YGL202W-MONOMER) | [ARO8](http://db.yeastgenome.org/cgi-bin/locus.pl?locus=S000003170) | | [pyruvate decarboxylase / decarboxylase](http://pathway.yeastgenome.org/YEAST/NEW-IMAGE?type=ENZYME&object=CPLX3O-118) | [PDC1](http://db.yeastgenome.org/cgi-bin/locus.pl?locus=S000004034) | | [pyruvate decarboxylase / decarboxylase](http://pathway.yeastgenome.org/YEAST/NEW-IMAGE?type=ENZYME&object=CPLX3O-67) | [PDC5](http://db.yeastgenome.org/cgi-bin/locus.pl?locus=S000004124) | | [pyruvate decarboxylase / decarboxylase](http://pathway.yeastgenome.org/YEAST/NEW-IMAGE?type=ENZYME&object=CPLX3O-58) | [PDC6](http://db.yeastgenome.org/cgi-bin/locus.pl?locus=S000003319) | | [decarboxylase](http://pathway.yeastgenome.org/YEAST/NEW-IMAGE?type=ENZYME&object=CPLX3O-71) | [ARO10](http://db.yeastgenome.org/cgi-bin/locus.pl?locus=S000002788) | | [alcohol dehydrogenase](http://pathway.yeastgenome.org/YEAST/NEW-IMAGE?type=ENZYME&object=YOL086C-MONOMER) | [ADH1](http://db.yeastgenome.org/cgi-bin/locus.pl?locus=S000005446) | | [alcohol dehydrogenase](http://pathway.yeastgenome.org/YEAST/NEW-IMAGE?type=ENZYME&object=YMR303C-MONOMER) | [ADH2](http://db.yeastgenome.org/cgi-bin/locus.pl?locus=S000004918) | | [alcohol dehydrogenase](http://pathway.yeastgenome.org/YEAST/NEW-IMAGE?type=ENZYME&object=YMR083W-MONOMER) | [ADH3](http://db.yeastgenome.org/cgi-bin/locus.pl?locus=S000004688) | | [alcohol dehydrogenase](http://pathway.yeastgenome.org/YEAST/NEW-IMAGE?type=ENZYME&object=YGL256W-MONOMER) | [ADH4](http://db.yeastgenome.org/cgi-bin/locus.pl?locus=S000003225) | | [alcohol dehydrogenase](http://pathway.yeastgenome.org/YEAST/NEW-IMAGE?type=ENZYME&object=YBR145W-MONOMER) | [ADH5](http://db.yeastgenome.org/cgi-bin/locus.pl?locus=S000000349) | | [formaldehyde dehydrogenase / alcohol dehydrogenase](http://pathway.yeastgenome.org/YEAST/NEW-IMAGE?type=ENZYME&object=YDL168W-MONOMER) | [SFA1](http://db.yeastgenome.org/cgi-bin/locus.pl?locus=S000002327) | |
| 83 | [tyrosine degradation](http://pathway.yeastgenome.org/YEAST/NEW-IMAGE?type=PATHWAY&object=ASPARAGINE-DEG2-PWY) | 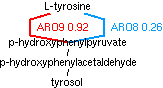 | | [aromatic amino acid aminotransferase II](http://pathway.yeastgenome.org/YEAST/NEW-IMAGE?type=ENZYME&object=YHR137W-MONOMER) | [ARO9](http://db.yeastgenome.org/cgi-bin/locus.pl?locus=S000001179) | | --- | --- | | [aromatic amino acid aminotransferase I](http://pathway.yeastgenome.org/YEAST/NEW-IMAGE?type=ENZYME&object=YGL202W-MONOMER) | [ARO8](http://db.yeastgenome.org/cgi-bin/locus.pl?locus=S000003170) | |
| 85 | [glycine](http://pathway.yeastgenome.org/YEAST/NEW-IMAGE?type=PATHWAY&object=ALANINE-DEG3-PWY) cleavage complex | 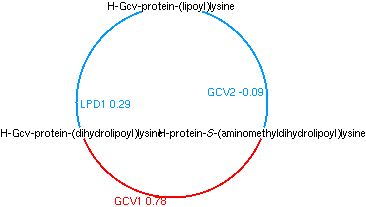 | | [glycine decarboxylase complex P subunit](http://pathway.yeastgenome.org/YEAST/NEW-IMAGE?type=ENZYME&object=YMR189W-MONOMER) | [GCV2](http://db.yeastgenome.org/cgi-bin/locus.pl?locus=S000004801) | | --- | --- | | [GCV1](http://pathway.yeastgenome.org/YEAST/NEW-IMAGE?type=ENZYME&object=YDR019C-MONOMER) | [GCV1](http://db.yeastgenome.org/cgi-bin/locus.pl?locus=S000002426) | | [dihydrolipoamide dehydrogenase](http://pathway.yeastgenome.org/YEAST/NEW-IMAGE?type=ENZYME&object=YFL018C-MONOMER) | [LPD1](http://db.yeastgenome.org/cgi-bin/locus.pl?locus=S000001876) | |
| 87 | [L-serine](http://pathway.yeastgenome.org/YEAST/NEW-IMAGE?type=PATHWAY&object=ALANINE-DEG3-PWY) degradation | 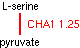 | | [serine/threonine dehydratase](http://pathway.yeastgenome.org/YEAST/NEW-IMAGE?type=ENZYME&object=YCL064C-MONOMER) | [CHA1](http://db.yeastgenome.org/cgi-bin/locus.pl?locus=S000000569) | | --- | --- | |
| 88 | [glutamate](http://pathway.yeastgenome.org/YEAST/NEW-IMAGE?type=PATHWAY&object=ALANINE-DEG3-PWY) degradation IX | 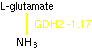 | | [NAD-dependent glutamate dehydrogenase](http://pathway.yeastgenome.org/YEAST/NEW-IMAGE?type=ENZYME&object=YDL215C-MONOMER) | [GDH2](http://db.yeastgenome.org/cgi-bin/locus.pl?locus=S000002374) | | --- | --- | |
| 93 | [glycogen catabolism](http://pathway.yeastgenome.org/YEAST/NEW-IMAGE?type=PATHWAY&object=GLYCOCAT-YEAST-PWY) | 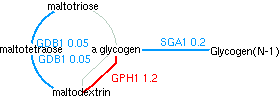 | | [glucoamylase](http://pathway.yeastgenome.org/YEAST/NEW-IMAGE?type=ENZYME&object=YIL099W-MONOMER) | [SGA1](http://db.yeastgenome.org/cgi-bin/locus.pl?locus=S000001361) | | --- | --- | | [glycogen phosphorylase](http://pathway.yeastgenome.org/YEAST/NEW-IMAGE?type=ENZYME&object=YPR160W-MONOMER) | [GPH1](http://db.yeastgenome.org/cgi-bin/locus.pl?locus=S000006364) | | [glucanotranferase](http://pathway.yeastgenome.org/YEAST/NEW-IMAGE?type=ENZYME&object=YPR184W-MONOMER) | [GDB1](http://db.yeastgenome.org/cgi-bin/locus.pl?locus=S000006388) | |
| 97 | [trehalose](http://pathway.yeastgenome.org/YEAST/NEW-IMAGE?type=PATHWAY&object=PYRUVDEHYD-PWY) degradation | 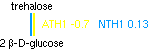 | | [neutral trehalase](http://pathway.yeastgenome.org/YEAST/NEW-IMAGE?type=ENZYME&object=YDR001C-MONOMER) | [NTH1](http://db.yeastgenome.org/cgi-bin/locus.pl?locus=S000002408) | | --- | --- | | [acid trehalase](http://pathway.yeastgenome.org/YEAST/NEW-IMAGE?type=ENZYME&object=YPR026W-MONOMER) | [ATH1](http://db.yeastgenome.org/cgi-bin/locus.pl?locus=S000006230) | |
| 100 | [allantoin degradation](http://pathway.yeastgenome.org/YEAST/NEW-IMAGE?type=PATHWAY&object=ALLANTOINDEG-PWY) | 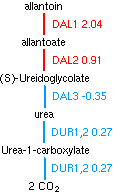 | | [allantoinase](http://pathway.yeastgenome.org/YEAST/NEW-IMAGE?type=ENZYME&object=YIR027C-MONOMER) | [DAL1](http://db.yeastgenome.org/cgi-bin/locus.pl?locus=S000001466) | | --- | --- | | [allantoicase](http://pathway.yeastgenome.org/YEAST/NEW-IMAGE?type=ENZYME&object=YIR029W-MONOMER) | [DAL2](http://db.yeastgenome.org/cgi-bin/locus.pl?locus=S000001468) | | [ureidoglycolate hydrolase](http://pathway.yeastgenome.org/YEAST/NEW-IMAGE?type=ENZYME&object=YIR032C-MONOMER) | [DAL3](http://db.yeastgenome.org/cgi-bin/locus.pl?locus=S000001471) | | [urea carboxylase / allophanate hydrolase](http://pathway.yeastgenome.org/YEAST/NEW-IMAGE?type=ENZYME&object=YBR208C-MONOMER) | [DUR1,2](http://db.yeastgenome.org/cgi-bin/locus.pl?locus=S000000412) | |
|  | phenylalanine biosynthesis | 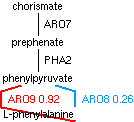 | | [chorismate mutase](http://pathway.yeastgenome.org/YEAST/NEW-IMAGE?type=ENZYME&object=YPR060C-MONOMER) | [ARO7](http://db.yeastgenome.org/cgi-bin/locus.pl?locus=S000006264) | | --- | --- | | [prephenate dehydratase](http://pathway.yeastgenome.org/YEAST/NEW-IMAGE?type=ENZYME&object=MONOMER3O-279) | [PHA2](http://db.yeastgenome.org/cgi-bin/locus.pl?locus=S000005260) | | [aromatic amino acid aminotransferase II](http://pathway.yeastgenome.org/YEAST/NEW-IMAGE?type=ENZYME&object=YHR137W-MONOMER) | [ARO9](http://db.yeastgenome.org/cgi-bin/locus.pl?locus=S000001179) | | [aromatic amino acid aminotransferase I](http://pathway.yeastgenome.org/YEAST/NEW-IMAGE?type=ENZYME&object=YGL202W-MONOMER) | [ARO8](http://db.yeastgenome.org/cgi-bin/locus.pl?locus=S000003170) | |
|  | tyrosine biosynthesis | 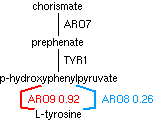 | | [chorismate mutase](http://pathway.yeastgenome.org/YEAST/NEW-IMAGE?type=ENZYME&object=YPR060C-MONOMER) | [ARO7](http://db.yeastgenome.org/cgi-bin/locus.pl?locus=S000006264) | | --- | --- | | [prephenate dehydrogenase (NADP+)](http://pathway.yeastgenome.org/YEAST/NEW-IMAGE?type=ENZYME&object=YBR166C-MONOMER) | [TYR1](http://db.yeastgenome.org/cgi-bin/locus.pl?locus=S000000370) | | [aromatic amino acid aminotransferase II](http://pathway.yeastgenome.org/YEAST/NEW-IMAGE?type=ENZYME&object=YHR137W-MONOMER) | [ARO9](http://db.yeastgenome.org/cgi-bin/locus.pl?locus=S000001179) | | [aromatic amino acid aminotransferase I](http://pathway.yeastgenome.org/YEAST/NEW-IMAGE?type=ENZYME&object=YGL202W-MONOMER) | [ARO8](http://db.yeastgenome.org/cgi-bin/locus.pl?locus=S000003170) | |
