# Supplementary material for: The impact of oxygen on the transcriptome of recombinant S. cerevisiae and P. pastoris - a comparative analysis
Source: BMC Genomics. 2011 May 9;12:218. doi: 10.1186/1471-2164-12-218 (PMC3116504; doi:10.1186/1471-2164-12-218)
Supplement: Additional file 3 — MetaCyc data Pichia pastoris. Regulated P. pastoris pathways in hypoxia vs. normoxia. Individual P. pastoris (recombinant strain) pathways that were transcriptionally regulated (i e. exceeding the log2 FC threshold of 0.59) in the comparison hypoxic vs. normoxic conditions, as resulting from the MetaCyc analysis presented in Figure 3 http://pathway.yeastgenome.org. Pathway numbers in the first column are referred to Figure 3. Pathway diagrams show all the intermediates of each pathways; reaction lines and the corresponding genes are colour-coded (three colour bins) according to the fold change threshold: red for upregulated, yellow for downregulated and blue for unregulated; log2 FC for each gene are also shown in colour. Last column contains the extended enzyme names corresponding to each gene of the pathway. [file 1471-2164-12-218-S3.DOC]

**Additional file 3 – Regulated *P. pastoris* pathways in hypoxia vs. normoxia**

Individual *P. pastoris* (Fab-producing strain) pathways transcriptionally regulated (*i e.* exceeding log2 FC threshold of 0.59) in the comparison hypoxic vs. normoxic conditions, as resulting from MetaCyc analysis (see Figure 3). Pathway numbers are referred to Figure 3 legend.

| **N^o^** | **Pathway** | **Pathway Diagram** | **Enzymes, Genes** |
| --- | --- | --- | --- |
| 2 | [aspartate biosynthesis](http://pathway.yeastgenome.org/YEAST/NEW-IMAGE?type=PATHWAY&object=ASPBIO-PWY) | 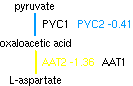 | | [pyruvate carboxylase](http://pathway.yeastgenome.org/YEAST/NEW-IMAGE?type=ENZYME&object=YBR218C-MONOMER) | [PYC2](http://db.yeastgenome.org/cgi-bin/locus.pl?locus=S000000422) | | --- | --- | | [pyruvate carboxylase](http://pathway.yeastgenome.org/YEAST/NEW-IMAGE?type=ENZYME&object=YGL062W-MONOMER) | [PYC1](http://db.yeastgenome.org/cgi-bin/locus.pl?locus=S000003030) | | [aspartate aminotransferase](http://pathway.yeastgenome.org/YEAST/NEW-IMAGE?type=ENZYME&object=YKL106W-MONOMER) | [AAT1](http://db.yeastgenome.org/cgi-bin/locus.pl?locus=S000001589) | | [aspartate aminotransferase](http://pathway.yeastgenome.org/YEAST/NEW-IMAGE?type=ENZYME&object=YLR027C-MONOMER) | [AAT2](http://db.yeastgenome.org/cgi-bin/locus.pl?locus=S000004017) | |
| 3 | [asparagine biosynthesis](http://pathway.yeastgenome.org/YEAST/NEW-IMAGE?type=PATHWAY&object=ASPARAGINE-BIOSYNTHESIS) | 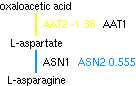 | | [aspartate aminotransferase](http://pathway.yeastgenome.org/YEAST/NEW-IMAGE?type=ENZYME&object=YKL106W-MONOMER) | [AAT1](http://db.yeastgenome.org/cgi-bin/locus.pl?locus=S000001589) | | --- | --- | | [aspartate aminotransferase](http://pathway.yeastgenome.org/YEAST/NEW-IMAGE?type=ENZYME&object=YLR027C-MONOMER) | [AAT2](http://db.yeastgenome.org/cgi-bin/locus.pl?locus=S000004017) | | [ASN2](http://pathway.yeastgenome.org/YEAST/NEW-IMAGE?type=ENZYME&object=YGR124W-MONOMER) | [ASN2](http://db.yeastgenome.org/cgi-bin/locus.pl?locus=S000003356) | | [ASN1](http://pathway.yeastgenome.org/YEAST/NEW-IMAGE?type=ENZYME&object=YPR145W-MONOMER) | [ASN1](http://db.yeastgenome.org/cgi-bin/locus.pl?locus=S000006349) | |
| 4 | [pantothenate and coenzyme A biosynthesis](http://pathway.yeastgenome.org/YEAST/NEW-IMAGE?type=PATHWAY&object=PANTOSYN2-PWY) | 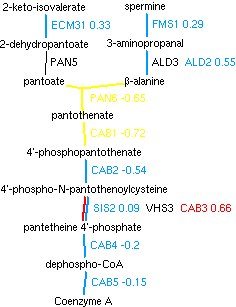 | | [amine oxidase](http://pathway.yeastgenome.org/YEAST/NEW-IMAGE?type=ENZYME&object=YMR020W-MONOMER) | [FMS1](http://db.yeastgenome.org/cgi-bin/locus.pl?locus=S000004622) | | --- | --- | | [aldehyde dehydrogenase (stress inducible cytoplasmic)](http://pathway.yeastgenome.org/YEAST/NEW-IMAGE?type=ENZYME&object=YMR170C-MONOMER) | [ALD2](http://db.yeastgenome.org/cgi-bin/locus.pl?locus=S000004780) | | [aldehyde dehydrogenase (stress inducible cytoplasmic)](http://pathway.yeastgenome.org/YEAST/NEW-IMAGE?type=ENZYME&object=YMR169C-MONOMER) | [ALD3](http://db.yeastgenome.org/cgi-bin/locus.pl?locus=S000004779) | | [3-methyl-2-oxobutanoate hydroxymethyltransferase](http://pathway.yeastgenome.org/YEAST/NEW-IMAGE?type=ENZYME&object=MONOMER3O-90) | [ECM31](http://db.yeastgenome.org/cgi-bin/locus.pl?locus=S000000380) | | [gluconate 5-dehydrogenase](http://pathway.yeastgenome.org/YEAST/NEW-IMAGE?type=ENZYME&object=MONOMER3O-214) | [PAN5](http://db.yeastgenome.org/cgi-bin/locus.pl?locus=S000001105) | | [pantoate-beta-alanine ligase](http://pathway.yeastgenome.org/YEAST/NEW-IMAGE?type=ENZYME&object=YIL145C-MONOMER) | [PAN6](http://db.yeastgenome.org/cgi-bin/locus.pl?locus=S000001407) | | [pantothenate kinase](http://pathway.yeastgenome.org/YEAST/NEW-IMAGE?type=ENZYME&object=MONOMER3O-268) | [CAB1](http://db.yeastgenome.org/cgi-bin/locus.pl?locus=S000002939) | | [phosphopantothenate-cysteine ligase](http://pathway.yeastgenome.org/YEAST/NEW-IMAGE?type=ENZYME&object=MONOMER3O-285) | [CAB2](http://db.yeastgenome.org/cgi-bin/locus.pl?locus=S000001345) | | [phosphopantothenoylcysteine decarboxylase](http://pathway.yeastgenome.org/YEAST/NEW-IMAGE?type=ENZYME&object=MONOMER3O-299) | [CAB3](http://db.yeastgenome.org/cgi-bin/locus.pl?locus=S000001571) | | [phosphopantothenoylcysteine decarboxylase](http://pathway.yeastgenome.org/YEAST/NEW-IMAGE?type=ENZYME&object=MONOMER3O-317) | [VHS3](http://db.yeastgenome.org/cgi-bin/locus.pl?locus=S000005580) | | [phosphopantothenoylcysteine decarboxylase](http://pathway.yeastgenome.org/YEAST/NEW-IMAGE?type=ENZYME&object=MONOMER3O-474) | [SIS2](http://db.yeastgenome.org/cgi-bin/locus.pl?locus=S000001780) | | [pantetheine-phosphate adenylyltransferase](http://pathway.yeastgenome.org/YEAST/NEW-IMAGE?type=ENZYME&object=MONOMER3O-487) | [CAB4](http://db.yeastgenome.org/cgi-bin/locus.pl?locus=S000003509) | | [dephospho-CoA kinase](http://pathway.yeastgenome.org/YEAST/NEW-IMAGE?type=ENZYME&object=MONOMER3O-504) | [CAB5](http://db.yeastgenome.org/cgi-bin/locus.pl?locus=S000002604) | |
| 8 | [arginine biosynthesis](http://pathway.yeastgenome.org/YEAST/NEW-IMAGE?type=PATHWAY&object=YEAST-ARG-SYN-PWY) | 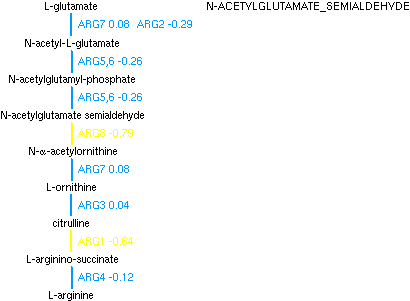 | | [acetylglutamate synthase](http://pathway.yeastgenome.org/YEAST/NEW-IMAGE?type=ENZYME&object=YJL071W-MONOMER) | [ARG2](http://db.yeastgenome.org/cgi-bin/locus.pl?locus=S000003607) | | --- | --- | | [acetylglutamate kinase / N-acetyl-gamma-glutamyl-phosphate reductase](http://pathway.yeastgenome.org/YEAST/NEW-IMAGE?type=ENZYME&object=YER069W-MONOMER) | [ARG5,6](http://db.yeastgenome.org/cgi-bin/locus.pl?locus=S000000871) | | [acetylornithine aminotransferase](http://pathway.yeastgenome.org/YEAST/NEW-IMAGE?type=ENZYME&object=YOL140W-MONOMER) | [ARG8](http://db.yeastgenome.org/cgi-bin/locus.pl?locus=S000005500) | | [acetylornithine acetyltransferase](http://pathway.yeastgenome.org/YEAST/NEW-IMAGE?type=ENZYME&object=YMR062C-MONOMER) | [ARG7](http://db.yeastgenome.org/cgi-bin/locus.pl?locus=S000004666) | | [ornithine carbamoyltransferase](http://pathway.yeastgenome.org/YEAST/NEW-IMAGE?type=ENZYME&object=YJL088W-MONOMER) | [ARG3](http://db.yeastgenome.org/cgi-bin/locus.pl?locus=S000003624) | | [arginosuccinate synthetase](http://pathway.yeastgenome.org/YEAST/NEW-IMAGE?type=ENZYME&object=YOL058W-MONOMER) | [ARG1](http://db.yeastgenome.org/cgi-bin/locus.pl?locus=S000005419) | | [argininosuccinate lyase](http://pathway.yeastgenome.org/YEAST/NEW-IMAGE?type=ENZYME&object=YHR018C-MONOMER) | [ARG4](http://db.yeastgenome.org/cgi-bin/locus.pl?locus=S000001060) | |
| 9 | [arginine degradation (anaerobic)](http://pathway.yeastgenome.org/YEAST/NEW-IMAGE?type=PATHWAY&object=ARG-PRO-PWY) | 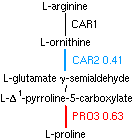 | | [arginase](http://pathway.yeastgenome.org/YEAST/NEW-IMAGE?type=ENZYME&object=YPL111W-MONOMER) | [CAR1](http://db.yeastgenome.org/cgi-bin/locus.pl?locus=S000006032) | | --- | --- | | [ornithine aminotransferase](http://pathway.yeastgenome.org/YEAST/NEW-IMAGE?type=ENZYME&object=YLR438W-MONOMER) | [CAR2](http://db.yeastgenome.org/cgi-bin/locus.pl?locus=S000004430) | | [delta 1-pyrroline-5-carboxylate reductase](http://pathway.yeastgenome.org/YEAST/NEW-IMAGE?type=ENZYME&object=YER023W-MONOMER) | [PRO3](http://db.yeastgenome.org/cgi-bin/locus.pl?locus=S000000825) | |
| 10 | [arginine biosynthesis](http://pathway.yeastgenome.org/YEAST/NEW-IMAGE?type=PATHWAY&object=ARGSYNBSUB-PWY) | 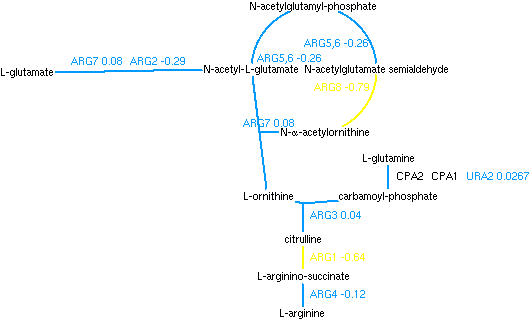 | | [acetylglutamate synthase](http://pathway.yeastgenome.org/YEAST/NEW-IMAGE?type=ENZYME&object=YJL071W-MONOMER) | [ARG2](http://db.yeastgenome.org/cgi-bin/locus.pl?locus=S000003607) | | --- | --- | | [acetylglutamate kinase / N-acetyl-gamma-glutamyl-phosphate reductase](http://pathway.yeastgenome.org/YEAST/NEW-IMAGE?type=ENZYME&object=YER069W-MONOMER) | [ARG5,6](http://db.yeastgenome.org/cgi-bin/locus.pl?locus=S000000871) | | [acetylornithine aminotransferase](http://pathway.yeastgenome.org/YEAST/NEW-IMAGE?type=ENZYME&object=YOL140W-MONOMER) | [ARG8](http://db.yeastgenome.org/cgi-bin/locus.pl?locus=S000005500) | | [acetylornithine acetyltransferase](http://pathway.yeastgenome.org/YEAST/NEW-IMAGE?type=ENZYME&object=YMR062C-MONOMER) | [ARG7](http://db.yeastgenome.org/cgi-bin/locus.pl?locus=S000004666) | | [carbamyl phosphate synthase / aspartate transcarbamylase](http://pathway.yeastgenome.org/YEAST/NEW-IMAGE?type=ENZYME&object=YJL130C-MONOMER) | [URA2](http://db.yeastgenome.org/cgi-bin/locus.pl?locus=S000003666) | | [carbamoyl phosphate synthetase](http://pathway.yeastgenome.org/YEAST/NEW-IMAGE?type=ENZYME&object=CPLX3O-887) | [CPA2](http://db.yeastgenome.org/cgi-bin/locus.pl?locus=S000003870) [CPA1](http://db.yeastgenome.org/cgi-bin/locus.pl?locus=S000005829) | | [ornithine carbamoyltransferase](http://pathway.yeastgenome.org/YEAST/NEW-IMAGE?type=ENZYME&object=YJL088W-MONOMER) | [ARG3](http://db.yeastgenome.org/cgi-bin/locus.pl?locus=S000003624) | | [arginosuccinate synthetase](http://pathway.yeastgenome.org/YEAST/NEW-IMAGE?type=ENZYME&object=YOL058W-MONOMER) | [ARG1](http://db.yeastgenome.org/cgi-bin/locus.pl?locus=S000005419) | | [argininosuccinate lyase](http://pathway.yeastgenome.org/YEAST/NEW-IMAGE?type=ENZYME&object=YHR018C-MONOMER) | [ARG4](http://db.yeastgenome.org/cgi-bin/locus.pl?locus=S000001060) | |
| 11 | [isoleucine biosynthesis](http://pathway.yeastgenome.org/YEAST/NEW-IMAGE?type=PATHWAY&object=ILEUSYN-PWY) | 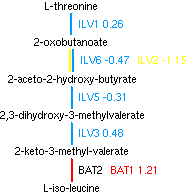 | | [threonine deaminase](http://pathway.yeastgenome.org/YEAST/NEW-IMAGE?type=ENZYME&object=YER086W-MONOMER) | [ILV1](http://db.yeastgenome.org/cgi-bin/locus.pl?locus=S000000888) | | --- | --- | | [acetolactate synthase](http://pathway.yeastgenome.org/YEAST/NEW-IMAGE?type=ENZYME&object=CPLX3O-30) | [ILV6](http://db.yeastgenome.org/cgi-bin/locus.pl?locus=S000000515) [ILV2](http://db.yeastgenome.org/cgi-bin/locus.pl?locus=S000004714) | | [acetohydroxyacid reductoisomerase](http://pathway.yeastgenome.org/YEAST/NEW-IMAGE?type=ENZYME&object=YLR355C-MONOMER) | [ILV5](http://db.yeastgenome.org/cgi-bin/locus.pl?locus=S000004347) | | [dihydroxy-acid dehydratase](http://pathway.yeastgenome.org/YEAST/NEW-IMAGE?type=ENZYME&object=YJR016C-MONOMER) | [ILV3](http://db.yeastgenome.org/cgi-bin/locus.pl?locus=S000003777) | | [branched-chain amino acid aminotransferase](http://pathway.yeastgenome.org/YEAST/NEW-IMAGE?type=ENZYME&object=YHR208W-MONOMER) | [BAT1](http://db.yeastgenome.org/cgi-bin/locus.pl?locus=S000001251) | | [branched-chain amino acid transaminase](http://pathway.yeastgenome.org/YEAST/NEW-IMAGE?type=ENZYME&object=YJR148W-MONOMER) | [BAT2](http://db.yeastgenome.org/cgi-bin/locus.pl?locus=S000003909) | |
| 11 | [valine biosynthesis](http://pathway.yeastgenome.org/YEAST/NEW-IMAGE?type=PATHWAY&object=VALSYN-PWY) | 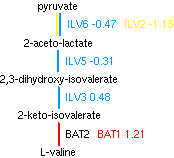 | | [acetolactate synthase](http://pathway.yeastgenome.org/YEAST/NEW-IMAGE?type=ENZYME&object=CPLX3O-30) | [ILV6](http://db.yeastgenome.org/cgi-bin/locus.pl?locus=S000000515) [ILV2](http://db.yeastgenome.org/cgi-bin/locus.pl?locus=S000004714) | | --- | --- | | [acetohydroxyacid reductoisomerase](http://pathway.yeastgenome.org/YEAST/NEW-IMAGE?type=ENZYME&object=YLR355C-MONOMER) | [ILV5](http://db.yeastgenome.org/cgi-bin/locus.pl?locus=S000004347) | | [dihydroxy-acid dehydratase](http://pathway.yeastgenome.org/YEAST/NEW-IMAGE?type=ENZYME&object=YJR016C-MONOMER) | [ILV3](http://db.yeastgenome.org/cgi-bin/locus.pl?locus=S000003777) | | [branched-chain amino acid aminotransferase](http://pathway.yeastgenome.org/YEAST/NEW-IMAGE?type=ENZYME&object=YHR208W-MONOMER) | [BAT1](http://db.yeastgenome.org/cgi-bin/locus.pl?locus=S000001251) | | [branched-chain amino acid transaminase](http://pathway.yeastgenome.org/YEAST/NEW-IMAGE?type=ENZYME&object=YJR148W-MONOMER) | [BAT2](http://db.yeastgenome.org/cgi-bin/locus.pl?locus=S000003909) | |
| 11 | [acetoin biosynthesis](http://pathway.yeastgenome.org/YEAST/NEW-IMAGE?type=PATHWAY&object=PWY3O-335) | 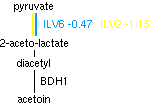 | | [acetolactate synthase](http://pathway.yeastgenome.org/YEAST/NEW-IMAGE?type=ENZYME&object=CPLX3O-30) | [ILV6](http://db.yeastgenome.org/cgi-bin/locus.pl?locus=S000000515) [ILV2](http://db.yeastgenome.org/cgi-bin/locus.pl?locus=S000004714) | | --- | --- | | [2,3-butanediol dehydrogenase / diacetyl reductase](http://pathway.yeastgenome.org/YEAST/NEW-IMAGE?type=ENZYME&object=CPLX3O-79) | [BDH1](http://db.yeastgenome.org/cgi-bin/locus.pl?locus=S000000056) | |
| 11 | [leucine biosynthesis](http://pathway.yeastgenome.org/YEAST/NEW-IMAGE?type=PATHWAY&object=LEUSYN-PWY) | 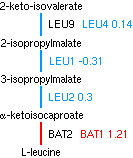 | | [alpha-isopropylmalate synthase](http://pathway.yeastgenome.org/YEAST/NEW-IMAGE?type=ENZYME&object=YNL104C-MONOMER) | [LEU4](http://db.yeastgenome.org/cgi-bin/locus.pl?locus=S000005048) | | --- | --- | | [alpha-isopropylmalate synthase, minor isozyme](http://pathway.yeastgenome.org/YEAST/NEW-IMAGE?type=ENZYME&object=MONOMER3O-59) | [LEU9](http://db.yeastgenome.org/cgi-bin/locus.pl?locus=S000005634) | | [isopropylmalate isomerase](http://pathway.yeastgenome.org/YEAST/NEW-IMAGE?type=ENZYME&object=YGL009C-MONOMER) | [LEU1](http://db.yeastgenome.org/cgi-bin/locus.pl?locus=S000002977) | | [beta-IPM dehydrogenase](http://pathway.yeastgenome.org/YEAST/NEW-IMAGE?type=ENZYME&object=YCL018W-MONOMER) | [LEU2](http://db.yeastgenome.org/cgi-bin/locus.pl?locus=S000000523) | | [branched-chain amino acid aminotransferase](http://pathway.yeastgenome.org/YEAST/NEW-IMAGE?type=ENZYME&object=YHR208W-MONOMER) | [BAT1](http://db.yeastgenome.org/cgi-bin/locus.pl?locus=S000001251) | | [branched-chain amino acid transaminase](http://pathway.yeastgenome.org/YEAST/NEW-IMAGE?type=ENZYME&object=YJR148W-MONOMER) | [BAT2](http://db.yeastgenome.org/cgi-bin/locus.pl?locus=S000003909) | |
| 12 | [serine biosynthesis from glyoxylate](http://pathway.yeastgenome.org/YEAST/NEW-IMAGE?type=PATHWAY&object=PWY3O-230) | 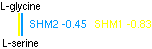 | | [Serine hydroxymethyltransferase, mitochondrial](http://pathway.yeastgenome.org/YEAST/NEW-IMAGE?type=ENZYME&object=YBR263W-MONOMER) | [SHM1](http://db.yeastgenome.org/cgi-bin/locus.pl?locus=S000000467) | | --- | --- | | [serine hydroxymethyltransferase](http://pathway.yeastgenome.org/YEAST/NEW-IMAGE?type=ENZYME&object=YLR058C-MONOMER) | [SHM2](http://db.yeastgenome.org/cgi-bin/locus.pl?locus=S000004048) | |
| 12 | [glycine biosynthesis from serine](http://pathway.yeastgenome.org/YEAST/NEW-IMAGE?type=PATHWAY&object=GLYSYN-SER-PWY) | 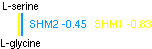 | | [Serine hydroxymethyltransferase, mitochondrial](http://pathway.yeastgenome.org/YEAST/NEW-IMAGE?type=ENZYME&object=YBR263W-MONOMER) | [SHM1](http://db.yeastgenome.org/cgi-bin/locus.pl?locus=S000000467) | | --- | --- | | [serine hydroxymethyltransferase](http://pathway.yeastgenome.org/YEAST/NEW-IMAGE?type=ENZYME&object=YLR058C-MONOMER) | [SHM2](http://db.yeastgenome.org/cgi-bin/locus.pl?locus=S000004048) | |
| 13 | [cysteine biosynthesis/ homocysteine degradation](http://pathway.yeastgenome.org/YEAST/NEW-IMAGE?type=PATHWAY&object=HOMOCYSDEGR-PWY) | 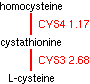 | | [cystathionine beta-synthase](http://pathway.yeastgenome.org/YEAST/NEW-IMAGE?type=ENZYME&object=YGR155W-MONOMER) | [CYS4](http://db.yeastgenome.org/cgi-bin/locus.pl?locus=S000003387) | | --- | --- | | [cystathionine gamma-lyase](http://pathway.yeastgenome.org/YEAST/NEW-IMAGE?type=ENZYME&object=YAL012W-MONOMER) | [CYS3](http://db.yeastgenome.org/cgi-bin/locus.pl?locus=S000000010) | |
| 13 | [sulfate assimilation pathway](http://pathway.yeastgenome.org/YEAST/NEW-IMAGE?type=PATHWAY&object=PWY-781) | 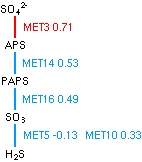 | | [ATP sulfurylase](http://pathway.yeastgenome.org/YEAST/NEW-IMAGE?type=ENZYME&object=YJR010W-MONOMER) | [MET3](http://db.yeastgenome.org/cgi-bin/locus.pl?locus=S000003771) | | --- | --- | | [adenylylsulfate kinase](http://pathway.yeastgenome.org/YEAST/NEW-IMAGE?type=ENZYME&object=YKL001C-MONOMER) | [MET14](http://db.yeastgenome.org/cgi-bin/locus.pl?locus=S000001484) | | [3'phosphoadenylylsulfate reductase](http://pathway.yeastgenome.org/YEAST/NEW-IMAGE?type=ENZYME&object=YPR167C-MONOMER) | [MET16](http://db.yeastgenome.org/cgi-bin/locus.pl?locus=S000006371) | | [sulfite reductase](http://pathway.yeastgenome.org/YEAST/NEW-IMAGE?type=ENZYME&object=CPLX3O-23) | [MET5](http://db.yeastgenome.org/cgi-bin/locus.pl?locus=S000003898) [MET10](http://db.yeastgenome.org/cgi-bin/locus.pl?locus=S000001926) | |
| 17 | [glutamate biosynthesis from ammonia](http://pathway.yeastgenome.org/YEAST/NEW-IMAGE?type=PATHWAY&object=GLUNH3-PWY) | 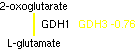 | | [NADP-dependent glutamate dehydrogenase](http://pathway.yeastgenome.org/YEAST/NEW-IMAGE?type=ENZYME&object=YAL062W-MONOMER) | [GDH3](http://db.yeastgenome.org/cgi-bin/locus.pl?locus=S000000058) | | --- | --- | | [NADP-dependent glutamate dehydrogenase](http://pathway.yeastgenome.org/YEAST/NEW-IMAGE?type=ENZYME&object=YOR375C-MONOMER) | [GDH1](http://db.yeastgenome.org/cgi-bin/locus.pl?locus=S000005902) | |
| 17 | [glutamate biosynthesis from glutamine](http://pathway.yeastgenome.org/YEAST/NEW-IMAGE?type=PATHWAY&object=GLUGLNSYN-PWY) | 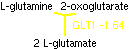 | | [glutamate synthase (NADH)](http://pathway.yeastgenome.org/YEAST/NEW-IMAGE?type=ENZYME&object=YDL171C-MONOMER) | [GLT1](http://db.yeastgenome.org/cgi-bin/locus.pl?locus=S000002330) | | --- | --- | |
| 17 | [superpathway of glutamate biosynthesis](http://pathway.yeastgenome.org/YEAST/NEW-IMAGE?type=PATHWAY&object=GLUTSYN-PWY) | 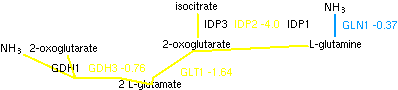 | | [NADP-dependent glutamate dehydrogenase](http://pathway.yeastgenome.org/YEAST/NEW-IMAGE?type=ENZYME&object=YAL062W-MONOMER) | [GDH3](http://db.yeastgenome.org/cgi-bin/locus.pl?locus=S000000058) | | --- | --- | | [NADP-dependent glutamate dehydrogenase](http://pathway.yeastgenome.org/YEAST/NEW-IMAGE?type=ENZYME&object=YOR375C-MONOMER) | [GDH1](http://db.yeastgenome.org/cgi-bin/locus.pl?locus=S000005902) | | [glutamine synthetase](http://pathway.yeastgenome.org/YEAST/NEW-IMAGE?type=ENZYME&object=YPR035W-MONOMER) | [GLN1](http://db.yeastgenome.org/cgi-bin/locus.pl?locus=S000006239) | | [NADP-dependent isocitrate dehydrogenase](http://pathway.yeastgenome.org/YEAST/NEW-IMAGE?type=ENZYME&object=YDL066W-MONOMER) | [IDP1](http://db.yeastgenome.org/cgi-bin/locus.pl?locus=S000002224) | | [NADP-dependent isocitrate dehydrogenase](http://pathway.yeastgenome.org/YEAST/NEW-IMAGE?type=ENZYME&object=YLR174W-MONOMER) | [IDP2](http://db.yeastgenome.org/cgi-bin/locus.pl?locus=S000004164) | | [NADP-dependent isocitrate dehydrogenase](http://pathway.yeastgenome.org/YEAST/NEW-IMAGE?type=ENZYME&object=YNL009W-MONOMER) | [IDP3](http://db.yeastgenome.org/cgi-bin/locus.pl?locus=S000004954) | | [glutamate synthase (NADH)](http://pathway.yeastgenome.org/YEAST/NEW-IMAGE?type=ENZYME&object=YDL171C-MONOMER) | [GLT1](http://db.yeastgenome.org/cgi-bin/locus.pl?locus=S000002330) | |
| 18 | [methionine salvage pathway](http://pathway.yeastgenome.org/YEAST/NEW-IMAGE?type=PATHWAY&object=PWY3O-64) | 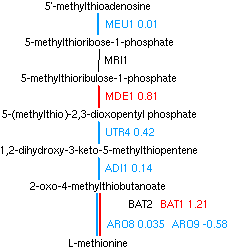 | | [5'-methylthioadenosine phosphorylase](http://pathway.yeastgenome.org/YEAST/NEW-IMAGE?type=ENZYME&object=MONOMER3O-157) | [MEU1](http://db.yeastgenome.org/cgi-bin/locus.pl?locus=S000004007) | | --- | --- | | [methylthioribose-1 P isomerase](http://pathway.yeastgenome.org/YEAST/NEW-IMAGE?type=ENZYME&object=CPLX3O-113) | [MRI1](http://db.yeastgenome.org/cgi-bin/locus.pl?locus=S000006322) | | [methylthioribulose-1-phosphate dehydratase](http://pathway.yeastgenome.org/YEAST/NEW-IMAGE?type=ENZYME&object=MONOMER3O-169) | [MDE1](http://db.yeastgenome.org/cgi-bin/locus.pl?locus=S000003785) | | [2,3-dioxomethiopentane-1-phosphate enolase/phosphatase](http://pathway.yeastgenome.org/YEAST/NEW-IMAGE?type=ENZYME&object=MONOMER3O-175) | [UTR4](http://db.yeastgenome.org/cgi-bin/locus.pl?locus=S000000764) | | [acireductone dioxygenase](http://pathway.yeastgenome.org/YEAST/NEW-IMAGE?type=ENZYME&object=MONOMER3O-186) | [ADI1](http://db.yeastgenome.org/cgi-bin/locus.pl?locus=S000004611) | | [aromatic amino acid aminotransferase II](http://pathway.yeastgenome.org/YEAST/NEW-IMAGE?type=ENZYME&object=YHR137W-MONOMER) | [ARO9](http://db.yeastgenome.org/cgi-bin/locus.pl?locus=S000001179) | | [aromatic amino acid aminotransferase I](http://pathway.yeastgenome.org/YEAST/NEW-IMAGE?type=ENZYME&object=YGL202W-MONOMER) | [ARO8](http://db.yeastgenome.org/cgi-bin/locus.pl?locus=S000003170) | | [branched-chain amino acid aminotransferase](http://pathway.yeastgenome.org/YEAST/NEW-IMAGE?type=ENZYME&object=YHR208W-MONOMER) | [BAT1](http://db.yeastgenome.org/cgi-bin/locus.pl?locus=S000001251) | | [branched-chain amino acid transaminase](http://pathway.yeastgenome.org/YEAST/NEW-IMAGE?type=ENZYME&object=YJR148W-MONOMER) | [BAT2](http://db.yeastgenome.org/cgi-bin/locus.pl?locus=S000003909) | |
| 19 | [proline biosynthesis](http://pathway.yeastgenome.org/YEAST/NEW-IMAGE?type=PATHWAY&object=PROSYN-PWY) | 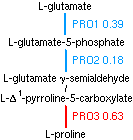 | | [gamma-glutamyl kinase](http://pathway.yeastgenome.org/YEAST/NEW-IMAGE?type=ENZYME&object=YDR300C-MONOMER) | [PRO1](http://db.yeastgenome.org/cgi-bin/locus.pl?locus=S000002708) | | --- | --- | | [gamma-glutamyl phosphate reductase](http://pathway.yeastgenome.org/YEAST/NEW-IMAGE?type=ENZYME&object=YOR323C-MONOMER) | [PRO2](http://db.yeastgenome.org/cgi-bin/locus.pl?locus=S000005850) | | [delta 1-pyrroline-5-carboxylate reductase](http://pathway.yeastgenome.org/YEAST/NEW-IMAGE?type=ENZYME&object=YER023W-MONOMER) | [PRO3](http://db.yeastgenome.org/cgi-bin/locus.pl?locus=S000000825) | |
| 20 | [ergosterol biosynthesis](http://pathway.yeastgenome.org/YEAST/NEW-IMAGE?type=PATHWAY&object=ERGOSTEROL-SYN-PWY) | 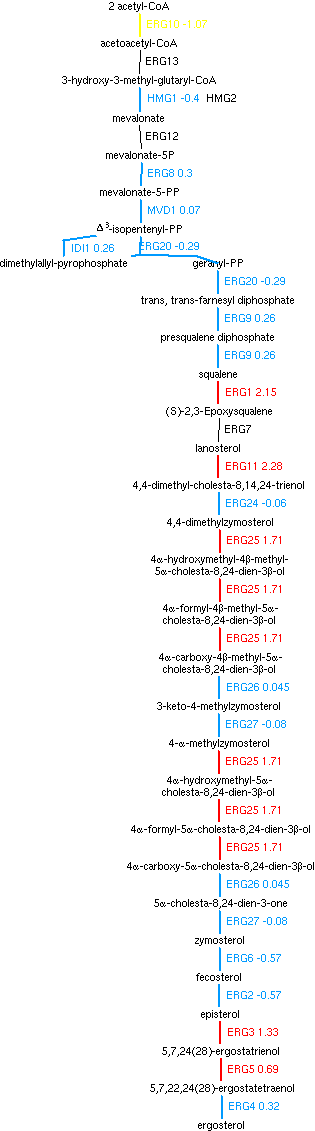  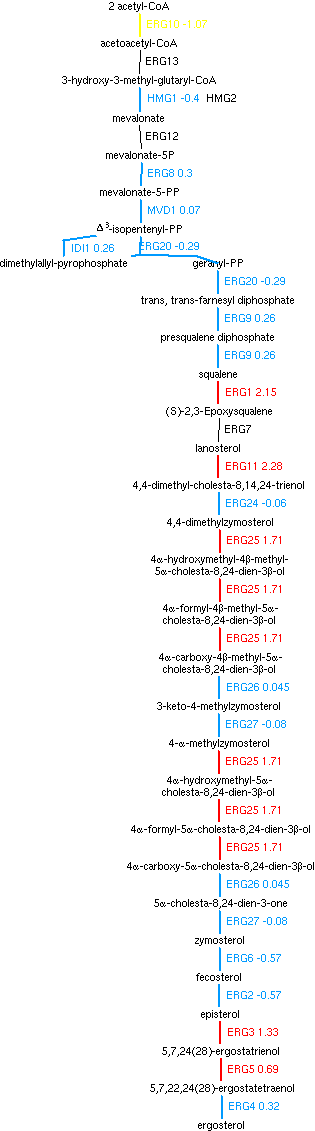 | | [acetoacetyl CoA thiolase](http://pathway.yeastgenome.org/YEAST/NEW-IMAGE?type=ENZYME&object=YPL028W-MONOMER) | [ERG10](http://db.yeastgenome.org/cgi-bin/locus.pl?locus=S000005949) | | --- | --- | | [3-hydroxy-3-methylglutaryl coenzyme A synthase](http://pathway.yeastgenome.org/YEAST/NEW-IMAGE?type=ENZYME&object=YML126C-MONOMER) | [ERG13](http://db.yeastgenome.org/cgi-bin/locus.pl?locus=S000004595) | | [3-hydroxy-3-methylglutaryl-coenzyme A (HMG-CoA)](http://pathway.yeastgenome.org/YEAST/NEW-IMAGE?type=ENZYME&object=YLR450W-MONOMER) | [HMG2](http://db.yeastgenome.org/cgi-bin/locus.pl?locus=S000004442) | | [3-hydroxy-3-methylglutaryl-coenzyme A (HMG-CoA)](http://pathway.yeastgenome.org/YEAST/NEW-IMAGE?type=ENZYME&object=YML075C-MONOMER) | [HMG1](http://db.yeastgenome.org/cgi-bin/locus.pl?locus=S000004540) | | [mevalonate kinase](http://pathway.yeastgenome.org/YEAST/NEW-IMAGE?type=ENZYME&object=YMR208W-MONOMER) | [ERG12](http://db.yeastgenome.org/cgi-bin/locus.pl?locus=S000004821) | | [phosphomevalonate kinase](http://pathway.yeastgenome.org/YEAST/NEW-IMAGE?type=ENZYME&object=YMR220W-MONOMER) | [ERG8](http://db.yeastgenome.org/cgi-bin/locus.pl?locus=S000004833) | | [mevalonate pyrophosphate decarboxylase](http://pathway.yeastgenome.org/YEAST/NEW-IMAGE?type=ENZYME&object=YNR043W-MONOMER) | [MVD1](http://db.yeastgenome.org/cgi-bin/locus.pl?locus=S000005326) | | [isopentenyl diphosphate:dimethylallyl diphosphate isomerase](http://pathway.yeastgenome.org/YEAST/NEW-IMAGE?type=ENZYME&object=YPL117C-MONOMER) | [IDI1](http://db.yeastgenome.org/cgi-bin/locus.pl?locus=S000006038) | | [farnesyl diphosphate synthetase](http://pathway.yeastgenome.org/YEAST/NEW-IMAGE?type=ENZYME&object=YJL167W-MONOMER) | [ERG20](http://db.yeastgenome.org/cgi-bin/locus.pl?locus=S000003703) | | [squalene synthetase](http://pathway.yeastgenome.org/YEAST/NEW-IMAGE?type=ENZYME&object=YHR190W-MONOMER) | [ERG9](http://db.yeastgenome.org/cgi-bin/locus.pl?locus=S000001233) | | [squalene monooxygenase](http://pathway.yeastgenome.org/YEAST/NEW-IMAGE?type=ENZYME&object=YGR175C-MONOMER) | [ERG1](http://db.yeastgenome.org/cgi-bin/locus.pl?locus=S000003407) | | [2,3-oxidosqualene-lanosterol cyclase](http://pathway.yeastgenome.org/YEAST/NEW-IMAGE?type=ENZYME&object=YHR072W-MONOMER) | [ERG7](http://db.yeastgenome.org/cgi-bin/locus.pl?locus=S000001114) | | [cytochrome P450 lanosterol 14a-demethylase](http://pathway.yeastgenome.org/YEAST/NEW-IMAGE?type=ENZYME&object=YHR007C-MONOMER) | [ERG11](http://db.yeastgenome.org/cgi-bin/locus.pl?locus=S000001049) | | [C-14 sterol reductase](http://pathway.yeastgenome.org/YEAST/NEW-IMAGE?type=ENZYME&object=YNL280C-MONOMER) | [ERG24](http://db.yeastgenome.org/cgi-bin/locus.pl?locus=S000005224) | | [C-4 sterol methyl oxidase](http://pathway.yeastgenome.org/YEAST/NEW-IMAGE?type=ENZYME&object=YGR060W-MONOMER) | [ERG25](http://db.yeastgenome.org/cgi-bin/locus.pl?locus=S000003292) | | [C-3 sterol dehydrogenase](http://pathway.yeastgenome.org/YEAST/NEW-IMAGE?type=ENZYME&object=YGL001C-MONOMER) | [ERG26](http://db.yeastgenome.org/cgi-bin/locus.pl?locus=S000002969) | | [3-keto sterol reductase](http://pathway.yeastgenome.org/YEAST/NEW-IMAGE?type=ENZYME&object=YLR100W-MONOMER) | [ERG27](http://db.yeastgenome.org/cgi-bin/locus.pl?locus=S000004090) | | [SAM:C-24 sterol methyltransferase](http://pathway.yeastgenome.org/YEAST/NEW-IMAGE?type=ENZYME&object=MONOMER3O-188) | [ERG6](http://db.yeastgenome.org/cgi-bin/locus.pl?locus=S000004467) | | [C-8 sterol isomerase](http://pathway.yeastgenome.org/YEAST/NEW-IMAGE?type=ENZYME&object=YMR202W-MONOMER) | [ERG2](http://db.yeastgenome.org/cgi-bin/locus.pl?locus=S000004815) | | [C-5 sterol desaturase](http://pathway.yeastgenome.org/YEAST/NEW-IMAGE?type=ENZYME&object=YLR056W-MONOMER) | [ERG3](http://db.yeastgenome.org/cgi-bin/locus.pl?locus=S000004046) | | [C-22 sterol desaturase](http://pathway.yeastgenome.org/YEAST/NEW-IMAGE?type=ENZYME&object=MONOMER3O-232) | [ERG5](http://db.yeastgenome.org/cgi-bin/locus.pl?locus=S000004617) | | [C-24 sterol reductase](http://pathway.yeastgenome.org/YEAST/NEW-IMAGE?type=ENZYME&object=YGL012W-MONOMER) | [ERG4](http://db.yeastgenome.org/cgi-bin/locus.pl?locus=S000002980) | |
| 20 | [mevalonate pathway](http://pathway.yeastgenome.org/YEAST/NEW-IMAGE?type=PATHWAY&object=IPPSYN-PWY) | 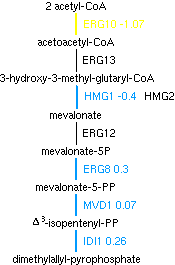 | | [acetoacetyl CoA thiolase](http://pathway.yeastgenome.org/YEAST/NEW-IMAGE?type=ENZYME&object=YPL028W-MONOMER) | [ERG10](http://db.yeastgenome.org/cgi-bin/locus.pl?locus=S000005949) | | --- | --- | | [3-hydroxy-3-methylglutaryl coenzyme A synthase](http://pathway.yeastgenome.org/YEAST/NEW-IMAGE?type=ENZYME&object=YML126C-MONOMER) | [ERG13](http://db.yeastgenome.org/cgi-bin/locus.pl?locus=S000004595) | | [3-hydroxy-3-methylglutaryl-coenzyme A (HMG-CoA)](http://pathway.yeastgenome.org/YEAST/NEW-IMAGE?type=ENZYME&object=YLR450W-MONOMER) | [HMG2](http://db.yeastgenome.org/cgi-bin/locus.pl?locus=S000004442) | | [3-hydroxy-3-methylglutaryl-coenzyme A (HMG-CoA)](http://pathway.yeastgenome.org/YEAST/NEW-IMAGE?type=ENZYME&object=YML075C-MONOMER) | [HMG1](http://db.yeastgenome.org/cgi-bin/locus.pl?locus=S000004540) | | [mevalonate kinase](http://pathway.yeastgenome.org/YEAST/NEW-IMAGE?type=ENZYME&object=YMR208W-MONOMER) | [ERG12](http://db.yeastgenome.org/cgi-bin/locus.pl?locus=S000004821) | | [phosphomevalonate kinase](http://pathway.yeastgenome.org/YEAST/NEW-IMAGE?type=ENZYME&object=YMR220W-MONOMER) | [ERG8](http://db.yeastgenome.org/cgi-bin/locus.pl?locus=S000004833) | | [mevalonate pyrophosphate decarboxylase](http://pathway.yeastgenome.org/YEAST/NEW-IMAGE?type=ENZYME&object=YNR043W-MONOMER) | [MVD1](http://db.yeastgenome.org/cgi-bin/locus.pl?locus=S000005326) | | [isopentenyl diphosphate:dimethylallyl diphosphate isomerase](http://pathway.yeastgenome.org/YEAST/NEW-IMAGE?type=ENZYME&object=YPL117C-MONOMER) | [IDI1](http://db.yeastgenome.org/cgi-bin/locus.pl?locus=S000006038) | |
| 26 | [sphingolipid metabolism](http://pathway.yeastgenome.org/YEAST/NEW-IMAGE?type=PATHWAY&object=SPHINGOLIPID-SYN-PWY) | 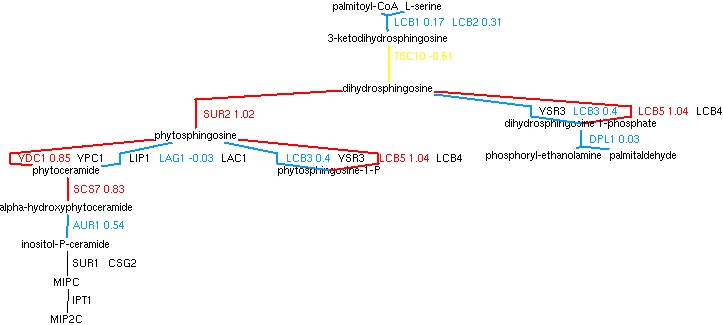 | | [serine palmitoyltransferase](http://pathway.yeastgenome.org/YEAST/NEW-IMAGE?type=ENZYME&object=YDR062W-MONOMER) | [LCB2](http://db.yeastgenome.org/cgi-bin/locus.pl?locus=S000002469) | | --- | --- | | [serine palmitoyltransferase](http://pathway.yeastgenome.org/YEAST/NEW-IMAGE?type=ENZYME&object=YMR296C-MONOMER) | [LCB1](http://db.yeastgenome.org/cgi-bin/locus.pl?locus=S000004911) | | [3-ketosphinganine reductase](http://pathway.yeastgenome.org/YEAST/NEW-IMAGE?type=ENZYME&object=YBR265W-MONOMER) | [TSC10](http://db.yeastgenome.org/cgi-bin/locus.pl?locus=S000000469) | | [dihydrosphingosine C-4 hydroxylase](http://pathway.yeastgenome.org/YEAST/NEW-IMAGE?type=ENZYME&object=YDR297W-MONOMER) | [SUR2](http://db.yeastgenome.org/cgi-bin/locus.pl?locus=S000002705) | | [PHS kinase / DHS kinase](http://pathway.yeastgenome.org/YEAST/NEW-IMAGE?type=ENZYME&object=YOR171C-MONOMER) | [LCB4](http://db.yeastgenome.org/cgi-bin/locus.pl?locus=S000005697) | | [PHS kinase / DHS kinase](http://pathway.yeastgenome.org/YEAST/NEW-IMAGE?type=ENZYME&object=YLR260W-MONOMER) | [LCB5](http://db.yeastgenome.org/cgi-bin/locus.pl?locus=S000004250) | | [PHS-1-P phosphatase / DHS-1-P phosphatase](http://pathway.yeastgenome.org/YEAST/NEW-IMAGE?type=ENZYME&object=YKR053C-MONOMER) | [YSR3](http://db.yeastgenome.org/cgi-bin/locus.pl?locus=S000001761) | | [PHS-1-P phsophatase / DHS-1-P phosphatase](http://pathway.yeastgenome.org/YEAST/NEW-IMAGE?type=ENZYME&object=MONOMER3O-419) | [LCB3](http://db.yeastgenome.org/cgi-bin/locus.pl?locus=S000003670) | | [ceramide synthase](http://pathway.yeastgenome.org/YEAST/NEW-IMAGE?type=ENZYME&object=CPLX3O-78) | [LIP1](http://db.yeastgenome.org/cgi-bin/locus.pl?locus=S000004913) [LAG1](http://db.yeastgenome.org/cgi-bin/locus.pl?locus=S000000995) [LAC1](http://db.yeastgenome.org/cgi-bin/locus.pl?locus=S000001491) | | [ceramidase](http://pathway.yeastgenome.org/YEAST/NEW-IMAGE?type=ENZYME&object=YBR183W-MONOMER) | [YPC1](http://db.yeastgenome.org/cgi-bin/locus.pl?locus=S000000387) | | [ceramidase](http://pathway.yeastgenome.org/YEAST/NEW-IMAGE?type=ENZYME&object=YPL087W-MONOMER) | [YDC1](http://db.yeastgenome.org/cgi-bin/locus.pl?locus=S000006008) | | [desaturase](http://pathway.yeastgenome.org/YEAST/NEW-IMAGE?type=ENZYME&object=YMR272C-MONOMER) | [SCS7](http://db.yeastgenome.org/cgi-bin/locus.pl?locus=S000004885) | | [IPC synthase](http://pathway.yeastgenome.org/YEAST/NEW-IMAGE?type=ENZYME&object=MONOMER3O-630) | [AUR1](http://db.yeastgenome.org/cgi-bin/locus.pl?locus=S000001487) | | [inositol phosphorylceramide mannosyltransferase](http://pathway.yeastgenome.org/YEAST/NEW-IMAGE?type=ENZYME&object=CPLX3O-383) | [SUR1](http://db.yeastgenome.org/cgi-bin/locus.pl?locus=S000005978) [CSG2](http://db.yeastgenome.org/cgi-bin/locus.pl?locus=S000000240) | | [inositolphosphotransferase](http://pathway.yeastgenome.org/YEAST/NEW-IMAGE?type=ENZYME&object=YDR072C-MONOMER) | [IPT1](http://db.yeastgenome.org/cgi-bin/locus.pl?locus=S000002479) | | [dihydrosphingosine phosphate lyase](http://pathway.yeastgenome.org/YEAST/NEW-IMAGE?type=ENZYME&object=YDR294C-MONOMER) | [DPL1](http://db.yeastgenome.org/cgi-bin/locus.pl?locus=S000002702) | |
| 30 | [phospholipid biosynthesis](http://pathway.yeastgenome.org/YEAST/NEW-IMAGE?type=PATHWAY&object=PHOSLIPSYN2-PWY) | 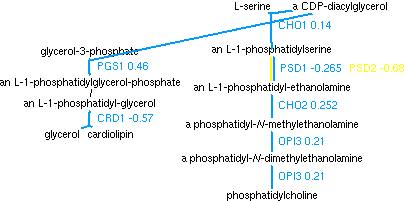 | | [phosphatidylserine synthase](http://pathway.yeastgenome.org/YEAST/NEW-IMAGE?type=ENZYME&object=YER026C-MONOMER) | [CHO1](http://db.yeastgenome.org/cgi-bin/locus.pl?locus=S000000828) | | --- | --- | | [phosphatidylserine decarboxylase, golgi/vacuole](http://pathway.yeastgenome.org/YEAST/NEW-IMAGE?type=ENZYME&object=YGR170W-MONOMER) | [PSD2](http://db.yeastgenome.org/cgi-bin/locus.pl?locus=S000003402) | | [phosphatidylserine decarboxylase, mitochondria](http://pathway.yeastgenome.org/YEAST/NEW-IMAGE?type=ENZYME&object=YNL169C-MONOMER) | [PSD1](http://db.yeastgenome.org/cgi-bin/locus.pl?locus=S000005113) | | [CHO2](http://pathway.yeastgenome.org/YEAST/NEW-IMAGE?type=ENZYME&object=YGR157W-MONOMER) | [CHO2](http://db.yeastgenome.org/cgi-bin/locus.pl?locus=S000003389) | | [OPI3](http://pathway.yeastgenome.org/YEAST/NEW-IMAGE?type=ENZYME&object=YJR073C-MONOMER) | [OPI3](http://db.yeastgenome.org/cgi-bin/locus.pl?locus=S000003834) | | [phosphatidylglycerolphosphate synthase](http://pathway.yeastgenome.org/YEAST/NEW-IMAGE?type=ENZYME&object=YCL004W-MONOMER) | [PGS1](http://db.yeastgenome.org/cgi-bin/locus.pl?locus=S000000510) | | [cardiolipin synthase](http://pathway.yeastgenome.org/YEAST/NEW-IMAGE?type=ENZYME&object=YDL142C-MONOMER) | [CRD1](http://db.yeastgenome.org/cgi-bin/locus.pl?locus=S000002301) | |
| 31 | [phosphatidate biosynthesis I (the dihydroxyacetone pathway)](http://pathway.yeastgenome.org/YEAST/NEW-IMAGE?type=PATHWAY&object=PWY3O-6407) | 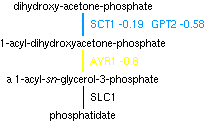 | | [dihydroxyacetone phosphate acyltransferase [multifunctional]](http://pathway.yeastgenome.org/YEAST/NEW-IMAGE?type=ENZYME&object=MONOMER3O-4095) | [GPT2](http://db.yeastgenome.org/cgi-bin/locus.pl?locus=S000001775) | | --- | --- | | [dihydroxyacetone phosphate acyltransferase [multifunctional]](http://pathway.yeastgenome.org/YEAST/NEW-IMAGE?type=ENZYME&object=MONOMER3O-4105) | [SCT1](http://db.yeastgenome.org/cgi-bin/locus.pl?locus=S000000107) | | [1-acyl dihydroxyacetone phosphate reductase](http://pathway.yeastgenome.org/YEAST/NEW-IMAGE?type=ENZYME&object=YIL124W-MONOMER) | [AYR1](http://db.yeastgenome.org/cgi-bin/locus.pl?locus=S000001386) | | [1-acyl-sn-gylcerol-3-phosphate acyl transferase](http://pathway.yeastgenome.org/YEAST/NEW-IMAGE?type=ENZYME&object=YDL052C-MONOMER) | [SLC1](http://db.yeastgenome.org/cgi-bin/locus.pl?locus=S000002210) | |
| 31 | [phosphatidate biosynthesis II (the glycerol-3-phosphate pathway)](http://pathway.yeastgenome.org/YEAST/NEW-IMAGE?type=PATHWAY&object=PWY3O-6499) | 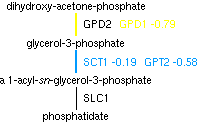 | | [glycerol 3-phosphate dehydrogenase](http://pathway.yeastgenome.org/YEAST/NEW-IMAGE?type=ENZYME&object=YDL022W-MONOMER) | [GPD1](http://db.yeastgenome.org/cgi-bin/locus.pl?locus=S000002180) | | --- | --- | | [glycerol-3-phosphate dehydrogenase (NAD+)](http://pathway.yeastgenome.org/YEAST/NEW-IMAGE?type=ENZYME&object=YOL059W-MONOMER) | [GPD2](http://db.yeastgenome.org/cgi-bin/locus.pl?locus=S000005420) | | [dihydroxyacetone phosphate acyltransferase [multifunctional]](http://pathway.yeastgenome.org/YEAST/NEW-IMAGE?type=ENZYME&object=MONOMER3O-4095) | [GPT2](http://db.yeastgenome.org/cgi-bin/locus.pl?locus=S000001775) | | [dihydroxyacetone phosphate acyltransferase [multifunctional]](http://pathway.yeastgenome.org/YEAST/NEW-IMAGE?type=ENZYME&object=MONOMER3O-4105) | [SCT1](http://db.yeastgenome.org/cgi-bin/locus.pl?locus=S000000107) | | [1-acyl-sn-gylcerol-3-phosphate acyl transferase](http://pathway.yeastgenome.org/YEAST/NEW-IMAGE?type=ENZYME&object=YDL052C-MONOMER) | [SLC1](http://db.yeastgenome.org/cgi-bin/locus.pl?locus=S000002210) | |
| 31 | [triglyceride biosynthesis](http://pathway.yeastgenome.org/YEAST/NEW-IMAGE?type=PATHWAY&object=TRIGLSYN-PWY) | 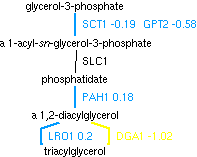 | | [dihydroxyacetone phosphate acyltransferase [multifunctional]](http://pathway.yeastgenome.org/YEAST/NEW-IMAGE?type=ENZYME&object=MONOMER3O-4095) | [GPT2](http://db.yeastgenome.org/cgi-bin/locus.pl?locus=S000001775) | | --- | --- | | [dihydroxyacetone phosphate acyltransferase [multifunctional]](http://pathway.yeastgenome.org/YEAST/NEW-IMAGE?type=ENZYME&object=MONOMER3O-4105) | [SCT1](http://db.yeastgenome.org/cgi-bin/locus.pl?locus=S000000107) | | [1-acyl-sn-gylcerol-3-phosphate acyl transferase](http://pathway.yeastgenome.org/YEAST/NEW-IMAGE?type=ENZYME&object=YDL052C-MONOMER) | [SLC1](http://db.yeastgenome.org/cgi-bin/locus.pl?locus=S000002210) | | [phosphatidate phosphatase](http://pathway.yeastgenome.org/YEAST/NEW-IMAGE?type=ENZYME&object=MONOMER3O-4125) | [PAH1](http://db.yeastgenome.org/cgi-bin/locus.pl?locus=S000004775) | | [Lecithin cholesterol acyl transferase](http://pathway.yeastgenome.org/YEAST/NEW-IMAGE?type=ENZYME&object=YNR008W-MONOMER) | [LRO1](http://db.yeastgenome.org/cgi-bin/locus.pl?locus=S000005291) | | [DiacylGlycerol Acyltransferase](http://pathway.yeastgenome.org/YEAST/NEW-IMAGE?type=ENZYME&object=YOR245C-MONOMER) | [DGA1](http://db.yeastgenome.org/cgi-bin/locus.pl?locus=S000005771) | |
| 33 | [glycerol biosynthesis](http://pathway.yeastgenome.org/YEAST/NEW-IMAGE?type=PATHWAY&object=PWY3O-48) | 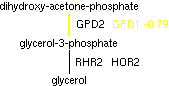 | | [glycrol 3-phosphate dehydrogenase](http://pathway.yeastgenome.org/YEAST/NEW-IMAGE?type=ENZYME&object=YDL022W-MONOMER) | [GPD1](http://db.yeastgenome.org/cgi-bin/locus.pl?locus=S000002180) | | --- | --- | | [glycerol-3-phosphate dehydrogenase (NAD+)](http://pathway.yeastgenome.org/YEAST/NEW-IMAGE?type=ENZYME&object=YOL059W-MONOMER) | [GPD2](http://db.yeastgenome.org/cgi-bin/locus.pl?locus=S000005420) | | [DL-glycerol-3-phosphatase](http://pathway.yeastgenome.org/YEAST/NEW-IMAGE?type=ENZYME&object=YER062C-MONOMER) | [HOR2](http://db.yeastgenome.org/cgi-bin/locus.pl?locus=S000000864) | | [DL-glycerol-3-phosphatase](http://pathway.yeastgenome.org/YEAST/NEW-IMAGE?type=ENZYME&object=YIL053W-MONOMER) | [RHR2](http://db.yeastgenome.org/cgi-bin/locus.pl?locus=S000001315) | |
| 37 | [heme biosynthesis](http://pathway.yeastgenome.org/YEAST/NEW-IMAGE?type=PATHWAY&object=HEME-BIOSYNTHESIS-II) | 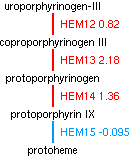 | | [uroporphyrinogen decarboxylase](http://pathway.yeastgenome.org/YEAST/NEW-IMAGE?type=ENZYME&object=YDR047W-MONOMER) | [HEM12](http://db.yeastgenome.org/cgi-bin/locus.pl?locus=S000002454) | | --- | --- | | [aerobic coproporphyrinogen oxidase](http://pathway.yeastgenome.org/YEAST/NEW-IMAGE?type=ENZYME&object=YDR044W-MONOMER) | [HEM13](http://db.yeastgenome.org/cgi-bin/locus.pl?locus=S000002451) | | [protoporphyrinogen oxidase](http://pathway.yeastgenome.org/YEAST/NEW-IMAGE?type=ENZYME&object=YER014W-MONOMER) | [HEM14](http://db.yeastgenome.org/cgi-bin/locus.pl?locus=S000000816) | | [protoheme ferrolyase](http://pathway.yeastgenome.org/YEAST/NEW-IMAGE?type=ENZYME&object=YOR176W-MONOMER) | [HEM15](http://db.yeastgenome.org/cgi-bin/locus.pl?locus=S000005702) | |
| 37 | [siroheme biosynthesis](http://pathway.yeastgenome.org/YEAST/NEW-IMAGE?type=PATHWAY&object=PWY-5194) | 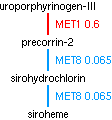 | | [uroporphyrinogen III transmethylase](http://pathway.yeastgenome.org/YEAST/NEW-IMAGE?type=ENZYME&object=MONOMER3O-47) | [MET1](http://db.yeastgenome.org/cgi-bin/locus.pl?locus=S000001777) | | --- | --- | | [ferrochelatase / precorrin-2 dehydrogenase](http://pathway.yeastgenome.org/YEAST/NEW-IMAGE?type=ENZYME&object=MONOMER3O-106) | [MET8](http://db.yeastgenome.org/cgi-bin/locus.pl?locus=S000000417) | |
| 38 | [riboflavin, FMN and FAD biosynthesis](http://pathway.yeastgenome.org/YEAST/NEW-IMAGE?type=PATHWAY&object=YEAST-RIBOSYN-PWY) | 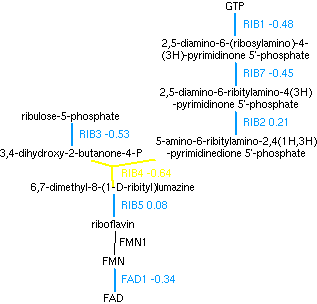 | | [GTP cyclohydrolase II](http://pathway.yeastgenome.org/YEAST/NEW-IMAGE?type=ENZYME&object=YBL033C-MONOMER) | [RIB1](http://db.yeastgenome.org/cgi-bin/locus.pl?locus=S000000129) | | --- | --- | | [diaminohydroxyphoshoribosylaminopyrimidine deaminase](http://pathway.yeastgenome.org/YEAST/NEW-IMAGE?type=ENZYME&object=MONOMER3O-27) | [RIB7](http://db.yeastgenome.org/cgi-bin/locus.pl?locus=S000000357) | | [DRAP deaminase](http://pathway.yeastgenome.org/YEAST/NEW-IMAGE?type=ENZYME&object=YOL066C-MONOMER) | [RIB2](http://db.yeastgenome.org/cgi-bin/locus.pl?locus=S000005427) | | [3,4-dihydroxy-2-butanone-4-phosphate synthase](http://pathway.yeastgenome.org/YEAST/NEW-IMAGE?type=ENZYME&object=MONOMER3O-89) | [RIB3](http://db.yeastgenome.org/cgi-bin/locus.pl?locus=S000002895) | | [lumazine synthase](http://pathway.yeastgenome.org/YEAST/NEW-IMAGE?type=ENZYME&object=YOL143C-MONOMER) | [RIB4](http://db.yeastgenome.org/cgi-bin/locus.pl?locus=S000005503) | | [riboflavine synthetase](http://pathway.yeastgenome.org/YEAST/NEW-IMAGE?type=ENZYME&object=MONOMER3O-75) | [RIB5](http://db.yeastgenome.org/cgi-bin/locus.pl?locus=S000000460) | | [riboflavin kinase](http://pathway.yeastgenome.org/YEAST/NEW-IMAGE?type=ENZYME&object=YDR236C-MONOMER) | [FMN1](http://db.yeastgenome.org/cgi-bin/locus.pl?locus=S000002644) | | [FAD synthetase](http://pathway.yeastgenome.org/YEAST/NEW-IMAGE?type=ENZYME&object=YDL045C-MONOMER) | [FAD1](http://db.yeastgenome.org/cgi-bin/locus.pl?locus=S000002203) | |
| 40 | [tryptophan degradation via kynurenine](http://pathway.yeastgenome.org/YEAST/NEW-IMAGE?type=PATHWAY&object=TRYPTOPHAN-DEGRADATION-1) | 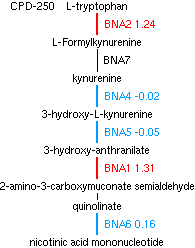 | | [Tryptophan 2,3-dioxygenase](http://pathway.yeastgenome.org/YEAST/NEW-IMAGE?type=ENZYME&object=YJR078W-MONOMER) | [BNA2](http://db.yeastgenome.org/cgi-bin/locus.pl?locus=S000003839) | | --- | --- | | [Arylformamidase](http://pathway.yeastgenome.org/YEAST/NEW-IMAGE?type=ENZYME&object=MONOMER3O-17) | [BNA7](http://db.yeastgenome.org/cgi-bin/locus.pl?locus=S000002836) | | [Kynurenine 3-mono oxygenase](http://pathway.yeastgenome.org/YEAST/NEW-IMAGE?type=ENZYME&object=YBL098W-MONOMER) | [BNA4](http://db.yeastgenome.org/cgi-bin/locus.pl?locus=S000000194) | | [Kynureninase](http://pathway.yeastgenome.org/YEAST/NEW-IMAGE?type=ENZYME&object=YLR231C-MONOMER) | [BNA5](http://db.yeastgenome.org/cgi-bin/locus.pl?locus=S000004221) | | [3-hydroxyanthranilic acid dioxygenase](http://pathway.yeastgenome.org/YEAST/NEW-IMAGE?type=ENZYME&object=YJR025C-MONOMER) | [BNA1](http://db.yeastgenome.org/cgi-bin/locus.pl?locus=S000003786) | | [Quinolinate phosphoribosyl transferase](http://pathway.yeastgenome.org/YEAST/NEW-IMAGE?type=ENZYME&object=YFR047C-MONOMER) | [BNA6](http://db.yeastgenome.org/cgi-bin/locus.pl?locus=S000001943) | |
| 36 - 42 | folate biosynthesis | 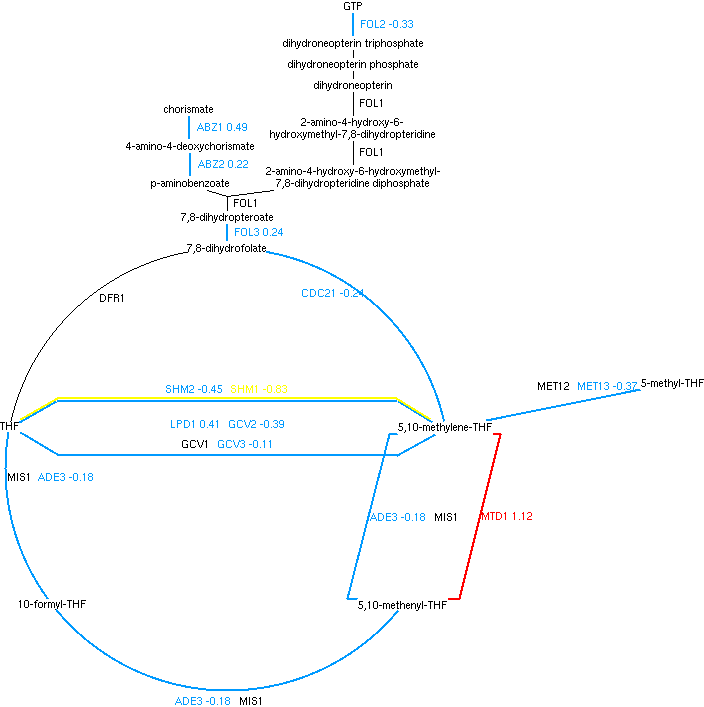 | | [GTP-cyclohydrolase I](http://pathway.yeastgenome.org/YEAST/NEW-IMAGE?type=ENZYME&object=YGR267C-MONOMER) | [FOL2](http://db.yeastgenome.org/cgi-bin/locus.pl?locus=S000003499) | | --- | --- | | [aminodeoxychorismate synthase](http://pathway.yeastgenome.org/YEAST/NEW-IMAGE?type=ENZYME&object=YNR033W-MONOMER) | [ABZ1](http://db.yeastgenome.org/cgi-bin/locus.pl?locus=S000005316) | | [aminodeoxychorismate lyase](http://pathway.yeastgenome.org/YEAST/NEW-IMAGE?type=ENZYME&object=MONOMER3O-131) | [ABZ2](http://db.yeastgenome.org/cgi-bin/locus.pl?locus=S000004902) | | [2-amino-4-hydroxy-6-hydroxymethyldihydropteridine pyrophosphokinase [multifunctional]](http://pathway.yeastgenome.org/YEAST/NEW-IMAGE?type=ENZYME&object=YNL256W-MONOMER) | [FOL1](http://db.yeastgenome.org/cgi-bin/locus.pl?locus=S000005200) | | [dihydrofolate synthase](http://pathway.yeastgenome.org/YEAST/NEW-IMAGE?type=ENZYME&object=YMR113W-MONOMER) | [FOL3](http://db.yeastgenome.org/cgi-bin/locus.pl?locus=S000004719) | | [dihydrofolate reductase](http://pathway.yeastgenome.org/YEAST/NEW-IMAGE?type=ENZYME&object=YOR236W-MONOMER) | [DFR1](http://db.yeastgenome.org/cgi-bin/locus.pl?locus=S000005762) | | [glycine cleavage complex](http://pathway.yeastgenome.org/YEAST/NEW-IMAGE?type=ENZYME&object=CPLX3O-213) | [LPD1](http://db.yeastgenome.org/cgi-bin/locus.pl?locus=S000001876) [GCV2](http://db.yeastgenome.org/cgi-bin/locus.pl?locus=S000004801) [GCV1](http://db.yeastgenome.org/cgi-bin/locus.pl?locus=S000002426) [GCV3](http://db.yeastgenome.org/cgi-bin/locus.pl?locus=S000000042) | | [Serine hydroxymethyltransferase, mitochondrial](http://pathway.yeastgenome.org/YEAST/NEW-IMAGE?type=ENZYME&object=YBR263W-MONOMER) | [SHM1](http://db.yeastgenome.org/cgi-bin/locus.pl?locus=S000000467) | | [serine hydroxymethyltransferase](http://pathway.yeastgenome.org/YEAST/NEW-IMAGE?type=ENZYME&object=YLR058C-MONOMER) | [SHM2](http://db.yeastgenome.org/cgi-bin/locus.pl?locus=S000004048) | | [thymidylate synthase](http://pathway.yeastgenome.org/YEAST/NEW-IMAGE?type=ENZYME&object=CPLX3O-630) | [CDC21](http://db.yeastgenome.org/cgi-bin/locus.pl?locus=S000005600) | | [MTHFR](http://pathway.yeastgenome.org/YEAST/NEW-IMAGE?type=ENZYME&object=YGL125W-MONOMER) | [MET13](http://db.yeastgenome.org/cgi-bin/locus.pl?locus=S000003093) | | [MTHFR](http://pathway.yeastgenome.org/YEAST/NEW-IMAGE?type=ENZYME&object=YPL023C-MONOMER) | [MET12](http://db.yeastgenome.org/cgi-bin/locus.pl?locus=S000005944) | | [NAD-dependent 5,10-methylenetetrahydrafolate dehydrogenase](http://pathway.yeastgenome.org/YEAST/NEW-IMAGE?type=ENZYME&object=CPLX3O-317) | [MTD1](http://db.yeastgenome.org/cgi-bin/locus.pl?locus=S000001788) | | [C1-tetrahydrofolate synthase](http://pathway.yeastgenome.org/YEAST/NEW-IMAGE?type=ENZYME&object=YGR204W-MONOMER) | [ADE3](http://db.yeastgenome.org/cgi-bin/locus.pl?locus=S000003436) | | [mitochondrial C1-tetrahydrofolate synthase](http://pathway.yeastgenome.org/YEAST/NEW-IMAGE?type=ENZYME&object=YBR084W-MONOMER) | [MIS1](http://db.yeastgenome.org/cgi-bin/locus.pl?locus=S000000288) | |
| 41 | [folate polyglutamylation](http://pathway.yeastgenome.org/YEAST/NEW-IMAGE?type=PATHWAY&object=PWY3O-20) | 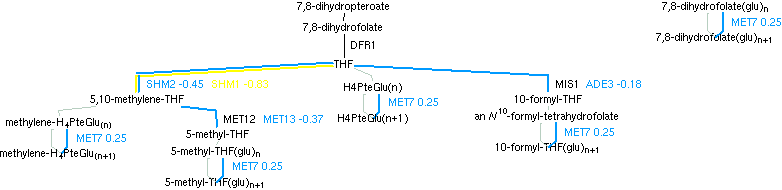 | | [dihydrofolate reductase](http://pathway.yeastgenome.org/YEAST/NEW-IMAGE?type=ENZYME&object=YOR236W-MONOMER) | [DFR1](http://db.yeastgenome.org/cgi-bin/locus.pl?locus=S000005762) | | --- | --- | | [Serine hydroxymethyltransferase, mitochondrial](http://pathway.yeastgenome.org/YEAST/NEW-IMAGE?type=ENZYME&object=YBR263W-MONOMER) | [SHM1](http://db.yeastgenome.org/cgi-bin/locus.pl?locus=S000000467) | | [serine hydroxymethyltransferase](http://pathway.yeastgenome.org/YEAST/NEW-IMAGE?type=ENZYME&object=YLR058C-MONOMER) | [SHM2](http://db.yeastgenome.org/cgi-bin/locus.pl?locus=S000004048) | | [MTHFR](http://pathway.yeastgenome.org/YEAST/NEW-IMAGE?type=ENZYME&object=YGL125W-MONOMER) | [MET13](http://db.yeastgenome.org/cgi-bin/locus.pl?locus=S000003093) | | [MTHFR](http://pathway.yeastgenome.org/YEAST/NEW-IMAGE?type=ENZYME&object=YPL023C-MONOMER) | [MET12](http://db.yeastgenome.org/cgi-bin/locus.pl?locus=S000005944) | | [C1-tetrahydrofolate synthase](http://pathway.yeastgenome.org/YEAST/NEW-IMAGE?type=ENZYME&object=YGR204W-MONOMER) | [ADE3](http://db.yeastgenome.org/cgi-bin/locus.pl?locus=S000003436) | | [mitochondrial C1-tetrahydrofolate synthase](http://pathway.yeastgenome.org/YEAST/NEW-IMAGE?type=ENZYME&object=YBR084W-MONOMER) | [MIS1](http://db.yeastgenome.org/cgi-bin/locus.pl?locus=S000000288) | | [folylpolyglutamate synthetase](http://pathway.yeastgenome.org/YEAST/NEW-IMAGE?type=ENZYME&object=YOR241W-MONOMER) | [MET7](http://db.yeastgenome.org/cgi-bin/locus.pl?locus=S000005767) | |
| 42 | [folate interconversions](http://pathway.yeastgenome.org/YEAST/NEW-IMAGE?type=PATHWAY&object=PWY3O-697) | 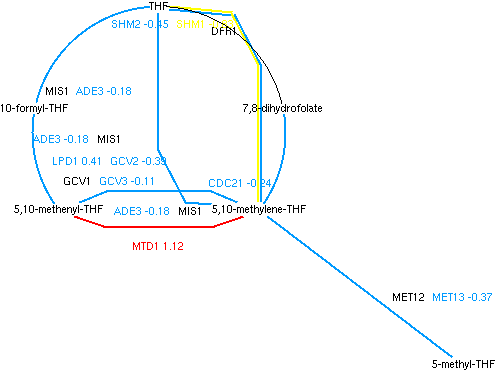 | | [C1-tetrahydrofolate synthase](http://pathway.yeastgenome.org/YEAST/NEW-IMAGE?type=ENZYME&object=YGR204W-MONOMER) | [ADE3](http://db.yeastgenome.org/cgi-bin/locus.pl?locus=S000003436) | | --- | --- | | [mitochondrial C1-tetrahydrofolate synthase](http://pathway.yeastgenome.org/YEAST/NEW-IMAGE?type=ENZYME&object=YBR084W-MONOMER) | [MIS1](http://db.yeastgenome.org/cgi-bin/locus.pl?locus=S000000288) | | [Serine hydroxymethyltransferase, mitochondrial](http://pathway.yeastgenome.org/YEAST/NEW-IMAGE?type=ENZYME&object=YBR263W-MONOMER) | [SHM1](http://db.yeastgenome.org/cgi-bin/locus.pl?locus=S000000467) | | [serine hydroxymethyltransferase](http://pathway.yeastgenome.org/YEAST/NEW-IMAGE?type=ENZYME&object=YLR058C-MONOMER) | [SHM2](http://db.yeastgenome.org/cgi-bin/locus.pl?locus=S000004048) | | [thymidylate synthase](http://pathway.yeastgenome.org/YEAST/NEW-IMAGE?type=ENZYME&object=CPLX3O-630) | [CDC21](http://db.yeastgenome.org/cgi-bin/locus.pl?locus=S000005600) | | [dihydrofolate reductase](http://pathway.yeastgenome.org/YEAST/NEW-IMAGE?type=ENZYME&object=YOR236W-MONOMER) | [DFR1](http://db.yeastgenome.org/cgi-bin/locus.pl?locus=S000005762) | | [glycine cleavage complex](http://pathway.yeastgenome.org/YEAST/NEW-IMAGE?type=ENZYME&object=CPLX3O-213) | [LPD1](http://db.yeastgenome.org/cgi-bin/locus.pl?locus=S000001876) [GCV2](http://db.yeastgenome.org/cgi-bin/locus.pl?locus=S000004801) [GCV1](http://db.yeastgenome.org/cgi-bin/locus.pl?locus=S000002426) [GCV3](http://db.yeastgenome.org/cgi-bin/locus.pl?locus=S000000042) | | [MTHFR](http://pathway.yeastgenome.org/YEAST/NEW-IMAGE?type=ENZYME&object=YGL125W-MONOMER) | [MET13](http://db.yeastgenome.org/cgi-bin/locus.pl?locus=S000003093) | | [MTHFR](http://pathway.yeastgenome.org/YEAST/NEW-IMAGE?type=ENZYME&object=YPL023C-MONOMER) | [MET12](http://db.yeastgenome.org/cgi-bin/locus.pl?locus=S000005944) | | [NAD-dependent 5,10-methylenetetrahydrafolate dehydrogenase](http://pathway.yeastgenome.org/YEAST/NEW-IMAGE?type=ENZYME&object=CPLX3O-317) | [MTD1](http://db.yeastgenome.org/cgi-bin/locus.pl?locus=S000001788) | |
| 43 | [biotin biosynthesis](http://pathway.yeastgenome.org/YEAST/NEW-IMAGE?type=PATHWAY&object=BIOTIN-SYNTHESIS-PWY) | 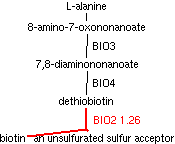 | | [7,8-diamino-pelargonic acid aminotransferase](http://pathway.yeastgenome.org/YEAST/NEW-IMAGE?type=ENZYME&object=YNR058W-MONOMER) | [BIO3](http://db.yeastgenome.org/cgi-bin/locus.pl?locus=S000005341) | | --- | --- | | [dethiobiotin synthetase](http://pathway.yeastgenome.org/YEAST/NEW-IMAGE?type=ENZYME&object=YNR057C-MONOMER) | [BIO4](http://db.yeastgenome.org/cgi-bin/locus.pl?locus=S000005340) | | [biotin synthase](http://pathway.yeastgenome.org/YEAST/NEW-IMAGE?type=ENZYME&object=YGR286C-MONOMER) | [BIO2](http://db.yeastgenome.org/cgi-bin/locus.pl?locus=S000003518) | |
| 44 | [folate transformations](http://pathway.yeastgenome.org/YEAST/NEW-IMAGE?type=PATHWAY&object=PWY-2201) | 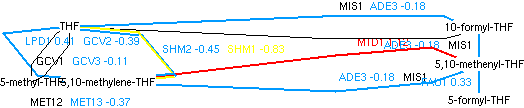 | | [Serine hydroxymethyltransferase, mitochondrial](http://pathway.yeastgenome.org/YEAST/NEW-IMAGE?type=ENZYME&object=YBR263W-MONOMER) | [SHM1](http://db.yeastgenome.org/cgi-bin/locus.pl?locus=S000000467) | | --- | --- | | [serine hydroxymethyltransferase](http://pathway.yeastgenome.org/YEAST/NEW-IMAGE?type=ENZYME&object=YLR058C-MONOMER) | [SHM2](http://db.yeastgenome.org/cgi-bin/locus.pl?locus=S000004048) | | [MTHFR](http://pathway.yeastgenome.org/YEAST/NEW-IMAGE?type=ENZYME&object=YGL125W-MONOMER) | [MET13](http://db.yeastgenome.org/cgi-bin/locus.pl?locus=S000003093) | | [MTHFR](http://pathway.yeastgenome.org/YEAST/NEW-IMAGE?type=ENZYME&object=YPL023C-MONOMER) | [MET12](http://db.yeastgenome.org/cgi-bin/locus.pl?locus=S000005944) | | [glycine cleavage complex](http://pathway.yeastgenome.org/YEAST/NEW-IMAGE?type=ENZYME&object=CPLX3O-213) | [LPD1](http://db.yeastgenome.org/cgi-bin/locus.pl?locus=S000001876) [GCV2](http://db.yeastgenome.org/cgi-bin/locus.pl?locus=S000004801) [GCV1](http://db.yeastgenome.org/cgi-bin/locus.pl?locus=S000002426) [GCV3](http://db.yeastgenome.org/cgi-bin/locus.pl?locus=S000000042) | | [NAD-dependent 5,10-methylenetetrahydrafolate dehydrogenase](http://pathway.yeastgenome.org/YEAST/NEW-IMAGE?type=ENZYME&object=CPLX3O-317) | [MTD1](http://db.yeastgenome.org/cgi-bin/locus.pl?locus=S000001788) | | [mitochondrial C1-tetrahydrofolate synthase](http://pathway.yeastgenome.org/YEAST/NEW-IMAGE?type=ENZYME&object=YBR084W-MONOMER) | [MIS1](http://db.yeastgenome.org/cgi-bin/locus.pl?locus=S000000288) | | [C1-tetrahydrofolate synthase](http://pathway.yeastgenome.org/YEAST/NEW-IMAGE?type=ENZYME&object=YGR204W-MONOMER) | [ADE3](http://db.yeastgenome.org/cgi-bin/locus.pl?locus=S000003436) | | [5,10-methenyltetrahydrofolate synthetase](http://pathway.yeastgenome.org/YEAST/NEW-IMAGE?type=ENZYME&object=YER183C-MONOMER) | [FAU1](http://db.yeastgenome.org/cgi-bin/locus.pl?locus=S000000985) | |
| 45 | [glutathione degradation](http://pathway.yeastgenome.org/YEAST/NEW-IMAGE?type=PATHWAY&object=PWYQT-4432) | 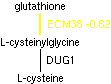 | | [γ-glutamyltransferase](http://pathway.yeastgenome.org/YEAST/NEW-IMAGE?type=ENZYME&object=YLR299W-MONOMER) | [ECM38](http://db.yeastgenome.org/cgi-bin/locus.pl?locus=S000004290) | | --- | --- | | [Cys-Gly metallodipeptidase](http://pathway.yeastgenome.org/YEAST/NEW-IMAGE?type=ENZYME&object=CPLX3O-5) | [DUG1](http://db.yeastgenome.org/cgi-bin/locus.pl?locus=S000001940) | |
| 45 | [superpathway of glutathione metabolism (truncated γ-glutamyl cycle)](http://pathway.yeastgenome.org/YEAST/NEW-IMAGE?type=PATHWAY&object=PWY3O-114) | 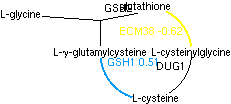 | | [gamma-glutamylcysteine synthetase](http://pathway.yeastgenome.org/YEAST/NEW-IMAGE?type=ENZYME&object=YJL101C-MONOMER) | [GSH1](http://db.yeastgenome.org/cgi-bin/locus.pl?locus=S000003637) | | --- | --- | | [glutathione synthetase](http://pathway.yeastgenome.org/YEAST/NEW-IMAGE?type=ENZYME&object=YOL049W-MONOMER) | [GSH2](http://db.yeastgenome.org/cgi-bin/locus.pl?locus=S000005409) | | [γ-glutamyltransferase](http://pathway.yeastgenome.org/YEAST/NEW-IMAGE?type=ENZYME&object=YLR299W-MONOMER) | [ECM38](http://db.yeastgenome.org/cgi-bin/locus.pl?locus=S000004290) | | [Cys-Gly metallodipeptidase](http://pathway.yeastgenome.org/YEAST/NEW-IMAGE?type=ENZYME&object=CPLX3O-5) | [DUG1](http://db.yeastgenome.org/cgi-bin/locus.pl?locus=S000001940) | |
| 46 | [pyridoxal 5'-phosphate salvage pathway](http://pathway.yeastgenome.org/YEAST/NEW-IMAGE?type=PATHWAY&object=PLPSAL-PWY) | 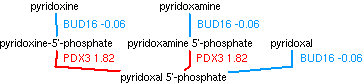 | | [pyridoxal kinase / pyridoxamine kinase / pyridoxine kinase](http://pathway.yeastgenome.org/YEAST/NEW-IMAGE?type=ENZYME&object=CPLX3O-38) | [BUD16](http://db.yeastgenome.org/cgi-bin/locus.pl?locus=S000000755) | | --- | --- | | [pyridoxine phosphate oxidase](http://pathway.yeastgenome.org/YEAST/NEW-IMAGE?type=ENZYME&object=YBR035C-MONOMER) | [PDX3](http://db.yeastgenome.org/cgi-bin/locus.pl?locus=S000000239) | |
| 52 | [salvage pathways of purines and their nucleosides](http://pathway.yeastgenome.org/YEAST/NEW-IMAGE?type=PATHWAY&object=PWY3O-1) | 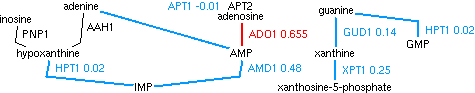 | | [purine nucleoside phosphorylase](http://pathway.yeastgenome.org/YEAST/NEW-IMAGE?type=ENZYME&object=YLR209C-MONOMER) | [PNP1](http://db.yeastgenome.org/cgi-bin/locus.pl?locus=S000004199) | | --- | --- | | [guanine deaminase](http://pathway.yeastgenome.org/YEAST/NEW-IMAGE?type=ENZYME&object=MONOMER3O-12) | [GUD1](http://db.yeastgenome.org/cgi-bin/locus.pl?locus=S000002397) | | [xanthine phosphoribosyl transferase](http://pathway.yeastgenome.org/YEAST/NEW-IMAGE?type=ENZYME&object=YJR133W-MONOMER) | [XPT1](http://db.yeastgenome.org/cgi-bin/locus.pl?locus=S000003894) | | [adenine aminohydrolase](http://pathway.yeastgenome.org/YEAST/NEW-IMAGE?type=ENZYME&object=YNL141W-MONOMER) | [AAH1](http://db.yeastgenome.org/cgi-bin/locus.pl?locus=S000005085) | | [hypoxanthine guanine phosphoribosyltransferase](http://pathway.yeastgenome.org/YEAST/NEW-IMAGE?type=ENZYME&object=YDR399W-MONOMER) | [HPT1](http://db.yeastgenome.org/cgi-bin/locus.pl?locus=S000002807) | | [adenine phosphoribosyltransferase](http://pathway.yeastgenome.org/YEAST/NEW-IMAGE?type=ENZYME&object=YDR441C-MONOMER) | [APT2](http://db.yeastgenome.org/cgi-bin/locus.pl?locus=S000002849) | | [adenine phosphoribosyltransferase](http://pathway.yeastgenome.org/YEAST/NEW-IMAGE?type=ENZYME&object=YML022W-MONOMER) | [APT1](http://db.yeastgenome.org/cgi-bin/locus.pl?locus=S000004484) | | [adenosine kinase](http://pathway.yeastgenome.org/YEAST/NEW-IMAGE?type=ENZYME&object=YJR105W-MONOMER) | [ADO1](http://db.yeastgenome.org/cgi-bin/locus.pl?locus=S000003866) | | [AMD1](http://pathway.yeastgenome.org/YEAST/NEW-IMAGE?type=ENZYME&object=YML035C-MONOMER) | [AMD1](http://db.yeastgenome.org/cgi-bin/locus.pl?locus=S000004498) | |
| 52 | [salvage pathways of adenine, hypoxanthine and their nucleosides](http://pathway.yeastgenome.org/YEAST/NEW-IMAGE?type=PATHWAY&object=PWY3O-2220) | 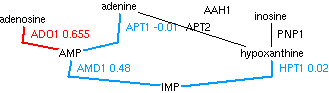 | | [adenosine kinase](http://pathway.yeastgenome.org/YEAST/NEW-IMAGE?type=ENZYME&object=YJR105W-MONOMER) | [ADO1](http://db.yeastgenome.org/cgi-bin/locus.pl?locus=S000003866) | | --- | --- | | [purine nucleoside phosphorylase](http://pathway.yeastgenome.org/YEAST/NEW-IMAGE?type=ENZYME&object=YLR209C-MONOMER) | [PNP1](http://db.yeastgenome.org/cgi-bin/locus.pl?locus=S000004199) | | [adenine phosphoribosyltransferase](http://pathway.yeastgenome.org/YEAST/NEW-IMAGE?type=ENZYME&object=YDR441C-MONOMER) | [APT2](http://db.yeastgenome.org/cgi-bin/locus.pl?locus=S000002849) | | [adenine phosphoribosyltransferase](http://pathway.yeastgenome.org/YEAST/NEW-IMAGE?type=ENZYME&object=YML022W-MONOMER) | [APT1](http://db.yeastgenome.org/cgi-bin/locus.pl?locus=S000004484) | | [AMD1](http://pathway.yeastgenome.org/YEAST/NEW-IMAGE?type=ENZYME&object=YML035C-MONOMER) | [AMD1](http://db.yeastgenome.org/cgi-bin/locus.pl?locus=S000004498) | | [adenine aminohydrolase](http://pathway.yeastgenome.org/YEAST/NEW-IMAGE?type=ENZYME&object=YNL141W-MONOMER) | [AAH1](http://db.yeastgenome.org/cgi-bin/locus.pl?locus=S000005085) | | [hypoxanthine guanine phosphoribosyltransferase](http://pathway.yeastgenome.org/YEAST/NEW-IMAGE?type=ENZYME&object=YDR399W-MONOMER) | [HPT1](http://db.yeastgenome.org/cgi-bin/locus.pl?locus=S000002807) | |
| 53 | [salvage pathways of pyrimidine deoxyribonucleotides](http://pathway.yeastgenome.org/YEAST/NEW-IMAGE?type=PATHWAY&object=YEAST-SALV-PYRMID-DNTP) | 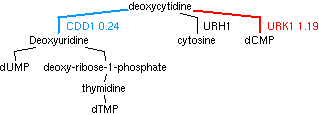 | | [deoxycytidine kinase / cytidine kinase / uridine kinase](http://pathway.yeastgenome.org/YEAST/NEW-IMAGE?type=ENZYME&object=YNR012W-MONOMER) | [URK1](http://db.yeastgenome.org/cgi-bin/locus.pl?locus=S000005295) | | --- | --- | | [nicotinic acid riboside hydrolase [multifunctional]](http://pathway.yeastgenome.org/YEAST/NEW-IMAGE?type=ENZYME&object=YDR400W-MONOMER) | [URH1](http://db.yeastgenome.org/cgi-bin/locus.pl?locus=S000002808) | | [cytidine deaminase](http://pathway.yeastgenome.org/YEAST/NEW-IMAGE?type=ENZYME&object=YLR245C-MONOMER) | [CDD1](http://db.yeastgenome.org/cgi-bin/locus.pl?locus=S000004235) | |
| 54 | [salvage pathways of pyrimidine ribonucleotides](http://pathway.yeastgenome.org/YEAST/NEW-IMAGE?type=PATHWAY&object=YEAST-RNT-SALV) | 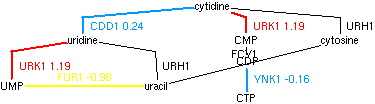 | | [cytosine deaminase](http://pathway.yeastgenome.org/YEAST/NEW-IMAGE?type=ENZYME&object=YPR062W-MONOMER) | [FCY1](http://db.yeastgenome.org/cgi-bin/locus.pl?locus=S000006266) | | --- | --- | | [nucleoside diphosphate kinase](http://pathway.yeastgenome.org/YEAST/NEW-IMAGE?type=ENZYME&object=YKL067W-MONOMER) | [YNK1](http://db.yeastgenome.org/cgi-bin/locus.pl?locus=S000001550) | | [cytidine deaminase](http://pathway.yeastgenome.org/YEAST/NEW-IMAGE?type=ENZYME&object=YLR245C-MONOMER) | [CDD1](http://db.yeastgenome.org/cgi-bin/locus.pl?locus=S000004235) | | [deoxycytidine kinase / cytidine kinase / uridine kinase](http://pathway.yeastgenome.org/YEAST/NEW-IMAGE?type=ENZYME&object=YNR012W-MONOMER) | [URK1](http://db.yeastgenome.org/cgi-bin/locus.pl?locus=S000005295) | | [nicotinic acid riboside hydrolase [multifunctional]](http://pathway.yeastgenome.org/YEAST/NEW-IMAGE?type=ENZYME&object=YDR400W-MONOMER) | [URH1](http://db.yeastgenome.org/cgi-bin/locus.pl?locus=S000002808) | | [UPRTase](http://pathway.yeastgenome.org/YEAST/NEW-IMAGE?type=ENZYME&object=YHR128W-MONOMER) | [FUR1](http://db.yeastgenome.org/cgi-bin/locus.pl?locus=S000001170) | |
| 59 | [lipid-linked oligosaccharide biosynthesis](http://pathway.yeastgenome.org/YEAST/NEW-IMAGE?type=PATHWAY&object=GLUCOSE-MANNOSYL-CHITO-DOLICHOL) | 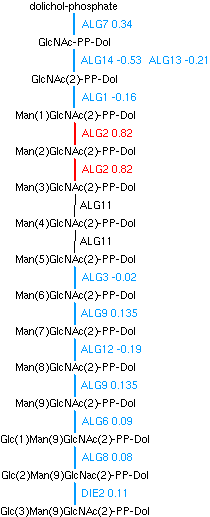 | | [Dol-PP-GlcNAc-1-P-transferase](http://pathway.yeastgenome.org/YEAST/NEW-IMAGE?type=ENZYME&object=YBR243C-MONOMER) | [ALG7](http://db.yeastgenome.org/cgi-bin/locus.pl?locus=S000000447) | | --- | --- | | [UDP-N-acetylglucosamine: N-acetylglucosaminyl-diphosphodolichol N-acetylglucosaminyltransferase](http://pathway.yeastgenome.org/YEAST/NEW-IMAGE?type=ENZYME&object=CPLX3O-24) | [ALG14](http://db.yeastgenome.org/cgi-bin/locus.pl?locus=S000000274) [ALG13](http://db.yeastgenome.org/cgi-bin/locus.pl?locus=S000003015) | | [Dol-PP-GlcNAc2:Man transferase](http://pathway.yeastgenome.org/YEAST/NEW-IMAGE?type=ENZYME&object=YBR110W-MONOMER) | [ALG1](http://db.yeastgenome.org/cgi-bin/locus.pl?locus=S000000314) | | [Dol-PP-GlcNAc2:Man2:Man transferase](http://pathway.yeastgenome.org/YEAST/NEW-IMAGE?type=ENZYME&object=YGL065C-MONOMER) | [ALG2](http://db.yeastgenome.org/cgi-bin/locus.pl?locus=S000003033) | | [alpha-1,2-mannosyltransferase](http://pathway.yeastgenome.org/YEAST/NEW-IMAGE?type=ENZYME&object=MONOMER3O-36) | [ALG11](http://db.yeastgenome.org/cgi-bin/locus.pl?locus=S000004993) | | [Dol-PP-GlcNAc2:Man5:Man transferase](http://pathway.yeastgenome.org/YEAST/NEW-IMAGE?type=ENZYME&object=YBL082C-MONOMER) | [ALG3](http://db.yeastgenome.org/cgi-bin/locus.pl?locus=S000000178) | | [Dol-PP-GlcNAc2:Man7:Man transferase](http://pathway.yeastgenome.org/YEAST/NEW-IMAGE?type=ENZYME&object=MONOMER3O-256) | [ALG12](http://db.yeastgenome.org/cgi-bin/locus.pl?locus=S000005313) | | [Dol-PP-GlcNAc2:Man6:Man transferase](http://pathway.yeastgenome.org/YEAST/NEW-IMAGE?type=ENZYME&object=YNL219C-MONOMER) | [ALG9](http://db.yeastgenome.org/cgi-bin/locus.pl?locus=S000005163) | | [Dol-PP-GlcNAc2:Man9:Glc transferase](http://pathway.yeastgenome.org/YEAST/NEW-IMAGE?type=ENZYME&object=YOR002W-MONOMER) | [ALG6](http://db.yeastgenome.org/cgi-bin/locus.pl?locus=S000005528) | | [Dol-PP-GlcNAc2:Man9:Glc transferase](http://pathway.yeastgenome.org/YEAST/NEW-IMAGE?type=ENZYME&object=YOR067C-MONOMER) | [ALG8](http://db.yeastgenome.org/cgi-bin/locus.pl?locus=S000005593) | | [Dol-PP-GlcNAc2:Man9:Glc transferase](http://pathway.yeastgenome.org/YEAST/NEW-IMAGE?type=ENZYME&object=YGR227W-MONOMER) | [DIE2](http://db.yeastgenome.org/cgi-bin/locus.pl?locus=S000003459) | |
| 61 | [gluconeogenesis](http://pathway.yeastgenome.org/YEAST/NEW-IMAGE?type=PATHWAY&object=GLUCONEO-PWY) | 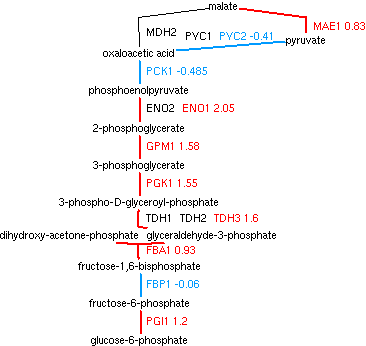 | | [malic enzyme](http://pathway.yeastgenome.org/YEAST/NEW-IMAGE?type=ENZYME&object=YKL029C-MONOMER) | [MAE1](http://db.yeastgenome.org/cgi-bin/locus.pl?locus=S000001512) | | --- | --- | | [pyruvate carboxylase](http://pathway.yeastgenome.org/YEAST/NEW-IMAGE?type=ENZYME&object=YBR218C-MONOMER) | [PYC2](http://db.yeastgenome.org/cgi-bin/locus.pl?locus=S000000422) | | [pyruvate carboxylase](http://pathway.yeastgenome.org/YEAST/NEW-IMAGE?type=ENZYME&object=YGL062W-MONOMER) | [PYC1](http://db.yeastgenome.org/cgi-bin/locus.pl?locus=S000003030) | | [cytosolic malate dehydrogenase](http://pathway.yeastgenome.org/YEAST/NEW-IMAGE?type=ENZYME&object=CPLX3O-88) | [MDH2](http://db.yeastgenome.org/cgi-bin/locus.pl?locus=S000005486) | | [phosphoenolpyruvate carboxylkinase](http://pathway.yeastgenome.org/YEAST/NEW-IMAGE?type=ENZYME&object=YKR097W-MONOMER) | [PCK1](http://db.yeastgenome.org/cgi-bin/locus.pl?locus=S000001805) | | [enolase I](http://pathway.yeastgenome.org/YEAST/NEW-IMAGE?type=ENZYME&object=YGR254W-MONOMER) | [ENO1](http://db.yeastgenome.org/cgi-bin/locus.pl?locus=S000003486) | | [enolase](http://pathway.yeastgenome.org/YEAST/NEW-IMAGE?type=ENZYME&object=YHR174W-MONOMER) | [ENO2](http://db.yeastgenome.org/cgi-bin/locus.pl?locus=S000001217) | | [phosphoglycerate mutase](http://pathway.yeastgenome.org/YEAST/NEW-IMAGE?type=ENZYME&object=YKL152C-MONOMER) | [GPM1](http://db.yeastgenome.org/cgi-bin/locus.pl?locus=S000001635) | | [3-phosphoglycerate kinase](http://pathway.yeastgenome.org/YEAST/NEW-IMAGE?type=ENZYME&object=YCR012W-MONOMER) | [PGK1](http://db.yeastgenome.org/cgi-bin/locus.pl?locus=S000000605) | | [glyceraldehyde-3-phosphate dehydrogenase](http://pathway.yeastgenome.org/YEAST/NEW-IMAGE?type=ENZYME&object=YGR192C-MONOMER) | [TDH3](http://db.yeastgenome.org/cgi-bin/locus.pl?locus=S000003424) | | [glyceraldehyde 3-phosphate dehydrogenase](http://pathway.yeastgenome.org/YEAST/NEW-IMAGE?type=ENZYME&object=YJR009C-MONOMER) | [TDH2](http://db.yeastgenome.org/cgi-bin/locus.pl?locus=S000003769) | | [glyceraldehyde-3-phosphate dehydrogenase](http://pathway.yeastgenome.org/YEAST/NEW-IMAGE?type=ENZYME&object=YJL052W-MONOMER) | [TDH1](http://db.yeastgenome.org/cgi-bin/locus.pl?locus=S000003588) | | [aldolase](http://pathway.yeastgenome.org/YEAST/NEW-IMAGE?type=ENZYME&object=YKL060C-MONOMER) | [FBA1](http://db.yeastgenome.org/cgi-bin/locus.pl?locus=S000001543) | | [fructose-1,6-bisphosphatase](http://pathway.yeastgenome.org/YEAST/NEW-IMAGE?type=ENZYME&object=YLR377C-MONOMER) | [FBP1](http://db.yeastgenome.org/cgi-bin/locus.pl?locus=S000004369) | | [glucose-6-phosphate isomerase](http://pathway.yeastgenome.org/YEAST/NEW-IMAGE?type=ENZYME&object=YBR196C-MONOMER) | [PGI1](http://db.yeastgenome.org/cgi-bin/locus.pl?locus=S000000400) | |
| 64 | [trehalose biosynthesis](http://pathway.yeastgenome.org/YEAST/NEW-IMAGE?type=PATHWAY&object=TRESYN-PWY) | 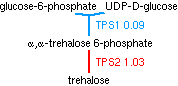 | | [trehalose-6-phosphate synthase](http://pathway.yeastgenome.org/YEAST/NEW-IMAGE?type=ENZYME&object=YBR126C-MONOMER) | [TPS1](http://db.yeastgenome.org/cgi-bin/locus.pl?locus=S000000330) | | --- | --- | | [trehalose-6-phosphate phosphatase](http://pathway.yeastgenome.org/YEAST/NEW-IMAGE?type=ENZYME&object=YDR074W-MONOMER) | [TPS2](http://db.yeastgenome.org/cgi-bin/locus.pl?locus=S000002481) | |
| 66 | [TCA cycle, aerobic respiration](http://pathway.yeastgenome.org/YEAST/NEW-IMAGE?type=PATHWAY&object=TCA-EUK-PWY) | 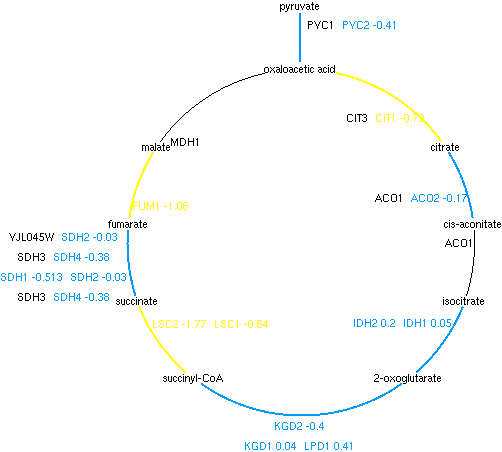 | | [pyruvate carboxylase](http://pathway.yeastgenome.org/YEAST/NEW-IMAGE?type=ENZYME&object=YBR218C-MONOMER) | [PYC2](http://db.yeastgenome.org/cgi-bin/locus.pl?locus=S000000422) | | --- | --- | | [pyruvate carboxylase](http://pathway.yeastgenome.org/YEAST/NEW-IMAGE?type=ENZYME&object=YGL062W-MONOMER) | [PYC1](http://db.yeastgenome.org/cgi-bin/locus.pl?locus=S000003030) | | [citrate synthase](http://pathway.yeastgenome.org/YEAST/NEW-IMAGE?type=ENZYME&object=YNR001C-MONOMER) | [CIT1](http://db.yeastgenome.org/cgi-bin/locus.pl?locus=S000005284) | | [citrate synthase](http://pathway.yeastgenome.org/YEAST/NEW-IMAGE?type=ENZYME&object=YPR001W-MONOMER) | [CIT3](http://db.yeastgenome.org/cgi-bin/locus.pl?locus=S000006205) | | [aconitate hydratase](http://pathway.yeastgenome.org/YEAST/NEW-IMAGE?type=ENZYME&object=YJL200C-MONOMER) | [ACO2](http://db.yeastgenome.org/cgi-bin/locus.pl?locus=S000003736) | | [aconitase](http://pathway.yeastgenome.org/YEAST/NEW-IMAGE?type=ENZYME&object=YLR304C-MONOMER) | [ACO1](http://db.yeastgenome.org/cgi-bin/locus.pl?locus=S000004295) | | [NAD-dependent isocitrate dehydrogenase](http://pathway.yeastgenome.org/YEAST/NEW-IMAGE?type=ENZYME&object=CPLX3O-679) | [IDH2](http://db.yeastgenome.org/cgi-bin/locus.pl?locus=S000005662) [IDH1](http://db.yeastgenome.org/cgi-bin/locus.pl?locus=S000004982) | | [2-ketoglutarate dehydrogenase complex](http://pathway.yeastgenome.org/YEAST/NEW-IMAGE?type=ENZYME&object=CPLX3O-33) | [KGD2](http://db.yeastgenome.org/cgi-bin/locus.pl?locus=S000002555) [KGD1](http://db.yeastgenome.org/cgi-bin/locus.pl?locus=S000001387) [LPD1](http://db.yeastgenome.org/cgi-bin/locus.pl?locus=S000001876) | | [succinyl-CoA ligase](http://pathway.yeastgenome.org/YEAST/NEW-IMAGE?type=ENZYME&object=CPLX3O-690) | [LSC2](http://db.yeastgenome.org/cgi-bin/locus.pl?locus=S000003476) [LSC1](http://db.yeastgenome.org/cgi-bin/locus.pl?locus=S000005668) | | [succinate dehydrogenase (ubiquinone)](http://pathway.yeastgenome.org/YEAST/NEW-IMAGE?type=ENZYME&object=CPLX3O-742) | [SDH1](http://db.yeastgenome.org/cgi-bin/locus.pl?locus=S000001631) [SDH2](http://db.yeastgenome.org/cgi-bin/locus.pl?locus=S000003964) [SDH3](http://db.yeastgenome.org/cgi-bin/locus.pl?locus=S000001624) [SDH4](http://db.yeastgenome.org/cgi-bin/locus.pl?locus=S000002585) | | [minor succinate dehydrogenase (ubiquinone)](http://pathway.yeastgenome.org/YEAST/NEW-IMAGE?type=ENZYME&object=CPLX3O-44) | [YJL045W](http://db.yeastgenome.org/cgi-bin/locus.pl?locus=S000003581) [SDH2](http://db.yeastgenome.org/cgi-bin/locus.pl?locus=S000003964) [SDH3](http://db.yeastgenome.org/cgi-bin/locus.pl?locus=S000001624) [SDH4](http://db.yeastgenome.org/cgi-bin/locus.pl?locus=S000002585) | | [fumarate hydralase](http://pathway.yeastgenome.org/YEAST/NEW-IMAGE?type=ENZYME&object=YPL262W-MONOMER) | [FUM1](http://db.yeastgenome.org/cgi-bin/locus.pl?locus=S000006183) | | [mitochondrial malate dehydrogenase](http://pathway.yeastgenome.org/YEAST/NEW-IMAGE?type=ENZYME&object=CPLX3O-85) | [MDH1](http://db.yeastgenome.org/cgi-bin/locus.pl?locus=S000001568) | |
| 66 | [glyoxylate cycle](http://pathway.yeastgenome.org/YEAST/NEW-IMAGE?type=PATHWAY&object=GLYOXYLATE-BYPASS) | 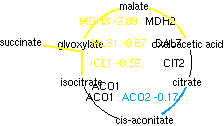 | | [cytosolic malate dehydrogenase](http://pathway.yeastgenome.org/YEAST/NEW-IMAGE?type=ENZYME&object=CPLX3O-88) | [MDH2](http://db.yeastgenome.org/cgi-bin/locus.pl?locus=S000005486) | | --- | --- | | [peroxisome malate dehydrogenase](http://pathway.yeastgenome.org/YEAST/NEW-IMAGE?type=ENZYME&object=CPLX3O-83) | [MDH3](http://db.yeastgenome.org/cgi-bin/locus.pl?locus=S000002236) | | [citrate synthase](http://pathway.yeastgenome.org/YEAST/NEW-IMAGE?type=ENZYME&object=YCR005C-MONOMER) | [CIT2](http://db.yeastgenome.org/cgi-bin/locus.pl?locus=S000000598) | | [aconitate hydratase](http://pathway.yeastgenome.org/YEAST/NEW-IMAGE?type=ENZYME&object=YJL200C-MONOMER) | [ACO2](http://db.yeastgenome.org/cgi-bin/locus.pl?locus=S000003736) | | [aconitase](http://pathway.yeastgenome.org/YEAST/NEW-IMAGE?type=ENZYME&object=YLR304C-MONOMER) | [ACO1](http://db.yeastgenome.org/cgi-bin/locus.pl?locus=S000004295) | | [isocitrate lyase](http://pathway.yeastgenome.org/YEAST/NEW-IMAGE?type=ENZYME&object=YER065C-MONOMER) | [ICL1](http://db.yeastgenome.org/cgi-bin/locus.pl?locus=S000000867) | | [malate synthase 2](http://pathway.yeastgenome.org/YEAST/NEW-IMAGE?type=ENZYME&object=YIR031C-MONOMER) | [DAL7](http://db.yeastgenome.org/cgi-bin/locus.pl?locus=S000001470) | | [malate synthase](http://pathway.yeastgenome.org/YEAST/NEW-IMAGE?type=ENZYME&object=YNL117W-MONOMER) | [MLS1](http://db.yeastgenome.org/cgi-bin/locus.pl?locus=S000005061) | |
| 67 | [non-oxidative branch of the pentose phosphate pathway](http://pathway.yeastgenome.org/YEAST/NEW-IMAGE?type=PATHWAY&object=NONOXIPENT-PWY) | 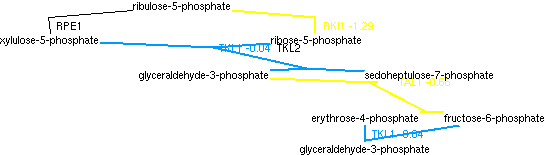 | | [ribose-5-phosphate ketol-isomerase](http://pathway.yeastgenome.org/YEAST/NEW-IMAGE?type=ENZYME&object=YOR095C-MONOMER) | [RKI1](http://db.yeastgenome.org/cgi-bin/locus.pl?locus=S000005621) | | --- | --- | | [D-ribulose-5-Phosphate 3-epimerase](http://pathway.yeastgenome.org/YEAST/NEW-IMAGE?type=ENZYME&object=YJL121C-MONOMER) | [RPE1](http://db.yeastgenome.org/cgi-bin/locus.pl?locus=S000003657) | | [transketolase](http://pathway.yeastgenome.org/YEAST/NEW-IMAGE?type=ENZYME&object=YBR117C-MONOMER) | [TKL2](http://db.yeastgenome.org/cgi-bin/locus.pl?locus=S000000321) | | [transaldolase](http://pathway.yeastgenome.org/YEAST/NEW-IMAGE?type=ENZYME&object=YLR354C-MONOMER) | [TAL1](http://db.yeastgenome.org/cgi-bin/locus.pl?locus=S000004346) | | [transketolase](http://pathway.yeastgenome.org/YEAST/NEW-IMAGE?type=ENZYME&object=YPR074C-MONOMER) | [TKL1](http://db.yeastgenome.org/cgi-bin/locus.pl?locus=S000006278) | |
| 68 | [aerobic respiration, electron transport chain](http://pathway.yeastgenome.org/YEAST/NEW-IMAGE?type=PATHWAY&object=PWY3O-188) | 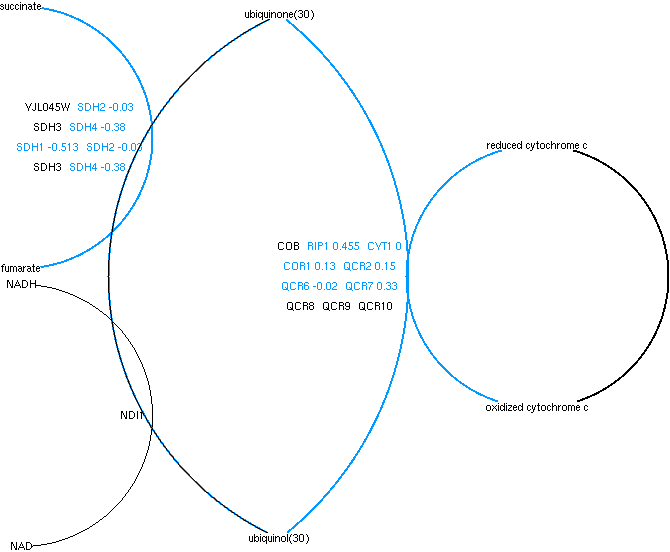 | | [cytochrome c oxidase](http://pathway.yeastgenome.org/YEAST/NEW-IMAGE?type=ENZYME&object=CPLX3O-117) | [COX1](http://db.yeastgenome.org/cgi-bin/locus.pl?locus=S000007260) [COX2](http://db.yeastgenome.org/cgi-bin/locus.pl?locus=S000007281) [COX3](http://db.yeastgenome.org/cgi-bin/locus.pl?locus=S000007283) [COX4](http://db.yeastgenome.org/cgi-bin/locus.pl?locus=S000003155) [COX5A](http://db.yeastgenome.org/cgi-bin/locus.pl?locus=S000004997) [COX6](http://db.yeastgenome.org/cgi-bin/locus.pl?locus=S000001093) [COX7](http://db.yeastgenome.org/cgi-bin/locus.pl?locus=S000004869) [COX8](http://db.yeastgenome.org/cgi-bin/locus.pl?locus=S000004387) [COX9](http://db.yeastgenome.org/cgi-bin/locus.pl?locus=S000002225) [COX12](http://db.yeastgenome.org/cgi-bin/locus.pl?locus=S000004028) [COX13](http://db.yeastgenome.org/cgi-bin/locus.pl?locus=S000003159) | | --- | --- | | [ubiquinol cytochrome c reductase complex](http://pathway.yeastgenome.org/YEAST/NEW-IMAGE?type=ENZYME&object=CPLX3O-109) | [COB](http://db.yeastgenome.org/cgi-bin/locus.pl?locus=S000007270) [RIP1](http://db.yeastgenome.org/cgi-bin/locus.pl?locus=S000000750) [CYT1](http://pathway.yeastgenome.org/YEAST/NEW-IMAGE?type=GENE&object=G3O-102) [COR1](http://db.yeastgenome.org/cgi-bin/locus.pl?locus=S000000141) [QCR2](http://db.yeastgenome.org/cgi-bin/locus.pl?locus=S000006395) [QCR6](http://db.yeastgenome.org/cgi-bin/locus.pl?locus=S000001929) [QCR7](http://db.yeastgenome.org/cgi-bin/locus.pl?locus=S000002937) [QCR8](http://db.yeastgenome.org/cgi-bin/locus.pl?locus=S000003702) [QCR9](http://db.yeastgenome.org/cgi-bin/locus.pl?locus=S000003415) [QCR10](http://db.yeastgenome.org/cgi-bin/locus.pl?locus=S000003529) | | [succinate dehydrogenase (ubiquinone)](http://pathway.yeastgenome.org/YEAST/NEW-IMAGE?type=ENZYME&object=CPLX3O-742) | [SDH1](http://db.yeastgenome.org/cgi-bin/locus.pl?locus=S000001631) [SDH2](http://db.yeastgenome.org/cgi-bin/locus.pl?locus=S000003964) [SDH3](http://db.yeastgenome.org/cgi-bin/locus.pl?locus=S000001624) [SDH4](http://db.yeastgenome.org/cgi-bin/locus.pl?locus=S000002585) | | [minor succinate dehydrogenase (ubiquinone)](http://pathway.yeastgenome.org/YEAST/NEW-IMAGE?type=ENZYME&object=CPLX3O-44) | [YJL045W](http://db.yeastgenome.org/cgi-bin/locus.pl?locus=S000003581) [SDH2](http://db.yeastgenome.org/cgi-bin/locus.pl?locus=S000003964) [SDH3](http://db.yeastgenome.org/cgi-bin/locus.pl?locus=S000001624) [SDH4](http://db.yeastgenome.org/cgi-bin/locus.pl?locus=S000002585) | | [NADH dehydrogenase (ubiquinone)](http://pathway.yeastgenome.org/YEAST/NEW-IMAGE?type=ENZYME&object=YML120C-MONOMER) | [NDI1](http://db.yeastgenome.org/cgi-bin/locus.pl?locus=S000004589) | |
| 69 | [glycolysis](http://pathway.yeastgenome.org/YEAST/NEW-IMAGE?type=PATHWAY&object=GLYCOLYSIS) | 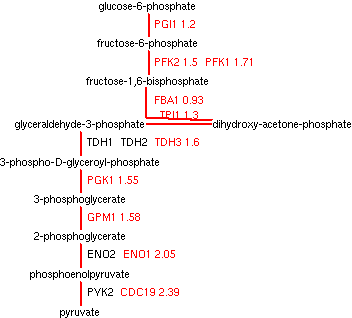 | | [glucose-6-phosphate isomerase](http://pathway.yeastgenome.org/YEAST/NEW-IMAGE?type=ENZYME&object=YBR196C-MONOMER) | [PGI1](http://db.yeastgenome.org/cgi-bin/locus.pl?locus=S000000400) | | --- | --- | | [phosphofructokinase](http://pathway.yeastgenome.org/YEAST/NEW-IMAGE?type=ENZYME&object=CPLX3O-77) | [PFK2](http://db.yeastgenome.org/cgi-bin/locus.pl?locus=S000004818) [PFK1](http://db.yeastgenome.org/cgi-bin/locus.pl?locus=S000003472) | | [aldolase](http://pathway.yeastgenome.org/YEAST/NEW-IMAGE?type=ENZYME&object=YKL060C-MONOMER) | [FBA1](http://db.yeastgenome.org/cgi-bin/locus.pl?locus=S000001543) | | [triosephosphate isomerase](http://pathway.yeastgenome.org/YEAST/NEW-IMAGE?type=ENZYME&object=YDR050C-MONOMER) | [TPI1](http://db.yeastgenome.org/cgi-bin/locus.pl?locus=S000002457) | | [glyceraldehyde-3-phosphate dehydrogenase](http://pathway.yeastgenome.org/YEAST/NEW-IMAGE?type=ENZYME&object=YGR192C-MONOMER) | [TDH3](http://db.yeastgenome.org/cgi-bin/locus.pl?locus=S000003424) | | [glyceraldehyde 3-phosphate dehydrogenase](http://pathway.yeastgenome.org/YEAST/NEW-IMAGE?type=ENZYME&object=YJR009C-MONOMER) | [TDH2](http://db.yeastgenome.org/cgi-bin/locus.pl?locus=S000003769) | | [glyceraldehyde-3-phosphate dehydrogenase](http://pathway.yeastgenome.org/YEAST/NEW-IMAGE?type=ENZYME&object=YJL052W-MONOMER) | [TDH1](http://db.yeastgenome.org/cgi-bin/locus.pl?locus=S000003588) | | [3-phosphoglycerate kinase](http://pathway.yeastgenome.org/YEAST/NEW-IMAGE?type=ENZYME&object=YCR012W-MONOMER) | [PGK1](http://db.yeastgenome.org/cgi-bin/locus.pl?locus=S000000605) | | [phosphoglycerate mutase](http://pathway.yeastgenome.org/YEAST/NEW-IMAGE?type=ENZYME&object=YKL152C-MONOMER) | [GPM1](http://db.yeastgenome.org/cgi-bin/locus.pl?locus=S000001635) | | [enolase I](http://pathway.yeastgenome.org/YEAST/NEW-IMAGE?type=ENZYME&object=YGR254W-MONOMER) | [ENO1](http://db.yeastgenome.org/cgi-bin/locus.pl?locus=S000003486) | | [enolase](http://pathway.yeastgenome.org/YEAST/NEW-IMAGE?type=ENZYME&object=YHR174W-MONOMER) | [ENO2](http://db.yeastgenome.org/cgi-bin/locus.pl?locus=S000001217) | | [pyruvate kinase](http://pathway.yeastgenome.org/YEAST/NEW-IMAGE?type=ENZYME&object=YAL038W-MONOMER) | [CDC19](http://db.yeastgenome.org/cgi-bin/locus.pl?locus=S000000036) | | [pyruvate kinase](http://pathway.yeastgenome.org/YEAST/NEW-IMAGE?type=ENZYME&object=YOR347C-MONOMER) | [PYK2](http://db.yeastgenome.org/cgi-bin/locus.pl?locus=S000005874) | |
| 69 | [superpathway of glucose fermentation](http://pathway.yeastgenome.org/YEAST/NEW-IMAGE?type=PATHWAY&object=GLUCFERMEN-PWY) | 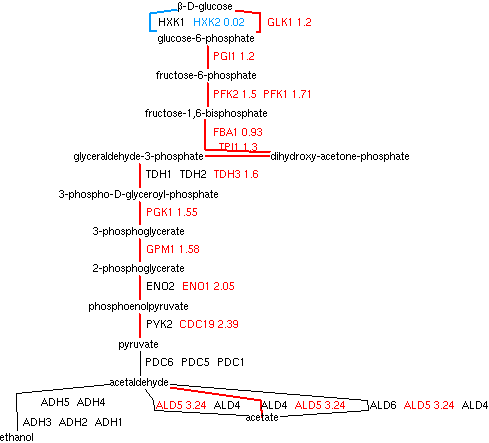 | | [glucokinase](http://pathway.yeastgenome.org/YEAST/NEW-IMAGE?type=ENZYME&object=YCL040W-MONOMER) | [GLK1](http://db.yeastgenome.org/cgi-bin/locus.pl?locus=S000000545) | | --- | --- | | [hexokinase II](http://pathway.yeastgenome.org/YEAST/NEW-IMAGE?type=ENZYME&object=YGL253W-MONOMER) | [HXK2](http://db.yeastgenome.org/cgi-bin/locus.pl?locus=S000003222) | | [hexokinase I](http://pathway.yeastgenome.org/YEAST/NEW-IMAGE?type=ENZYME&object=YFR053C-MONOMER) | [HXK1](http://db.yeastgenome.org/cgi-bin/locus.pl?locus=S000001949) | | [glucose-6-phosphate isomerase](http://pathway.yeastgenome.org/YEAST/NEW-IMAGE?type=ENZYME&object=YBR196C-MONOMER) | [PGI1](http://db.yeastgenome.org/cgi-bin/locus.pl?locus=S000000400) | | [phosphofructokinase](http://pathway.yeastgenome.org/YEAST/NEW-IMAGE?type=ENZYME&object=CPLX3O-77) | [PFK2](http://db.yeastgenome.org/cgi-bin/locus.pl?locus=S000004818) [PFK1](http://db.yeastgenome.org/cgi-bin/locus.pl?locus=S000003472) | | [aldolase](http://pathway.yeastgenome.org/YEAST/NEW-IMAGE?type=ENZYME&object=YKL060C-MONOMER) | [FBA1](http://db.yeastgenome.org/cgi-bin/locus.pl?locus=S000001543) | | [triosephosphate isomerase](http://pathway.yeastgenome.org/YEAST/NEW-IMAGE?type=ENZYME&object=YDR050C-MONOMER) | [TPI1](http://db.yeastgenome.org/cgi-bin/locus.pl?locus=S000002457) | | [glyceraldehyde-3-phosphate dehydrogenase](http://pathway.yeastgenome.org/YEAST/NEW-IMAGE?type=ENZYME&object=YGR192C-MONOMER) | [TDH3](http://db.yeastgenome.org/cgi-bin/locus.pl?locus=S000003424) | | [glyceraldehyde 3-phosphate dehydrogenase](http://pathway.yeastgenome.org/YEAST/NEW-IMAGE?type=ENZYME&object=YJR009C-MONOMER) | [TDH2](http://db.yeastgenome.org/cgi-bin/locus.pl?locus=S000003769) | | [glyceraldehyde-3-phosphate dehydrogenase](http://pathway.yeastgenome.org/YEAST/NEW-IMAGE?type=ENZYME&object=YJL052W-MONOMER) | [TDH1](http://db.yeastgenome.org/cgi-bin/locus.pl?locus=S000003588) | | [3-phosphoglycerate kinase](http://pathway.yeastgenome.org/YEAST/NEW-IMAGE?type=ENZYME&object=YCR012W-MONOMER) | [PGK1](http://db.yeastgenome.org/cgi-bin/locus.pl?locus=S000000605) | | [phosphoglycerate mutase](http://pathway.yeastgenome.org/YEAST/NEW-IMAGE?type=ENZYME&object=YKL152C-MONOMER) | [GPM1](http://db.yeastgenome.org/cgi-bin/locus.pl?locus=S000001635) | | [enolase I](http://pathway.yeastgenome.org/YEAST/NEW-IMAGE?type=ENZYME&object=YGR254W-MONOMER) | [ENO1](http://db.yeastgenome.org/cgi-bin/locus.pl?locus=S000003486) | | [enolase](http://pathway.yeastgenome.org/YEAST/NEW-IMAGE?type=ENZYME&object=YHR174W-MONOMER) | [ENO2](http://db.yeastgenome.org/cgi-bin/locus.pl?locus=S000001217) | | [pyruvate kinase](http://pathway.yeastgenome.org/YEAST/NEW-IMAGE?type=ENZYME&object=YAL038W-MONOMER) | [CDC19](http://db.yeastgenome.org/cgi-bin/locus.pl?locus=S000000036) | | [pyruvate kinase](http://pathway.yeastgenome.org/YEAST/NEW-IMAGE?type=ENZYME&object=YOR347C-MONOMER) | [PYK2](http://db.yeastgenome.org/cgi-bin/locus.pl?locus=S000005874) | | [pyruvate decarboxylase / decarboxylase](http://pathway.yeastgenome.org/YEAST/NEW-IMAGE?type=ENZYME&object=CPLX3O-118) | [PDC1](http://db.yeastgenome.org/cgi-bin/locus.pl?locus=S000004034) | | [pyruvate decarboxylase / decarboxylase](http://pathway.yeastgenome.org/YEAST/NEW-IMAGE?type=ENZYME&object=CPLX3O-67) | [PDC5](http://db.yeastgenome.org/cgi-bin/locus.pl?locus=S000004124) | | [pyruvate decarboxylase / decarboxylase](http://pathway.yeastgenome.org/YEAST/NEW-IMAGE?type=ENZYME&object=CPLX3O-58) | [PDC6](http://db.yeastgenome.org/cgi-bin/locus.pl?locus=S000003319) | | [aldehyde dehydrogenase (major mitochondrial)](http://pathway.yeastgenome.org/YEAST/NEW-IMAGE?type=ENZYME&object=YOR374W-MONOMER) | [ALD4](http://db.yeastgenome.org/cgi-bin/locus.pl?locus=S000005901) | | [aldehyde dehydrogenase (minor mitochondrial)](http://pathway.yeastgenome.org/YEAST/NEW-IMAGE?type=ENZYME&object=YER073W-MONOMER) | [ALD5](http://db.yeastgenome.org/cgi-bin/locus.pl?locus=S000000875) | | [aldehyde dehydrogenase (major cytoplasmic)](http://pathway.yeastgenome.org/YEAST/NEW-IMAGE?type=ENZYME&object=YPL061W-MONOMER) | [ALD6](http://db.yeastgenome.org/cgi-bin/locus.pl?locus=S000005982) | | [alcohol dehydrogenase](http://pathway.yeastgenome.org/YEAST/NEW-IMAGE?type=ENZYME&object=YOL086C-MONOMER) | [ADH1](http://db.yeastgenome.org/cgi-bin/locus.pl?locus=S000005446) | | [alcohol dehydrogenase](http://pathway.yeastgenome.org/YEAST/NEW-IMAGE?type=ENZYME&object=YMR303C-MONOMER) | [ADH2](http://db.yeastgenome.org/cgi-bin/locus.pl?locus=S000004918) | | [alcohol dehydrogenase](http://pathway.yeastgenome.org/YEAST/NEW-IMAGE?type=ENZYME&object=YMR083W-MONOMER) | [ADH3](http://db.yeastgenome.org/cgi-bin/locus.pl?locus=S000004688) | | [alcohol dehydrogenase](http://pathway.yeastgenome.org/YEAST/NEW-IMAGE?type=ENZYME&object=YGL256W-MONOMER) | [ADH4](http://db.yeastgenome.org/cgi-bin/locus.pl?locus=S000003225) | | [alcohol dehydrogenase](http://pathway.yeastgenome.org/YEAST/NEW-IMAGE?type=ENZYME&object=YBR145W-MONOMER) | [ADH5](http://db.yeastgenome.org/cgi-bin/locus.pl?locus=S000000349) | |
| 71 | [fatty acid oxidation pathway](http://pathway.yeastgenome.org/YEAST/NEW-IMAGE?type=PATHWAY&object=YEAST-FAO-PWY) | 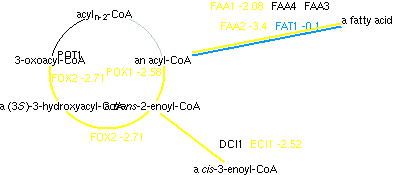 | | [d3,d2-Enoyl-CoA Isomerase](http://pathway.yeastgenome.org/YEAST/NEW-IMAGE?type=ENZYME&object=YLR284C-MONOMER) | [ECI1](http://db.yeastgenome.org/cgi-bin/locus.pl?locus=S000004274) | | --- | --- | | [delta(3,5)-delta(2,4)-dienoyl-CoA isomerase](http://pathway.yeastgenome.org/YEAST/NEW-IMAGE?type=ENZYME&object=YOR180C-MONOMER) | [DCI1](http://db.yeastgenome.org/cgi-bin/locus.pl?locus=S000005706) | | [fatty acid transporter](http://pathway.yeastgenome.org/YEAST/NEW-IMAGE?type=ENZYME&object=YBR041W-MONOMER) | [FAT1](http://db.yeastgenome.org/cgi-bin/locus.pl?locus=S000000245) | | [acyl-CoA synthetase](http://pathway.yeastgenome.org/YEAST/NEW-IMAGE?type=ENZYME&object=YER015W-MONOMER) | [FAA2](http://db.yeastgenome.org/cgi-bin/locus.pl?locus=S000000817) | | [acyl-CoA synthase](http://pathway.yeastgenome.org/YEAST/NEW-IMAGE?type=ENZYME&object=YIL009W-MONOMER) | [FAA3](http://db.yeastgenome.org/cgi-bin/locus.pl?locus=S000001271) | | [long chain fatty acyl:CoA synthetase](http://pathway.yeastgenome.org/YEAST/NEW-IMAGE?type=ENZYME&object=YMR246W-MONOMER) | [FAA4](http://db.yeastgenome.org/cgi-bin/locus.pl?locus=S000004860) | | [long chain fatty acyl:CoA synthetase](http://pathway.yeastgenome.org/YEAST/NEW-IMAGE?type=ENZYME&object=YOR317W-MONOMER) | [FAA1](http://db.yeastgenome.org/cgi-bin/locus.pl?locus=S000005844) | | [fatty-acyl coenzyme A oxidase](http://pathway.yeastgenome.org/YEAST/NEW-IMAGE?type=ENZYME&object=YGL205W-MONOMER) | [POX1](http://db.yeastgenome.org/cgi-bin/locus.pl?locus=S000003173) | | [3-hydroxyacyl-CoA dehydrogenase](http://pathway.yeastgenome.org/YEAST/NEW-IMAGE?type=ENZYME&object=YKR009C-MONOMER) | [FOX2](http://db.yeastgenome.org/cgi-bin/locus.pl?locus=S000001717) | | [3-oxoacyl CoA thiolase](http://pathway.yeastgenome.org/YEAST/NEW-IMAGE?type=ENZYME&object=YIL160C-MONOMER) | [POT1](http://db.yeastgenome.org/cgi-bin/locus.pl?locus=S000001422) | |
| 72 | [formaldehyde oxidation II (glutathione-dependent)](http://pathway.yeastgenome.org/YEAST/NEW-IMAGE?type=PATHWAY&object=PWY-1801) | 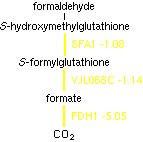 | | [formaldehyde dehydrogenase / alcohol dehydrogenase](http://pathway.yeastgenome.org/YEAST/NEW-IMAGE?type=ENZYME&object=YDL168W-MONOMER) | [SFA1](http://db.yeastgenome.org/cgi-bin/locus.pl?locus=S000002327) | | --- | --- | | [S-formylglutathione hydrolase](http://pathway.yeastgenome.org/YEAST/NEW-IMAGE?type=ENZYME&object=YJL068C-MONOMER) | [YJL068C](http://db.yeastgenome.org/cgi-bin/locus.pl?locus=S000003604) | | [formate dehydrogenases](http://pathway.yeastgenome.org/YEAST/NEW-IMAGE?type=ENZYME&object=YOR388C-MONOMER) | [FDH1](http://db.yeastgenome.org/cgi-bin/locus.pl?locus=S000005915) | |
| 76 | [leucine degradation](http://pathway.yeastgenome.org/YEAST/NEW-IMAGE?type=PATHWAY&object=PWY3O-4112) | 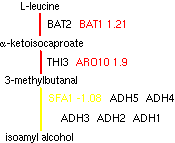 | | [branched-chain amino acid aminotransferase](http://pathway.yeastgenome.org/YEAST/NEW-IMAGE?type=ENZYME&object=YHR208W-MONOMER) | [BAT1](http://db.yeastgenome.org/cgi-bin/locus.pl?locus=S000001251) | | --- | --- | | [branched-chain amino acid transaminase](http://pathway.yeastgenome.org/YEAST/NEW-IMAGE?type=ENZYME&object=YJR148W-MONOMER) | [BAT2](http://db.yeastgenome.org/cgi-bin/locus.pl?locus=S000003909) | | [decarboxylase](http://pathway.yeastgenome.org/YEAST/NEW-IMAGE?type=ENZYME&object=CPLX3O-71) | [ARO10](http://db.yeastgenome.org/cgi-bin/locus.pl?locus=S000002788) | | [ketoisocaproate decarboxylase / decarboxylase](http://pathway.yeastgenome.org/YEAST/NEW-IMAGE?type=ENZYME&object=CPLX3O-110) | [THI3](http://db.yeastgenome.org/cgi-bin/locus.pl?locus=S000002238) | | [alcohol dehydrogenase](http://pathway.yeastgenome.org/YEAST/NEW-IMAGE?type=ENZYME&object=YOL086C-MONOMER) | [ADH1](http://db.yeastgenome.org/cgi-bin/locus.pl?locus=S000005446) | | [alcohol dehydrogenase](http://pathway.yeastgenome.org/YEAST/NEW-IMAGE?type=ENZYME&object=YMR303C-MONOMER) | [ADH2](http://db.yeastgenome.org/cgi-bin/locus.pl?locus=S000004918) | | [alcohol dehydrogenase](http://pathway.yeastgenome.org/YEAST/NEW-IMAGE?type=ENZYME&object=YMR083W-MONOMER) | [ADH3](http://db.yeastgenome.org/cgi-bin/locus.pl?locus=S000004688) | | [alcohol dehydrogenase](http://pathway.yeastgenome.org/YEAST/NEW-IMAGE?type=ENZYME&object=YGL256W-MONOMER) | [ADH4](http://db.yeastgenome.org/cgi-bin/locus.pl?locus=S000003225) | | [alcohol dehydrogenase](http://pathway.yeastgenome.org/YEAST/NEW-IMAGE?type=ENZYME&object=YBR145W-MONOMER) | [ADH5](http://db.yeastgenome.org/cgi-bin/locus.pl?locus=S000000349) | | [formaldehyde dehydrogenase / alcohol dehydrogenase](http://pathway.yeastgenome.org/YEAST/NEW-IMAGE?type=ENZYME&object=YDL168W-MONOMER) | [SFA1](http://db.yeastgenome.org/cgi-bin/locus.pl?locus=S000002327) | |
| 77 | [threonine degradation](http://pathway.yeastgenome.org/YEAST/NEW-IMAGE?type=PATHWAY&object=THREOCAT2-PWY) | 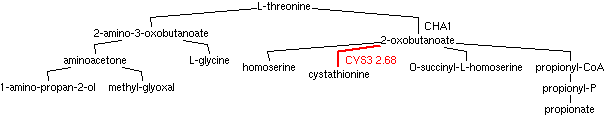 | | [serine/threonine dehydratase](http://pathway.yeastgenome.org/YEAST/NEW-IMAGE?type=ENZYME&object=YCL064C-MONOMER) | [CHA1](http://db.yeastgenome.org/cgi-bin/locus.pl?locus=S000000569) | | --- | --- | | [cystathionine gamma-lyase](http://pathway.yeastgenome.org/YEAST/NEW-IMAGE?type=ENZYME&object=YAL012W-MONOMER) | [CYS3](http://db.yeastgenome.org/cgi-bin/locus.pl?locus=S000000010) | |
| 78 | [valine degradation](http://pathway.yeastgenome.org/YEAST/NEW-IMAGE?type=PATHWAY&object=PWY3O-4105) | 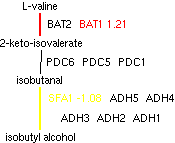 | | [branched-chain amino acid aminotransferase](http://pathway.yeastgenome.org/YEAST/NEW-IMAGE?type=ENZYME&object=YHR208W-MONOMER) | [BAT1](http://db.yeastgenome.org/cgi-bin/locus.pl?locus=S000001251) | | --- | --- | | [branched-chain amino acid transaminase](http://pathway.yeastgenome.org/YEAST/NEW-IMAGE?type=ENZYME&object=YJR148W-MONOMER) | [BAT2](http://db.yeastgenome.org/cgi-bin/locus.pl?locus=S000003909) | | [pyruvate decarboxylase / decarboxylase](http://pathway.yeastgenome.org/YEAST/NEW-IMAGE?type=ENZYME&object=CPLX3O-118) | [PDC1](http://db.yeastgenome.org/cgi-bin/locus.pl?locus=S000004034) | | [pyruvate decarboxylase / decarboxylase](http://pathway.yeastgenome.org/YEAST/NEW-IMAGE?type=ENZYME&object=CPLX3O-67) | [PDC5](http://db.yeastgenome.org/cgi-bin/locus.pl?locus=S000004124) | | [pyruvate decarboxylase / decarboxylase](http://pathway.yeastgenome.org/YEAST/NEW-IMAGE?type=ENZYME&object=CPLX3O-58) | [PDC6](http://db.yeastgenome.org/cgi-bin/locus.pl?locus=S000003319) | | [alcohol dehydrogenase](http://pathway.yeastgenome.org/YEAST/NEW-IMAGE?type=ENZYME&object=YOL086C-MONOMER) | [ADH1](http://db.yeastgenome.org/cgi-bin/locus.pl?locus=S000005446) | | [alcohol dehydrogenase](http://pathway.yeastgenome.org/YEAST/NEW-IMAGE?type=ENZYME&object=YMR303C-MONOMER) | [ADH2](http://db.yeastgenome.org/cgi-bin/locus.pl?locus=S000004918) | | [alcohol dehydrogenase](http://pathway.yeastgenome.org/YEAST/NEW-IMAGE?type=ENZYME&object=YMR083W-MONOMER) | [ADH3](http://db.yeastgenome.org/cgi-bin/locus.pl?locus=S000004688) | | [alcohol dehydrogenase](http://pathway.yeastgenome.org/YEAST/NEW-IMAGE?type=ENZYME&object=YGL256W-MONOMER) | [ADH4](http://db.yeastgenome.org/cgi-bin/locus.pl?locus=S000003225) | | [alcohol dehydrogenase](http://pathway.yeastgenome.org/YEAST/NEW-IMAGE?type=ENZYME&object=YBR145W-MONOMER) | [ADH5](http://db.yeastgenome.org/cgi-bin/locus.pl?locus=S000000349) | | [formaldehyde dehydrogenase / alcohol dehydrogenase](http://pathway.yeastgenome.org/YEAST/NEW-IMAGE?type=ENZYME&object=YDL168W-MONOMER) | [SFA1](http://db.yeastgenome.org/cgi-bin/locus.pl?locus=S000002327) | |
| 79 | [isoleucine degradation](http://pathway.yeastgenome.org/YEAST/NEW-IMAGE?type=PATHWAY&object=PWY3O-4109) | 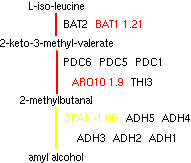 | | [branched-chain amino acid aminotransferase](http://pathway.yeastgenome.org/YEAST/NEW-IMAGE?type=ENZYME&object=YHR208W-MONOMER) | [BAT1](http://db.yeastgenome.org/cgi-bin/locus.pl?locus=S000001251) | | --- | --- | | [branched-chain amino acid transaminase](http://pathway.yeastgenome.org/YEAST/NEW-IMAGE?type=ENZYME&object=YJR148W-MONOMER) | [BAT2](http://db.yeastgenome.org/cgi-bin/locus.pl?locus=S000003909) | | [ketoisocaproate decarboxylase / decarboxylase](http://pathway.yeastgenome.org/YEAST/NEW-IMAGE?type=ENZYME&object=CPLX3O-110) | [THI3](http://db.yeastgenome.org/cgi-bin/locus.pl?locus=S000002238) | | [decarboxylase](http://pathway.yeastgenome.org/YEAST/NEW-IMAGE?type=ENZYME&object=CPLX3O-71) | [ARO10](http://db.yeastgenome.org/cgi-bin/locus.pl?locus=S000002788) | | [pyruvate decarboxylase / decarboxylase](http://pathway.yeastgenome.org/YEAST/NEW-IMAGE?type=ENZYME&object=CPLX3O-118) | [PDC1](http://db.yeastgenome.org/cgi-bin/locus.pl?locus=S000004034) | | [pyruvate decarboxylase / decarboxylase](http://pathway.yeastgenome.org/YEAST/NEW-IMAGE?type=ENZYME&object=CPLX3O-67) | [PDC5](http://db.yeastgenome.org/cgi-bin/locus.pl?locus=S000004124) | | [pyruvate decarboxylase / decarboxylase](http://pathway.yeastgenome.org/YEAST/NEW-IMAGE?type=ENZYME&object=CPLX3O-58) | [PDC6](http://db.yeastgenome.org/cgi-bin/locus.pl?locus=S000003319) | | [alcohol dehydrogenase](http://pathway.yeastgenome.org/YEAST/NEW-IMAGE?type=ENZYME&object=YOL086C-MONOMER) | [ADH1](http://db.yeastgenome.org/cgi-bin/locus.pl?locus=S000005446) | | [alcohol dehydrogenase](http://pathway.yeastgenome.org/YEAST/NEW-IMAGE?type=ENZYME&object=YMR303C-MONOMER) | [ADH2](http://db.yeastgenome.org/cgi-bin/locus.pl?locus=S000004918) | | [alcohol dehydrogenase](http://pathway.yeastgenome.org/YEAST/NEW-IMAGE?type=ENZYME&object=YMR083W-MONOMER) | [ADH3](http://db.yeastgenome.org/cgi-bin/locus.pl?locus=S000004688) | | [alcohol dehydrogenase](http://pathway.yeastgenome.org/YEAST/NEW-IMAGE?type=ENZYME&object=YGL256W-MONOMER) | [ADH4](http://db.yeastgenome.org/cgi-bin/locus.pl?locus=S000003225) | | [alcohol dehydrogenase](http://pathway.yeastgenome.org/YEAST/NEW-IMAGE?type=ENZYME&object=YBR145W-MONOMER) | [ADH5](http://db.yeastgenome.org/cgi-bin/locus.pl?locus=S000000349) | | [formaldehyde dehydrogenase / alcohol dehydrogenase](http://pathway.yeastgenome.org/YEAST/NEW-IMAGE?type=ENZYME&object=YDL168W-MONOMER) | [SFA1](http://db.yeastgenome.org/cgi-bin/locus.pl?locus=S000002327) | |
| 80 | [glutamate degradation I](http://pathway.yeastgenome.org/YEAST/NEW-IMAGE?type=PATHWAY&object=GLUDEG-I-PWY) | 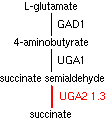 | | [glutamate decarboxylase](http://pathway.yeastgenome.org/YEAST/NEW-IMAGE?type=ENZYME&object=YMR250W-MONOMER) | [GAD1](http://db.yeastgenome.org/cgi-bin/locus.pl?locus=S000004862) | | --- | --- | | [gamma-aminobutyrate (GABA) transaminase](http://pathway.yeastgenome.org/YEAST/NEW-IMAGE?type=ENZYME&object=YGR019W-MONOMER) | [UGA1](http://db.yeastgenome.org/cgi-bin/locus.pl?locus=S000003251) | | [succinate semialdehyde dehydrogenase](http://pathway.yeastgenome.org/YEAST/NEW-IMAGE?type=ENZYME&object=YBR006W-MONOMER) | [UGA2](http://db.yeastgenome.org/cgi-bin/locus.pl?locus=S000000210) | |
| 81 | [tryptophan degradation](http://pathway.yeastgenome.org/YEAST/NEW-IMAGE?type=PATHWAY&object=PWY3O-214) | 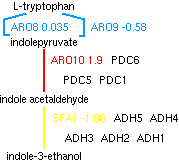 | | [aromatic amino acid aminotransferase II](http://pathway.yeastgenome.org/YEAST/NEW-IMAGE?type=ENZYME&object=YHR137W-MONOMER) | [ARO9](http://db.yeastgenome.org/cgi-bin/locus.pl?locus=S000001179) | | --- | --- | | [aromatic amino acid aminotransferase I](http://pathway.yeastgenome.org/YEAST/NEW-IMAGE?type=ENZYME&object=YGL202W-MONOMER) | [ARO8](http://db.yeastgenome.org/cgi-bin/locus.pl?locus=S000003170) | | [pyruvate decarboxylase / decarboxylase](http://pathway.yeastgenome.org/YEAST/NEW-IMAGE?type=ENZYME&object=CPLX3O-118) | [PDC1](http://db.yeastgenome.org/cgi-bin/locus.pl?locus=S000004034) | | [pyruvate decarboxylase / decarboxylase](http://pathway.yeastgenome.org/YEAST/NEW-IMAGE?type=ENZYME&object=CPLX3O-67) | [PDC5](http://db.yeastgenome.org/cgi-bin/locus.pl?locus=S000004124) | | [pyruvate decarboxylase / decarboxylase](http://pathway.yeastgenome.org/YEAST/NEW-IMAGE?type=ENZYME&object=CPLX3O-58) | [PDC6](http://db.yeastgenome.org/cgi-bin/locus.pl?locus=S000003319) | | [decarboxylase](http://pathway.yeastgenome.org/YEAST/NEW-IMAGE?type=ENZYME&object=CPLX3O-71) | [ARO10](http://db.yeastgenome.org/cgi-bin/locus.pl?locus=S000002788) | | [alcohol dehydrogenase](http://pathway.yeastgenome.org/YEAST/NEW-IMAGE?type=ENZYME&object=YOL086C-MONOMER) | [ADH1](http://db.yeastgenome.org/cgi-bin/locus.pl?locus=S000005446) | | [alcohol dehydrogenase](http://pathway.yeastgenome.org/YEAST/NEW-IMAGE?type=ENZYME&object=YMR303C-MONOMER) | [ADH2](http://db.yeastgenome.org/cgi-bin/locus.pl?locus=S000004918) | | [alcohol dehydrogenase](http://pathway.yeastgenome.org/YEAST/NEW-IMAGE?type=ENZYME&object=YMR083W-MONOMER) | [ADH3](http://db.yeastgenome.org/cgi-bin/locus.pl?locus=S000004688) | | [alcohol dehydrogenase](http://pathway.yeastgenome.org/YEAST/NEW-IMAGE?type=ENZYME&object=YGL256W-MONOMER) | [ADH4](http://db.yeastgenome.org/cgi-bin/locus.pl?locus=S000003225) | | [alcohol dehydrogenase](http://pathway.yeastgenome.org/YEAST/NEW-IMAGE?type=ENZYME&object=YBR145W-MONOMER) | [ADH5](http://db.yeastgenome.org/cgi-bin/locus.pl?locus=S000000349) | | [formaldehyde dehydrogenase / alcohol dehydrogenase](http://pathway.yeastgenome.org/YEAST/NEW-IMAGE?type=ENZYME&object=YDL168W-MONOMER) | [SFA1](http://db.yeastgenome.org/cgi-bin/locus.pl?locus=S000002327) | |
| 82 | [phenylalanine degradation](http://pathway.yeastgenome.org/YEAST/NEW-IMAGE?type=PATHWAY&object=PWY3O-4115) | 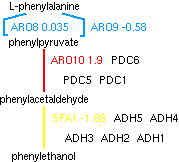 | | [aromatic amino acid aminotransferase II](http://pathway.yeastgenome.org/YEAST/NEW-IMAGE?type=ENZYME&object=YHR137W-MONOMER) | [ARO9](http://db.yeastgenome.org/cgi-bin/locus.pl?locus=S000001179) | | --- | --- | | [aromatic amino acid aminotransferase I](http://pathway.yeastgenome.org/YEAST/NEW-IMAGE?type=ENZYME&object=YGL202W-MONOMER) | [ARO8](http://db.yeastgenome.org/cgi-bin/locus.pl?locus=S000003170) | | [pyruvate decarboxylase / decarboxylase](http://pathway.yeastgenome.org/YEAST/NEW-IMAGE?type=ENZYME&object=CPLX3O-118) | [PDC1](http://db.yeastgenome.org/cgi-bin/locus.pl?locus=S000004034) | | [pyruvate decarboxylase / decarboxylase](http://pathway.yeastgenome.org/YEAST/NEW-IMAGE?type=ENZYME&object=CPLX3O-67) | [PDC5](http://db.yeastgenome.org/cgi-bin/locus.pl?locus=S000004124) | | [pyruvate decarboxylase / decarboxylase](http://pathway.yeastgenome.org/YEAST/NEW-IMAGE?type=ENZYME&object=CPLX3O-58) | [PDC6](http://db.yeastgenome.org/cgi-bin/locus.pl?locus=S000003319) | | [decarboxylase](http://pathway.yeastgenome.org/YEAST/NEW-IMAGE?type=ENZYME&object=CPLX3O-71) | [ARO10](http://db.yeastgenome.org/cgi-bin/locus.pl?locus=S000002788) | | [alcohol dehydrogenase](http://pathway.yeastgenome.org/YEAST/NEW-IMAGE?type=ENZYME&object=YOL086C-MONOMER) | [ADH1](http://db.yeastgenome.org/cgi-bin/locus.pl?locus=S000005446) | | [alcohol dehydrogenase](http://pathway.yeastgenome.org/YEAST/NEW-IMAGE?type=ENZYME&object=YMR303C-MONOMER) | [ADH2](http://db.yeastgenome.org/cgi-bin/locus.pl?locus=S000004918) | | [alcohol dehydrogenase](http://pathway.yeastgenome.org/YEAST/NEW-IMAGE?type=ENZYME&object=YMR083W-MONOMER) | [ADH3](http://db.yeastgenome.org/cgi-bin/locus.pl?locus=S000004688) | | [alcohol dehydrogenase](http://pathway.yeastgenome.org/YEAST/NEW-IMAGE?type=ENZYME&object=YGL256W-MONOMER) | [ADH4](http://db.yeastgenome.org/cgi-bin/locus.pl?locus=S000003225) | | [alcohol dehydrogenase](http://pathway.yeastgenome.org/YEAST/NEW-IMAGE?type=ENZYME&object=YBR145W-MONOMER) | [ADH5](http://db.yeastgenome.org/cgi-bin/locus.pl?locus=S000000349) | | [formaldehyde dehydrogenase / alcohol dehydrogenase](http://pathway.yeastgenome.org/YEAST/NEW-IMAGE?type=ENZYME&object=YDL168W-MONOMER) | [SFA1](http://db.yeastgenome.org/cgi-bin/locus.pl?locus=S000002327) | |
| 84 | [asparagine degradation](http://pathway.yeastgenome.org/YEAST/NEW-IMAGE?type=PATHWAY&object=ASPARAGINE-DEG2-PWY) | 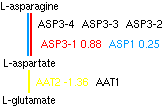 | | [asparaginase I](http://pathway.yeastgenome.org/YEAST/NEW-IMAGE?type=ENZYME&object=YDR321W-MONOMER) | [ASP1](http://db.yeastgenome.org/cgi-bin/locus.pl?locus=S000002729) | | --- | --- | | [asparaginase II](http://pathway.yeastgenome.org/YEAST/NEW-IMAGE?type=ENZYME&object=YLR155C-MONOMER) | [ASP3-1](http://db.yeastgenome.org/cgi-bin/locus.pl?locus=S000004145) | | [asparaginase II](http://pathway.yeastgenome.org/YEAST/NEW-IMAGE?type=ENZYME&object=YLR157C-MONOMER) | [ASP3-2](http://db.yeastgenome.org/cgi-bin/locus.pl?locus=S000004147) | | [asparaginase II](http://pathway.yeastgenome.org/YEAST/NEW-IMAGE?type=ENZYME&object=YLR158C-MONOMER) | [ASP3-3](http://db.yeastgenome.org/cgi-bin/locus.pl?locus=S000004148) | | [asparaginase II](http://pathway.yeastgenome.org/YEAST/NEW-IMAGE?type=ENZYME&object=YLR160C-MONOMER) | [ASP3-4](http://db.yeastgenome.org/cgi-bin/locus.pl?locus=S000004150) | | [aspartate aminotransferase](http://pathway.yeastgenome.org/YEAST/NEW-IMAGE?type=ENZYME&object=YKL106W-MONOMER) | [AAT1](http://db.yeastgenome.org/cgi-bin/locus.pl?locus=S000001589) | | [aspartate aminotransferase](http://pathway.yeastgenome.org/YEAST/NEW-IMAGE?type=ENZYME&object=YLR027C-MONOMER) | [AAT2](http://db.yeastgenome.org/cgi-bin/locus.pl?locus=S000004017) | |
| 89 | [alanine degradation](http://pathway.yeastgenome.org/YEAST/NEW-IMAGE?type=PATHWAY&object=ALANINE-DEG3-PWY) | 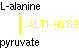 | | [alanine aminotransferase](http://pathway.yeastgenome.org/YEAST/NEW-IMAGE?type=ENZYME&object=YLR089C-MONOMER) | [ALT1](http://db.yeastgenome.org/cgi-bin/locus.pl?locus=S000004079) | | --- | --- | |
| 89 | [alanine biosynthesis](http://pathway.yeastgenome.org/YEAST/NEW-IMAGE?type=PATHWAY&object=ALANINE-SYN2-PWY) | 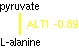 | | [alanine aminotransferase](http://pathway.yeastgenome.org/YEAST/NEW-IMAGE?type=ENZYME&object=YLR089C-MONOMER) | [ALT1](http://db.yeastgenome.org/cgi-bin/locus.pl?locus=S000004079) | | --- | --- | |
| 91 | [mannose degradation](http://pathway.yeastgenome.org/YEAST/NEW-IMAGE?type=PATHWAY&object=PWY3O-1743) | 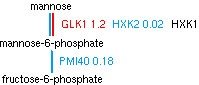 | | [hexokinase I](http://pathway.yeastgenome.org/YEAST/NEW-IMAGE?type=ENZYME&object=YFR053C-MONOMER) | [HXK1](http://db.yeastgenome.org/cgi-bin/locus.pl?locus=S000001949) | | --- | --- | | [hexokinase II](http://pathway.yeastgenome.org/YEAST/NEW-IMAGE?type=ENZYME&object=YGL253W-MONOMER) | [HXK2](http://db.yeastgenome.org/cgi-bin/locus.pl?locus=S000003222) | | [glucokinase](http://pathway.yeastgenome.org/YEAST/NEW-IMAGE?type=ENZYME&object=YCL040W-MONOMER) | [GLK1](http://db.yeastgenome.org/cgi-bin/locus.pl?locus=S000000545) | | [mannose-6-phosphate isomerase](http://pathway.yeastgenome.org/YEAST/NEW-IMAGE?type=ENZYME&object=YER003C-MONOMER) | [PMI40](http://db.yeastgenome.org/cgi-bin/locus.pl?locus=S000000805) | |
| 93 | [glycogen catabolism](http://pathway.yeastgenome.org/YEAST/NEW-IMAGE?type=PATHWAY&object=GLYCOCAT-YEAST-PWY) | 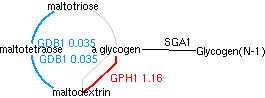 | | [glucoamylase](http://pathway.yeastgenome.org/YEAST/NEW-IMAGE?type=ENZYME&object=YIL099W-MONOMER) | [SGA1](http://db.yeastgenome.org/cgi-bin/locus.pl?locus=S000001361) | | --- | --- | | [glycogen phosphorylase](http://pathway.yeastgenome.org/YEAST/NEW-IMAGE?type=ENZYME&object=YPR160W-MONOMER) | [GPH1](http://db.yeastgenome.org/cgi-bin/locus.pl?locus=S000006364) | | [glucanotranferase](http://pathway.yeastgenome.org/YEAST/NEW-IMAGE?type=ENZYME&object=YPR184W-MONOMER) | [GDB1](http://db.yeastgenome.org/cgi-bin/locus.pl?locus=S000006388) | |
| 99 | [pyruvate dehydrogenase complex](http://pathway.yeastgenome.org/YEAST/NEW-IMAGE?type=PATHWAY&object=PYRUVDEHYD-PWY) | 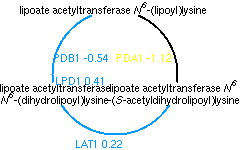 | | [dihydrolipoamide dehydrogenase](http://pathway.yeastgenome.org/YEAST/NEW-IMAGE?type=ENZYME&object=YFL018C-MONOMER) | [LPD1](http://db.yeastgenome.org/cgi-bin/locus.pl?locus=S000001876) | | --- | --- | | [pyruvate dehydrogenase](http://pathway.yeastgenome.org/YEAST/NEW-IMAGE?type=ENZYME&object=YER178W-MONOMER) | [PDA1](http://db.yeastgenome.org/cgi-bin/locus.pl?locus=S000000980) | | [pyruvate dehydrogenase](http://pathway.yeastgenome.org/YEAST/NEW-IMAGE?type=ENZYME&object=YBR221C-MONOMER) | [PDB1](http://db.yeastgenome.org/cgi-bin/locus.pl?locus=S000000425) | | [dihydrolipoamide acetyltransferase](http://pathway.yeastgenome.org/YEAST/NEW-IMAGE?type=ENZYME&object=YNL071W-MONOMER) | [LAT1](http://db.yeastgenome.org/cgi-bin/locus.pl?locus=S000005015) | |
| 100 | [allantoin degradation](http://pathway.yeastgenome.org/YEAST/NEW-IMAGE?type=PATHWAY&object=ALLANTOINDEG-PWY) | 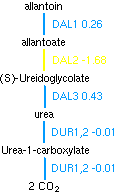 | | [allantoinase](http://pathway.yeastgenome.org/YEAST/NEW-IMAGE?type=ENZYME&object=YIR027C-MONOMER) | [DAL1](http://db.yeastgenome.org/cgi-bin/locus.pl?locus=S000001466) | | --- | --- | | [allantoicase](http://pathway.yeastgenome.org/YEAST/NEW-IMAGE?type=ENZYME&object=YIR029W-MONOMER) | [DAL2](http://db.yeastgenome.org/cgi-bin/locus.pl?locus=S000001468) | | [ureidoglycolate hydrolase](http://pathway.yeastgenome.org/YEAST/NEW-IMAGE?type=ENZYME&object=YIR032C-MONOMER) | [DAL3](http://db.yeastgenome.org/cgi-bin/locus.pl?locus=S000001471) | | [urea carboxylase / allophanate hydrolase](http://pathway.yeastgenome.org/YEAST/NEW-IMAGE?type=ENZYME&object=YBR208C-MONOMER) | [DUR1,2](http://db.yeastgenome.org/cgi-bin/locus.pl?locus=S000000412) | |
| 101 | [4-aminobutyrate degradation](http://pathway.yeastgenome.org/YEAST/NEW-IMAGE?type=PATHWAY&object=YEAST-4AMINOBUTMETAB-PWY) | 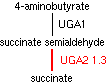 | | [gamma-aminobutyrate (GABA) transaminase](http://pathway.yeastgenome.org/YEAST/NEW-IMAGE?type=ENZYME&object=YGR019W-MONOMER) | [UGA1](http://db.yeastgenome.org/cgi-bin/locus.pl?locus=S000003251) | | --- | --- | | [succinate semialdehyde dehydrogenase](http://pathway.yeastgenome.org/YEAST/NEW-IMAGE?type=ENZYME&object=YBR006W-MONOMER) | [UGA2](http://db.yeastgenome.org/cgi-bin/locus.pl?locus=S000000210) | |
| 102 | [glycerol degradation](http://pathway.yeastgenome.org/YEAST/NEW-IMAGE?type=PATHWAY&object=AERO-GLYCEROL-CAT-PWY) | 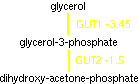 | | [glycerol kinase](http://pathway.yeastgenome.org/YEAST/NEW-IMAGE?type=ENZYME&object=YHL032C-MONOMER) | [GUT1](http://db.yeastgenome.org/cgi-bin/locus.pl?locus=S000001024) | | --- | --- | | [glycerol-3-phosphate dehydrogenase](http://pathway.yeastgenome.org/YEAST/NEW-IMAGE?type=ENZYME&object=YIL155C-MONOMER) | [GUT2](http://db.yeastgenome.org/cgi-bin/locus.pl?locus=S000001417) | |
|  | [glycogen biosynthesis](http://pathway.yeastgenome.org/YEAST/NEW-IMAGE?type=PATHWAY&object=PWY3O-4031) | 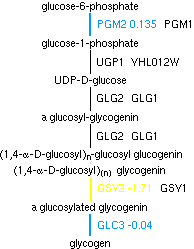 | | [phosphoglucomutase](http://pathway.yeastgenome.org/YEAST/NEW-IMAGE?type=ENZYME&object=YKL127W-MONOMER) | [PGM1](http://db.yeastgenome.org/cgi-bin/locus.pl?locus=S000001610) | | --- | --- | | [phosphoglucomutase](http://pathway.yeastgenome.org/YEAST/NEW-IMAGE?type=ENZYME&object=YMR105C-MONOMER) | [PGM2](http://db.yeastgenome.org/cgi-bin/locus.pl?locus=S000004711) | | [UTP glucose-1-phosphate uridylyltransferase](http://pathway.yeastgenome.org/YEAST/NEW-IMAGE?type=ENZYME&object=YHL012W-MONOMER) | [YHL012W](http://db.yeastgenome.org/cgi-bin/locus.pl?locus=S000001004) | | [uridinephosphoglucose pyrophosphorylase](http://pathway.yeastgenome.org/YEAST/NEW-IMAGE?type=ENZYME&object=YKL035W-MONOMER) | [UGP1](http://db.yeastgenome.org/cgi-bin/locus.pl?locus=S000001518) | | [glycogenin glucosyltransferase](http://pathway.yeastgenome.org/YEAST/NEW-IMAGE?type=ENZYME&object=MONOMER3O-4054) | [GLG1](http://db.yeastgenome.org/cgi-bin/locus.pl?locus=S000001766) | | [glycogenin glucosyltransferase](http://pathway.yeastgenome.org/YEAST/NEW-IMAGE?type=ENZYME&object=MONOMER3O-4031) | [GLG2](http://db.yeastgenome.org/cgi-bin/locus.pl?locus=S000003673) | | [UDP-glucose-starch glucosyltransferase](http://pathway.yeastgenome.org/YEAST/NEW-IMAGE?type=ENZYME&object=YFR015C-MONOMER) | [GSY1](http://db.yeastgenome.org/cgi-bin/locus.pl?locus=S000001911) | | [UDP-glucose-starch glucosyltransferase](http://pathway.yeastgenome.org/YEAST/NEW-IMAGE?type=ENZYME&object=YLR258W-MONOMER) | [GSY2](http://db.yeastgenome.org/cgi-bin/locus.pl?locus=S000004248) | | [1,4-glucan-6-(1,4-glucano)-transferase](http://pathway.yeastgenome.org/YEAST/NEW-IMAGE?type=ENZYME&object=YEL011W-MONOMER) | [GLC3](http://db.yeastgenome.org/cgi-bin/locus.pl?locus=S000000737) | |
