# Supplementary material for: The impact of oxygen on the transcriptome of recombinant S. cerevisiae and P. pastoris - a comparative analysis
Source: BMC Genomics. 2011 May 9;12:218. doi: 10.1186/1471-2164-12-218 (PMC3116504; doi:10.1186/1471-2164-12-218)
Supplement: Additional file 4 — Metabolic pathways. List of metabolic pathways that correspond to the numbers indicated on the cellular overview chart depicted in Figure 3. [file 1471-2164-12-218-S4.DOC]

| 1 | [histidine biosynthesis](http://pathway.yeastgenome.org/YEAST/NEW-IMAGE?object=HISTSYN-PWY) | 53 | [salvage pathways of pyrimidine deoxyribonucleotides](http://pathway.yeastgenome.org/YEAST/NEW-IMAGE?object=YEAST-SALV-PYRMID-DNTP) |
| --- | --- | --- | --- |
| 2 | [aspartate biosynthesis](http://pathway.yeastgenome.org/YEAST/NEW-IMAGE?object=ASPBIO-PWY) | 54 | [salvage pathways of pyrimidine ribonucleotides](http://pathway.yeastgenome.org/YEAST/NEW-IMAGE?object=YEAST-RNT-SALV) |
| 3 | [asparagine biosynthesis](http://pathway.yeastgenome.org/YEAST/NEW-IMAGE?object=ASPARAGINE-BIOSYNTHESIS) | 55 | [UDP-N-acetylglucosamine biosynthesis](http://pathway.yeastgenome.org/YEAST/NEW-IMAGE?object=UDPNAGSYN-YEAST-PWY) |
| 4 | [pantothenate and coenzyme A biosynthesis](http://pathway.yeastgenome.org/YEAST/NEW-IMAGE?object=PANTOSYN2-PWY) | 56 | [inositol phosphate biosynthesis](http://pathway.yeastgenome.org/YEAST/NEW-IMAGE?object=PWY3O-402) |
| 5 | [glutamine biosynthesis](http://pathway.yeastgenome.org/YEAST/NEW-IMAGE?object=GLNSYN-PWY) | 57 | [superpathway of polyamine biosynthesis](http://pathway.yeastgenome.org/YEAST/NEW-IMAGE?object=POLYAMSYN-YEAST-PWY) |
| 6 | [lysine biosynthesis](http://pathway.yeastgenome.org/YEAST/NEW-IMAGE?object=LYSINE-AMINOAD-PWY) | 58 | [*myo*-inositol biosynthesis](http://pathway.yeastgenome.org/YEAST/NEW-IMAGE?object=PWY-2301) |
| 7 | [alanine biosynthesis](http://pathway.yeastgenome.org/YEAST/NEW-IMAGE?object=ALANINE-SYN2-PWY) | 59 | [lipid-linked oligosaccharide biosynthesis](http://pathway.yeastgenome.org/YEAST/NEW-IMAGE?object=GLUCOSE-MANNOSYL-CHITO-DOLICHOL) |
| 8 | [arginine biosynthesis](http://pathway.yeastgenome.org/YEAST/NEW-IMAGE?object=YEAST-ARG-SYN-PWY) | 60 | [chitosan biosynthesis](http://pathway.yeastgenome.org/YEAST/NEW-IMAGE?object=PWY3O-70) |
| 9 | [arginine degradation (anaerobic)](http://pathway.yeastgenome.org/YEAST/NEW-IMAGE?object=ARG-PRO-PWY) | 61 | [gluconeogenesis](http://pathway.yeastgenome.org/YEAST/NEW-IMAGE?object=GLUCONEO-PWY) |
| 10 | [arginine biosynthesis](http://pathway.yeastgenome.org/YEAST/NEW-IMAGE?object=ARGSYNBSUB-PWY) | 62 | [dolichyl glucosyl phosphate biosynthesis](http://pathway.yeastgenome.org/YEAST/NEW-IMAGE?object=PWY3O-1565) |
| 11 | [superpathway of leucine, isoleucine, and valine biosynthesis](http://pathway.yeastgenome.org/YEAST/NEW-IMAGE?object=BRANCHED-CHAIN-AA-SYN-PWY) | 63 | [dolichyl phosphate D-mannose biosynthesis](http://pathway.yeastgenome.org/YEAST/NEW-IMAGE?object=PWY3O-123) |
| 12 | [superpathway of serine and glycine biosynthesis](http://pathway.yeastgenome.org/YEAST/NEW-IMAGE?object=PWY3O-261) | 64 | [trehalose biosynthesis](http://pathway.yeastgenome.org/YEAST/NEW-IMAGE?object=TRESYN-PWY) |
| 13 | [superpathway of sulfur amino acid biosynthesis](http://pathway.yeastgenome.org/YEAST/NEW-IMAGE?object=PWY-821) | 65 | [chitin biosynthesis](http://pathway.yeastgenome.org/YEAST/NEW-IMAGE?object=PWY3O-15) |
| 14 | [S-adenosylmethionine cycle](http://pathway.yeastgenome.org/YEAST/NEW-IMAGE?object=PWY-5041) | 66 | [superpathway of TCA cycle and glyoxylate cycle](http://pathway.yeastgenome.org/YEAST/NEW-IMAGE?object=PWY3O-94) |
| 15 | [superpathway of threonine and methionine biosynthesis](http://pathway.yeastgenome.org/YEAST/NEW-IMAGE?object=P4-PWY) | 67 | [pentose phosphate pathway](http://pathway.yeastgenome.org/YEAST/NEW-IMAGE?object=PENTOSE-P-PWY) |
| 16 | [superpathway of methionine biosynthesis](http://pathway.yeastgenome.org/YEAST/NEW-IMAGE?object=PWY3O-954) | 68 | [aerobic respiration, electron transport chain](http://pathway.yeastgenome.org/YEAST/NEW-IMAGE?object=PWY3O-188) |
| 17 | [superpathway of glutamate biosynthesis](http://pathway.yeastgenome.org/YEAST/NEW-IMAGE?object=GLUTSYN-PWY) | 69 | [glycolysis](http://pathway.yeastgenome.org/YEAST/NEW-IMAGE?object=GLYCOLYSIS) - [superpathway of glucose fermentation](http://pathway.yeastgenome.org/YEAST/NEW-IMAGE?object=GLUCFERMEN-PWY) |
| 18 | [methionine salvage pathway](http://pathway.yeastgenome.org/YEAST/NEW-IMAGE?object=PWY3O-64) | 70 | [superpathway of acetoin and butanediol biosynthesis](http://pathway.yeastgenome.org/YEAST/NEW-IMAGE?object=PWY3O-981) |
| 19 | [proline biosynthesis](http://pathway.yeastgenome.org/YEAST/NEW-IMAGE?object=PROSYN-PWY) | 71 | [fatty acid oxidation pathway](http://pathway.yeastgenome.org/YEAST/NEW-IMAGE?object=YEAST-FAO-PWY) |
| 20 | [ergosterol biosynthesis](http://pathway.yeastgenome.org/YEAST/NEW-IMAGE?object=ERGOSTEROL-SYN-PWY) | 72 | [formaldehyde oxidation II (glutathione-dependent)](http://pathway.yeastgenome.org/YEAST/NEW-IMAGE?object=PWY-1801) |
| 21 | [myristate biosynthesis](http://pathway.yeastgenome.org/YEAST/NEW-IMAGE?object=PWY-5971-1) | 73 | [phospholipids degradation](http://pathway.yeastgenome.org/YEAST/NEW-IMAGE?object=LIPASYN-PWY-1) |
| 22 | [fatty acid biosynthesis, initial steps](http://pathway.yeastgenome.org/YEAST/NEW-IMAGE?object=PWY3O-10) (saturated and unsaturated) | 74 | arginine degradation ([proline utilization](http://pathway.yeastgenome.org/YEAST/NEW-IMAGE?object=PROUT-PWY)) |
| 23 | fatty acid biosynthesis, elongation (saturated and unsaturated) | 75 | [lysine degradation](http://pathway.yeastgenome.org/YEAST/NEW-IMAGE?object=LYSDEGII-PWY) |
| 24 | [phosphatidylinositol phosphate biosynthesis](http://pathway.yeastgenome.org/YEAST/NEW-IMAGE?object=PWY3O-242) | 76 | [leucine degradation](http://pathway.yeastgenome.org/YEAST/NEW-IMAGE?object=PWY3O-4112) |
| 25 | [fatty acid biosynthesis (concise)](http://pathway.yeastgenome.org/YEAST/NEW-IMAGE?object=PWY-5970) | 77 | [threonine degradation](http://pathway.yeastgenome.org/YEAST/NEW-IMAGE?object=THREOCAT2-PWY) |
| 26 | [sphingolipid metabolism](http://pathway.yeastgenome.org/YEAST/NEW-IMAGE?object=SPHINGOLIPID-SYN-PWY) | 78 | [valine degradation](http://pathway.yeastgenome.org/YEAST/NEW-IMAGE?object=PWY3O-4105) |
| 27 | [palmitate biosynthesis](http://pathway.yeastgenome.org/YEAST/NEW-IMAGE?object=PWY3O-8514) | 79 | [isoleucine degradation](http://pathway.yeastgenome.org/YEAST/NEW-IMAGE?object=PWY3O-4109) |
| 28 | [fatty acid biosynthesis, initial steps](http://pathway.yeastgenome.org/YEAST/NEW-IMAGE?object=PWY3O-10) ([saturated](http://pathway.yeastgenome.org/YEAST/NEW-IMAGE?object=PWY3O-6336)) | 80 | [glutamate degradation I](http://pathway.yeastgenome.org/YEAST/NEW-IMAGE?object=GLUDEG-I-PWY) |
| 29 | fatty acid biosynthesis, elongation ([saturated](http://pathway.yeastgenome.org/YEAST/NEW-IMAGE?object=PWY3O-6336)) | 81 | [tryptophan degradation](http://pathway.yeastgenome.org/YEAST/NEW-IMAGE?object=PWY3O-214) |
| 30 | [phospholipid biosynthesis](http://pathway.yeastgenome.org/YEAST/NEW-IMAGE?object=PHOSLIPSYN2-PWY) | 82 | [phenylalanine degradation](http://pathway.yeastgenome.org/YEAST/NEW-IMAGE?object=PWY3O-4115) |
| 31 | [triglyceride biosynthesis](http://pathway.yeastgenome.org/YEAST/NEW-IMAGE?object=TRIGLSYN-PWY) | 83 | [tyrosine degradation](http://pathway.yeastgenome.org/YEAST/NEW-IMAGE?object=PWY3O-4108) |
| 32 | [superpathway of phosphatidate biosynthesis](http://pathway.yeastgenome.org/YEAST/NEW-IMAGE?object=PWY3O-6635) | 84 | [asparagine degradation](http://pathway.yeastgenome.org/YEAST/NEW-IMAGE?object=ASPARAGINE-DEG2-PWY) |
| 33 | [glycerol biosynthesis](http://pathway.yeastgenome.org/YEAST/NEW-IMAGE?object=PHOSLIPSYN2-PWY) | 85 | [glycine cleavage complex](http://pathway.yeastgenome.org/YEAST/NEW-IMAGE?object=GLYCLEAV-PWY) |
| 34 | [very long chain fatty acid biosynthesis](http://pathway.yeastgenome.org/YEAST/NEW-IMAGE?object=PWY-5080) | 86 | [2-ketoglutarate dehydrogenase complex](http://pathway.yeastgenome.org/YEAST/NEW-IMAGE?object=PWY-5084) |
| 35 | [superpathway of phosphatidic acid and phospholipid biosynthesis](http://pathway.yeastgenome.org/YEAST/NEW-IMAGE?object=PHOS-PWY) | 87 | [L-serine degradation](http://pathway.yeastgenome.org/YEAST/NEW-IMAGE?object=SERDEG-PWY) |
| 36 | [folate biosynthesis II](http://pathway.yeastgenome.org/YEAST/NEW-IMAGE?object=PWY3O-45) | 88 | [glutamate degradation IX](http://pathway.yeastgenome.org/YEAST/NEW-IMAGE?object=PWY-3322) |
| 37 | [superpathway of heme and siroheme biosynthesis](http://pathway.yeastgenome.org/YEAST/NEW-IMAGE?object=PWY3O-69) | 89 | [alanine degradation](http://pathway.yeastgenome.org/YEAST/NEW-IMAGE?object=ALANINE-DEG3-PWY) |
| 38 | [riboflavin, FMN and FAD biosynthesis](http://pathway.yeastgenome.org/YEAST/NEW-IMAGE?object=YEAST-RIBOSYN-PWY) | 90 | [galactose degradation](http://pathway.yeastgenome.org/YEAST/NEW-IMAGE?object=YEAST-GALACT-METAB-PWY) |
| 39 | [thioredoxin system](http://pathway.yeastgenome.org/YEAST/NEW-IMAGE?object=THIOREDOX-PWY) | 91 | [mannose degradation](http://pathway.yeastgenome.org/YEAST/NEW-IMAGE?object=PWY3O-1743) |
| 40 | [superpathway of NAD biosynthesis](http://pathway.yeastgenome.org/YEAST/NEW-IMAGE?object=PWY3O-4158) | 92 | [methylglyoxal catabolism](http://pathway.yeastgenome.org/YEAST/NEW-IMAGE?object=PWY-901) |
| 41 | [folate polyglutamylation](http://pathway.yeastgenome.org/YEAST/NEW-IMAGE?object=PWY3O-20) | 93 | [glycogen catabolism](http://pathway.yeastgenome.org/YEAST/NEW-IMAGE?object=GLYCOCAT-YEAST-PWY) |
| 42 | [folate interconversions](http://pathway.yeastgenome.org/YEAST/NEW-IMAGE?object=PWY3O-697) | 94 | [xylose metabolism](http://pathway.yeastgenome.org/YEAST/NEW-IMAGE?object=PWY3O-8) |
| 43 | [biotin biosynthesis](http://pathway.yeastgenome.org/YEAST/NEW-IMAGE?object=BIOTIN-SYNTHESIS-PWY) | 95 | [xylulose degradation](http://pathway.yeastgenome.org/YEAST/NEW-IMAGE?object=PWY3O-5) |
| 44 | [folate transformations](http://pathway.yeastgenome.org/YEAST/NEW-IMAGE?object=PWY-2201) | 96 | [fructose degradation](http://pathway.yeastgenome.org/YEAST/NEW-IMAGE?object=PWY3O-0) |
| 45 | [superpathway of glutathione metabolism (truncated γ-glutamyl cycle)](http://pathway.yeastgenome.org/YEAST/NEW-IMAGE?object=PWY3O-114) | 97 | [trehalose degradation](http://pathway.yeastgenome.org/YEAST/NEW-IMAGE?object=TREDEG-YEAST-PWY) |
| 46 | [pyridoxal 5'-phosphate salvage pathway](http://pathway.yeastgenome.org/YEAST/NEW-IMAGE?object=PLPSAL-PWY) | 98 | [sucrose degradation](http://pathway.yeastgenome.org/YEAST/NEW-IMAGE?object=SUCUTIL-PWY) |
| 47 | [glutathione-glutaredoxin system](http://pathway.yeastgenome.org/YEAST/NEW-IMAGE?object=PWY3O-592) | 99 | [pyruvate dehydrogenase complex](http://pathway.yeastgenome.org/YEAST/NEW-IMAGE?object=PYRUVDEHYD-PWY) |
| 48 | [glutathione-glutaredoxin redox reactions](http://pathway.yeastgenome.org/YEAST/NEW-IMAGE?object=GLUT-REDOX2-PWY) | 100 | [allantoin degradation](http://pathway.yeastgenome.org/YEAST/NEW-IMAGE?object=ALLANTOINDEG-PWY) |
| 49 | [superpathway of purine biosynthesis and salvage pathways](http://pathway.yeastgenome.org/YEAST/NEW-IMAGE?object=PWY3O-285) | 101 | [4-aminobutyrate degradation](http://pathway.yeastgenome.org/YEAST/NEW-IMAGE?object=YEAST-4AMINOBUTMETAB-PWY) |
| 50 | [de novo biosynthesis of pyrimidine ribonucleotides](http://pathway.yeastgenome.org/YEAST/NEW-IMAGE?object=PYRIMID-RNTSYN-PWY) | 102 | [glycerol degradation](http://pathway.yeastgenome.org/YEAST/NEW-IMAGE?object=AERO-GLYCEROL-CAT-PWY) |
| 51 | [de novo biosynthesis of pyrimidine deoxyribonucleotides](http://pathway.yeastgenome.org/YEAST/NEW-IMAGE?object=YEAST-DE-NOVO-PYRMID-DNT) | 103 | [butanediol degradation](http://pathway.yeastgenome.org/YEAST/NEW-IMAGE?object=PWY3O-246) |
| 52 | [salvage pathways of purines and their nucleosides](http://pathway.yeastgenome.org/YEAST/NEW-IMAGE?object=PWY3O-1) |  |  |
